# Matrix Product Operator Algebras I: Representations of Weak Hopf Algebras and Projected Entangled Pair States

**DOI:** 10.1007/s00220-026-05670-w

**Published:** 2026-08-28

**Authors:** Andras Molnar, Alberto Ruiz-de-Alarcón, José Garre-Rubio, Norbert Schuch, J. Ignacio Cirac, David Pérez-García

**Affiliations:** 1 Faculty of Mathematics, University of Vienna, Oskar-Morgenstern-Platz 1, 1090 Vienna, Austria; 2 Dpto. de Análisis Matemático y Matemática Aplicada, Universidad Complutense de Madrid, Plaza de las Ciencias 3, 28040 Madrid, Spain; 3 Instituto de Ciencias Matemáticas, Nicolás Cabrera 13-15, 28049 Madrid, Spain; 4 Faculty of Physics, University of Vienna, Boltzmanngasse 5, 1090 Vienna, Austria; 5 Max-Planck-Institute of Quantum Optics, Hans-Kopfermann-Str. 1, 85748 Garching, Germany; 6 Munich Center for Quantum Science and Technology, Schellingstr. 4, 80799 München, Germany

## Abstract

Matrix Product Operators (MPOs) are tensor networks representing operators acting on 1D systems. They model a wide variety of situations, including communication channels with memory effects, quantum cellular automata, mixed states in 1D quantum systems, or holographic boundary models associated to 2D quantum systems. A scenario where MPOs have proven particularly useful is to represent algebras of non-trivial symmetries. Concretely, the boundary of both symmetry protected and topologically ordered phases in 2D quantum systems exhibit symmetries in the form of MPOs. In this paper, we develop a theory of MPOs as representations of algebraic structures. We establish a dictionary between algebra and MPO properties which allows to transfer results between both setups, covering the cases of pre-bialgebras, weak bialgebras, and weak Hopf algebras. We define the notion of pulling-through algebras, which abstracts the minimal requirements needed to define topologically ordered 2D tensor networks from MPO algebras. We show, as one of our main results, that any semisimple pivotal weak Hopf algebra is a pulling-through algebra. We demonstrate the power of this framework by showing that they can be used to construct Kitaev’s quantum double models for Hopf algebras solely from an MPO representation of the Hopf algebra, in the exact same way as MPO symmetries obtained from fusion categories can be used to construct Levin-Wen string-net models, and to explain all their topological features; it thus allows to describe both Kitaev and string-net models on the same formal footing.

## Introduction

### Context

Tensor Networks [ONU22, CPSV21, Hac19] allow to express certain high-dimensional tensors $T_{i_1,\dots ,i_N}$—that is, tensors with a large number *N* of indices—efficiently as a network of simple few-index tensors. Those elementary tensors are arranged on a graph (“network”), and their “auxiliary” indices are contracted (i.e., identified and summed over) with those of the adjacent tensor, as prescribed by the edges of the graph. The resulting object describes a multi-dimensional tensor whose indices are given by the remaining uncontracted indices of the original network. This allows to express the original tensor $T_{i_1,\dots ,i_N}$ with its exponential number $d^N$ of parameters (for simplicity, $i_k=1,\dots ,d$ for all *k*) through a much smaller number of parameters (typically scaling linearly in *N*), while retaining highly non-trivial correlations between the different indices of *T*. Surprisingly, in a remarkably broad range of applications, this exponential saving in the number of parameters effectively does not restrict the expressive power of the ansatz.

A particularly successful instance of tensor networks is given by one-dimensional (1D) tensor networks, most commonly known by the names of Matrix Product States (MPS) [PVWC07, FNW92] or Tensor Trains (TT) [Ose11], among others. These ansatzes have been independently (re-)discovered in different fields and have found applications in a multitude of areas throughout data science and physics. This includes, for instance, their use in modeling and compressing high-dimensional data, such as for image compression or the simulation of PDEs, as well as in the design of deep learning algorithms, such as for hidden Markov models or Born machines [SS17, NTO17, CLO+16, CPZ+17, Lat05, ME22, IOL07, Gar21, BSU16, CCC+19, GSP+19]. In physics, Tensor Networks—both one-dimensional MPS and their higher-dimensional generalization, termed Projected Entangled Pair States (PEPS)—have proven to be a powerful ansatz for the simulation and modeling of quantum many-body systems in a wide range of scenarios in condensed matter, atomic, and high energy physics, as well as in quantum chemistry [Sch11, SC12, MG02, DM16, PKS+19, ZCC+17, Oru19, VCM08, SSV+05, BC20, PYHP15, JE21, SPM+15, OHN+15]. Notably, their suitability for faithfully approximating low-energy states of local Hamiltonians can be rigorously proven in a broad range of settings [Has07b, Has07a, CPSV21], providing a formal justification of their success in the study of such systems; and the fact that MPS allow to represent physical symmetries locally [PWS+08]—that is, as acting on the auxiliary degrees of freedom—has enabled a comprehensive classification of unconventional phases under symmetries [CGW11, SPC11, CPSV21].

A generalization of MPS which is natural in many contexts are Matrix Product Operators (MPOs) [ZV04]. MPOs describe operators acting on a multi-dimensional tensor which themselves can be expressed as 1D tensor networks and thus possess—and preserve—the underlying 1D locality structure. On the one hand, such MPOs can encode operations or transformations which themselves have a 1D structure; they thus form natural generalization of strictly local operations, in that they preserve the local correlation structure when acting on an MPS [MCPV10, HV17, CPSV17b, ŞCSBC18]. On the other hand, MPOs can characterize symmetries acting on a system which have an intrinsic 1D correlation structure [RVHB19, CLV22], or by implication projections onto subspaces invariant under the symmetry which can exhibit highly non-trivial structures [GLM23]. Notably, in both cases, despite their local form the structure of such operators can be fundamentally different from what can be achieved by low-depth local circuits alone.

MPOs appear most naturally in the context of quantum many-body problems. They can, for instance, describe evolutions with a local structure (but not necessarily locally generated) such as in driven quantum systems, i.e., Floquet physics [PFM+16]; and they can describe the symmetries of chains of particles with anyonic statistics [FTL+07, GAT+13, GLM23]. However, the use of MPOs in many-body physics goes far beyond this and extends deeply into the study of 2D systems: Boundaries—and in particular the correlations across those boundaries, i.e., the entanglement spectrum—play a key role in understanding the physics of strongly correlated quantum matter [LH08, Laf16]. The boundary of a 2D quantum system has, yet again, a natural 1D structure, which can be made explicit by cutting a 2D tensor network description of the bulk system at the boundary [CPSV11]. The density matrix which carries the entanglement spectrum can then be described by an MPO at the boundary. On the other hand, just as physical symmetries in MPS can be represented as acting at the auxiliary indices, symmetries in 2D PEPS can be understood as acting as MPOs at the 1D boundary [CLW11, WBM+16, MGSC18], in which case they naturally form a representation of the symmetry group; indeed, the classification of MPO representations of groups enabled the classification of symmetry protected phases in 2D. However, there is another way in which such symmetries can act: Rather than acting as faithful representations both on the physical and the auxiliary degree of freedom, MPO symmetries can also appear as symmetries of the auxiliary degrees of freedom on their own, that is, as pure entanglement symmetries. These entanglement symmetries are precisely what underlies topological order in 2D, and they allow to comprehensively understand topologically ordered systems, encompassing their ground space structure as well as anyons and their braiding [SCP10, BMW+17, CPSV21] (see Sect. 1.3 for a detailed discussion).

In many of these cases, MPOs naturally form algebraic structures, such as group representations in the case of symmetries or evolutions. A particularly strong structure arises in the case where the MPO on its own describes a symmetry, such as the entanglement symmetries which appear in topologically ordered systems: As both products and linear combinations of symmetries are again symmetries, the MPOs which appear in topologically ordered systems naturally form MPO algebras. Given the widespread use of MPS and MPOs, the importance of understanding topologically ordered systems, and the key role played by MPO algebras in this context, it is thus highly desirable to formalize the representation theory of MPO algebras and their underlying algebraic structure, and to understand the way in which additional conditions imposed on the corresponding algebraic structures are reflected in properties of their MPO representation, and vice versa [BMW+17, Kaw20, Kaw21a, Kaw21b].

### Main results of this work

In this paper, we lay out a framework for describing MPOs as (faithful) representations of algebraic structures $\mathcal {A}$ which are both algebras and co-algebras (i.e. duals of an algebra), with certain compatibility conditions: pre-bialgebras, weak bialgebras, weak Hopf algebras, and variants thereof, and we establish a dictionary between the properties of those algebraic structures $\mathcal {A}$ and those of their MPO representations. This framework allows one to use the algebraic structure of $\mathcal {A}$ to reason about MPOs, and conversely to use MPOs to derive statements about $\mathcal {A}$. We then show that under suitable conditions, those structures—and thus their MPO representations—satisfy a condition which we use to define a new algebraic structure: *pulling-through algebras*; those algebras play a key role in the construction and study of topologically ordered models from weak Hopf algebras. We conclude by demonstrating how pulling-through algebras show up in the construction of a large class of topological models; the study of further applications in topological order is left for future work.

Let us describe more specifically the key results of the work. We start by showing that MPS naturally appear as representations of coalgebras (i.e. duals of algebras) in Sect. 3. MPOs are then representations of pre-bialgebras $\mathcal {A}$ – coalgebras which are also algebras, with a minimal compatibility condition (Sect. 4). In both cases, the coalgebra element $a\in \mathcal {A}$ is encoded in the boundary condition of the MPO or MPS. We then show that semisimplicity of $\mathcal {A}^*$, the dual of $\mathcal {A}$, amounts to an MPS/MPO which decomposes into irreducible (i.e., normal/injective) blocks corresponding to the irreps of $\mathcal {A}^*$—importantly, for MPOs these irreducible blocks give rise to a notion of sectors and their fusion, which links to topological order—and that cocentral elements are represented by translational invariant MPOs.

We then study the effect of introducing the additional conditions which make $\mathcal {A}$ a weak bialgebra (WBA) or weak Hopf algebra (WHA), respectively (Sect. 5). We show how those conditions give rise to additional properties of the sectors of the MPO and their fusion, giving them the structure of a monoidal category in the case of a WBA and multi-fusion category in the case of a WHA. In particular, we show how to construct a “vaccum” sector, as well as an integral whose representation is a special projector (used later to construct the topological models). By further restricting to pivotal, spherical, and $C^*$ weak Hopf algebras, we then use said integral to show the existence of a “pulling through structure” satisfying a sequence of increasingly strong conditions. This motivates the definition of pulling-through algebras in Sect. 6, whose MPO representation possesses such a pulling through structure which satisfies the corresponding conditions. In Sect. 7, we construct PEPS based on pulling-through algebras, show that their symmetry structure is scale invariant—central for follow-up work – and demonstrate that in the case of $C^*$ Hopf algebras, the resulting models are equivalent to the class of generalized Kitaev models, discussed in Sect. 1.3 below. Finally in Sect. 8 we discuss future directions of our framework and explain how the present approach can be applied to various physical scenarios such as in Refs. [RGMP24, Kat24, LRS+25, Sun25, OPR25, LMS+25].

This work is the first of a series of papers in which we utilize MPO algebras to understand and classify topological phases of matter in quantum many body systems. The second paper [RGMP24] is concerned with classifying phases in 1D open quantum systems as equivalence classes of shallow circuits. At the heart of this problem is the classification of renormalization fixed point density operators, and the present work provides the tools required to build examples for fixed point MPOs, as well as channels that map between such states. The third paper of the series studies the structure of PEPS representations of topological models constructed from pulling-through algebras, as explained above. This is in particular relevant for characterizing the representation of physical symmetries on the auxiliary degrees of freedom, and thus the classification of symmetry-enriched topological phases.

### Connection to topological order

As mentioned before, MPO algebras are especially important in the study of topologically ordered phases in 2D tensor networks, i.e., PEPS. In particular, the definition of pulling-through algebras in this work is directly motivated by the study of topological order in PEPS. In the following, we explain this context, and the way in which our results fit in, in more detail.

Topologically ordered phases are phases which exhibit order which cannot be detected by any local order parameter. Instead, they are characterized by a global ordering in their quantum correlations, also known as entanglement. Characteristic to these systems are their degenerate ground states, which are locally indistinguishable and whose number depends on the topology of the surface on which the system is defined—both incompatible with local order parameters – as well as the presence of excitations with non-trivial statistics in the system, termed “anyons”.

In a seminal work, Kitaev [Kit03] first proposed a Hamiltonian model for a spin system with the aforementioned properties, the Toric Code model, as well as its generalization to finite groups *G*, the quantum double models. An alternative construction was provided by the string-net models of Levin and Wen [LW05]. While string-net models can be understood as another way of generalizing the Toric Code model, they are in fact motivated by topological quantum field theories. Hence, they use a category theoretical language (the construction utilizes unitary fusion categories with some additional restrictions—the so-called tetrahedral symmetry of the *F*-symbols), as opposed to the algebraic approach used for the quantum double models (which are constructed as representations of the quantum double *D*(*G*)). Over the years, generalizations of both classes of models have been devised. Kitaev already noted in his original work [Kit03] that the same construction also works for semisimple Hopf algebras. This has later been worked out in detail [BK12, BMCA13], and further generalized to $C^*$-weak Hopf algebras [Cha14]. For string-net models, it has been shown that the requirement of tetrahedral symmetry can be dropped [HW20], and thus string-net models can be built from arbitrary unitary fusion categories; further, the construction has been generalized to build on bimodule categories instead of fusion categories [LFH+20].

Both Kitaev models and string-net models admit tensor network descriptions. Such a description has been developed first for Kitaev’s Toric Code model [VWPC06] and later generalized to Kitaev models based on finite groups [SCP10] and Hopf algebras [BMCA13], and separately for string-net models [BAV09, GLSW09]. A characteristic feature of the PEPS description both of Kitaev models based on finite groups and of string-net models is that the tensors which define the state possess symmetries which act solely on the auxiliary degrees of freedom of the tensor. This symmetry is intimately tied to the topological features of the system, as it allows to explain both its ground space degeneracy and the presence of anyonic excitations [SCP10, ŞCWB+14, BMW+17], whereas breaking it even slightly leads to an immediate breakdown of topological order in 2D [CZG+10] (but not in 3D [WDVS21, DS21]). A crucial property of these symmetries is their size-independence: They are given either by tensor powers of a local symmetry generator (for the double models) or, more generally, by homogenous MPOs (for string-net models), such that every region in the PEPS possesses the same MPO symmetries. The specific properties of the underlying topological phase can then be inferred by studying the algebraic properties of the corresponding MPO algebra. Remarkably, it is even possible to build topological models in this very phase from nothing but the MPO symmetry itself: the PEPS tensor is then constructed from the MPO by placing it on a fixed-size ring with suitable boundaries [SCP10, BMW+17, CPSV21].

Despite the success of MPO symmetries in understanding, characterizing, and simulating topological order in the phases of the Kitaev double models of finite groups and of the string-net models [SCP10, BWV+18, LCSV22, DIH+17], the picture is unfortunately not complete. First off, for the Kitaev model based on Hopf algebras the known tensor network constructions [BMCA13] do not evidently display any such symmetries; and moving to an even broader setting, for the Kitaev models constructed from weak Hopf algebras not even a tensor network description is known—which, in turn, should be possible to construct once the underlying MPO symmetries have been identified. This lack of knowledge is the more surprising given that weak Hopf algebras correspond to multi-fusion categories [EGH+], and thus, they exhibit the same type of topological order as the corresponding string-net models. A key reason why, despite this connection, an understanding of the MPO symmetries underlying Kitaev models for (weak) Hopf algebras is missing is the fact that the MPO symmetries for string-net models are constructed in a category theoretical language. To understand the MPO symmetries relevant for describing Kitaev models, however, an algebraic language is clearly more natural. This is precisely what we achieve in this work: We show that weak Hopf algebras correspond to MPO algebras with specific properties, most importantly the pulling-through structure. Using these MPO algebras, we can then construct a PEPS representation for Kitaev models based on any weak Hopf algebra; we explicitly show the connection between both representations for the case where the MPOs are built from a $C^*$-Hopf algebra.

On a more abstract level, the relation between the MPO symmetries of the string-net models and the MPOs representations of semisimple weak Hopf algebras is as follows. The MPOs constructed in this work are representations of semisimple weak Hopf algebras. In turn, representations of semisimple weak Hopf algebras are known to be exactly multi-fusion categories [EGH+]. Vice versa, every (multi-)fusion category arises as the category of representations of some weak Hopf algebra. The MPO symmetries of the string-net model based on any given fusion category will thus form a representation of the corresponding weak Hopf algebra. In fact, that weak Hopf algebra can be constructed from the MPO symmetries themselves, when closed with arbitrary boundary conditions. It is important to note that while (ordinary) string-net models are built on a single fusion category, general representations of weak Hopf algebras involve two different fusion categories: one is the representation category of the weak Hopf algebra, the other is the category (or a subcategory) of the representations of the dual weak Hopf algebra. The correspondence between fusion categories, MPOs, and weak Hopf algebras then suggests that string-net models based on bimodule categories [LFH+20] and PEPS constructed from weak Hopf algebras are actually the same. The exact details on how to map these models to each other is left for future work.

### Structure of the paper

This paper is structured as follows. First, in Sect. 2 we introduce the graphical notation of tensor calculus used throughout the paper. In Sect. 3 we introduce coalgebras and matrix product states (MPS), and establish the relation between them: one can think of an MPS as a representation of a coalgebra. We specialize to the case where the coalgebra is cosemisimple; in this case the representing MPS is in canonical form, i.e. it is a sum of smaller bond dimensional injective MPS. In Sect. 4 we investigate pre-bialgebras: coalgebras that are algebras as well such that the two structures satisfy a compatibility condition. Correspondingly, the previously defined MPS representations of pre-bialgebras will become MPOs and these MPOs are closed under multiplication. In Sect. 5 we introduce weak bialgebras (pre-bialgebras such that their unit and counit satisfy certain properties) and weak Hopf algebras (weak bialgebras with an extra operation called the antipode). The main result of this section is Theorem 5.1, which proves the above mentioned special integral in cosemisimple WHAs over $\mathbb {C}$. In Sect. 5.3 we specialize to pivotal WHAs, which, as mentioned above, allows us to define a “pulling-through” structure on the WHA. In Sect. 5.5 we further specialize to $C^*$-WHAs, which guarantees extra properties of this pulling-through structure. In Sect. 6 we investigate pulling-through algebras independently from the previous weak Hopf algebra properties. We develop a graphical language suited for these algebras that we then use in Sect. 7 to define PEPS that have stable entanglement symmetries described by this pulling-through algebra, and which we show to precisely describe Kitaev’s quantum double models in the case of Hopf algebras. Finally in Sect. 8 we provide possible physical applications of our results.

## Graphical Notation of Tensor Calculus

In this section we introduce the graphical notation of tensor calculus that we use throughout the paper. This graphical notation is especially useful to visualize equations that involve the contraction of many higher rank tensors (i.e. tensors with more than two indices) and it is standard in the field of tensor networks. In this paper, however, we face extra challenges as we use the graphical language parallel to an algebraic one, and thus we have to modify the usual graphical language in order to be able to translate between the two languages.

From a computational point of view, tensors are just multi-dimensional arrays. In the usual graphical notation of tensor calculus, one denotes tensors by dots (and various shapes) with lines connected to them. The number of lines connected to the dot (or other shape) is the rank of the tensor, and each line corresponds to one of the vector spaces in the tensor product. For example, the following diagrams represent a scalar, a vector and a matrix, respectively:

**Figure:**
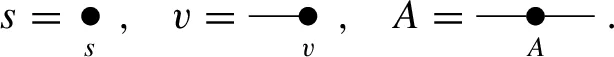

Tensor contraction is denoted by joining the lines corresponding to the contracted indices of the two tensors. For example the scalar product of two vectors, a matrix acting on a vector and the product of two matrices are denoted by the following diagrams, respectively:

**Figure:**
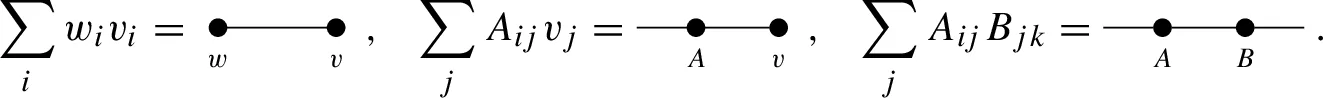

Here we have made the implicit assumption that the first index is the left line, the second one is the right one. For higher rank tensors and more complicated contraction schemes one has to keep track of which index belongs to which line (by e.g. fixing a convention such as in the previous figure). In the following, we outline a notation that allows us to distinguish the different indices of the tensor without requiring them to be always at the same position. This notation is thus suitable to depict more complicated tensor constructions such as the definition of a PEPS.

To introduce our modification of the graphical notation, let us first formalize what tensors and tensor contractions are. Rank-*n* tensors are elements of the tensor product $V_1 \otimes \dots \otimes V_n$ for some finite dimensional vector spaces $V_1 \dots V_n$. One can naturally take tensor products of tensors: for example, if $V_1$, $V_2$ and $V_3$ are vector spaces and $r = v_1 \otimes v_2 \in V_1 \otimes V_2$ and $s=v_3\otimes f \in V_3\otimes V_2^*$, where $V_2^*$ denotes the space of linear functionals on $V_2$, then their tensor product t=r⊗s is $t=v_1 \otimes v_2 \otimes v_3 \otimes f$. Tensor contraction (without introducing a scalar product) is then the following operation: if amongst the *n* components of the tensor product both a vector space *V* and its dual $V^*$ appears, then one can form a rank-(n-2) tensor by acting with the linear functional in $V^*$ on the vector in *V*. For example, the tensor $t=v_1 \otimes v_2 \otimes v_3 \otimes f$ defined above is an element of the space $V_1 \otimes V_2 \otimes V_3 \otimes V_2^*$, and thus one can contract its second and fourth components to obtain a rank-two tensor $\mathcal {C}_{24}(t) = f(v_2) \cdot v_1 \otimes v_3 \in V_1 \otimes V_3$.

As we have seen, to make sense of tensor contraction without a scalar product, it is important to differentiate between vector spaces and linear functionals. We will denote indices corresponding to vectors by outgoing arrows, while indices corresponding to linear functionals by incoming arrows. Moreover, to distinguish between the different indices or the tensor, we will label the lines with vector spaces. For outgoing arrow, the label is the corresponding tensor component. For incoming arrow, the label is the vector space the given tensor component is the dual of. For example, a vector v∈V, a linear functional $f\in V^*$ and a rank-two tensor $A\in V\otimes V^*$ is denoted by

**Figure:**
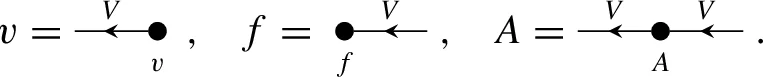

Tensor contraction is still denoted by joining lines. Note, however, that now only lines with the same label can be joined that also point in the same direction. For example,

**Figure:**
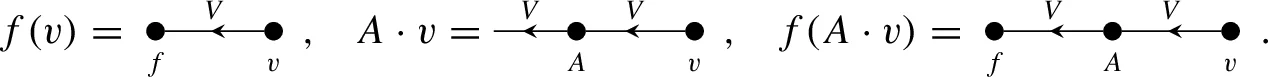

The vector space *V* is canonically isomorphic to $$V^{**}$$, and thus one can equally think of *V* as the space of linear functionals on $$V^*$$. Correspondingly, in our graphical notation every arrow can be reversed by changing the label from *V* to $$V^*$$. For example, there are four different ways to depict a rank-two tensor $$A\in V\otimes V^*$$:

**Figure:**
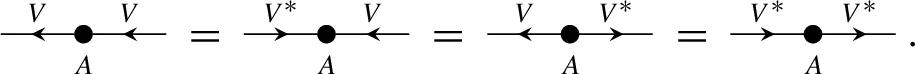

The first depiction of *A* suggests to interpret it as a linear map $\hat{A}:V \rightarrow V$, while the last equation suggests to interpret it as a linear map $V^* \rightarrow V^*$; this linear map is $\hat{A}^T$. Such rank-two tensors are sometimes labeled by the linear map $\hat{A}$; when this is the case, we will try to be consistent and label the vector spaces by *V* and not by $V^*$.

As we will depict tensor networks in two dimensions, sometimes it will be convenient to rotate tensors. This means that vectors do not always point to the left, and thus we actually need the arrows to distinguish between the two indices of a rank-two tensor. Such a rotation is, for example, the following:

**Figure:**
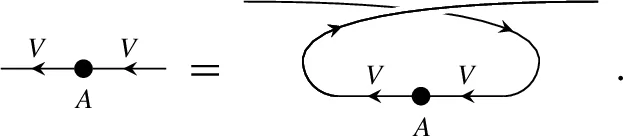

In the rest of the paper we will often deal with rank-three and rank-four tensors of the form $A \in V \otimes W \otimes W^*$ and $O \in V\otimes V^* \otimes W \otimes W^*$. These tensors are denoted by

**Figure:**
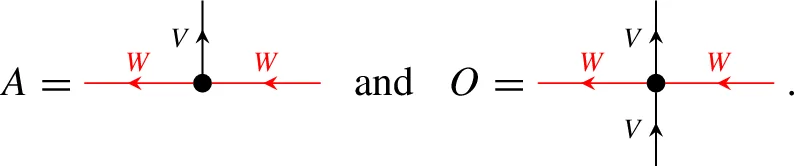

For better visual distinction, we have introduced colors: the red colored edges are labeled by *W* and the black colored edges by *V*. In fact, in cases where the vector spaces *V* and *W* are fixed and we only need to distinguish between $V,V^*,W$ and $W^*$, we will drop the labels and keep the colors only, denoting *A* and *O* by

**Figure:**
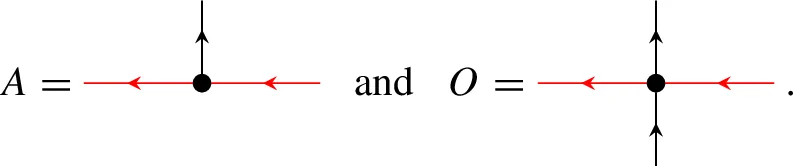

## Coalgebras and Matrix Product States

In this section we define coalgebras and matrix product states and show that MPS can be thought of as representations of coalgebras. We also show that this correspondence holds the other way around as well: given an MPS tensor, one can construct a coalgebra such that the MPS forms a representation of the constructed coalgebra. This observation makes thus MPS and coalgebras completely equivalent. We elaborate on a special case: when the coalgebra is cosemisimple, the corresponding MPS tensor is a sum of injective tensors (see Definition 3.3), and vice versa, given an MPS that is a sum of injective tensors, the constructed coalgebra is cosemisimple. We also define the notion of cocentral and non-degenerate coalgebra elements and show how these properties are reflected in the MPS representation.

We start by defining coalgebras.

### Definition 3.1

*(Coalgebra)*. The triple $(\mathcal {C},\Delta ,\epsilon )$ is a *coalgebra* if $\mathcal {C}$ is a finite dimensional vector space over $\mathbb {C}$, $\Delta :\mathcal {C}\rightarrow \mathcal {C}\otimes \mathcal {C}$ is a linear map called comultiplication such that it is associative:

$$\begin{aligned} (\Delta \otimes \textrm{Id}) \circ \Delta = (\textrm{Id}\otimes \Delta ) \circ \Delta , \end{aligned}$$

and $\epsilon \in \mathcal {C}^*$ is a linear functional called *counit*, such that

$$\begin{aligned} (\epsilon \otimes \textrm{Id})\circ \Delta = (\textrm{Id}\otimes \epsilon )\circ \Delta = \textrm{Id}. \end{aligned}$$

Coalgebras emerge as the dual of algebras: Given a finite dimensional algebra $\mathcal {A}$ with product $\mu _{\mathcal {A}}:\mathcal {A}\otimes \mathcal {A}\rightarrow \mathcal {A}$ and unit 1, we can define a coproduct on $\mathcal {A}^*$ by defining $\Delta _{\mathcal {A}^*}:\mathcal {A}^*\rightarrow \mathcal {A}^*\otimes \mathcal {A}^*$ as $\Delta _{\mathcal {A}^*} = \mu _{\mathcal {A}}^T$, that is, by

$$\begin{aligned} \Delta _{\mathcal {A}^*}(f)(x\otimes y) = f(xy), \end{aligned}$$

where $x,y\in \mathcal {A}$ and $f\in \mathcal {A}^*$: Associativity of $\Delta _{\mathcal {A}^*} = \mu _{\mathcal {A}}^T$ is equivalent to the associativity of $\mu _{\mathcal {A}}$, and the map given by f↦f(1) defines the counit of $\mathcal {A}^*$. Vice versa, if $\mathcal {C}$ is a coalgebra with coproduct $\Delta _{\mathcal {C}}$ and counit ϵ, then we can naturally give $\mathcal {C}^*$ an algebra structure by defining the product via $\mu _{\mathcal {C}^*} = \Delta _{\mathcal {C}}^T$, i.e. by

$$\begin{aligned} (fg)(x) = (f\otimes g)\circ \Delta _{\mathcal {C}} (x), \end{aligned}$$

where $f,g\in \mathcal {C}^*$ and $x\in \mathcal {C}$; the unit of $\mathcal {C}^*$ is then ϵ.

Associativity of the coproduct allows us to write $\Delta ^2(x)$ instead of $(\Delta \otimes \textrm{Id}) \circ \Delta$, and $\Delta ^n(x)$ for *n* repeated application of Δ on *x*. In the following, we will use Sweedler’s notation of the coproduct and write

$$\begin{aligned} \Delta ^n(x) = \sum _{(x)} x_{(1)} \otimes x_{(2)} \otimes \dots \otimes x_{(n+1)}. \end{aligned}$$

We will show below that this shorthand notation actually hides a more complicated sum that has a special structure called matrix product state.

### Definition 3.2

(Matrix product states). Let $(V_i)_{i=1}^n$ and $(W_j)_{j=0}^n$ be two collections of finite dimensional vector spaces over $\mathbb {C}$. An MPS is given by tensors $A_i \in V_i \otimes W_{i-1} \otimes W_i^*$ (i=1,…,n), $A_i = \sum _k \vert k \rangle \otimes A_i^k$ and a matrix $X\in W_n \otimes W_0^*$; the state generated by the MPS is given by

**Figure:**
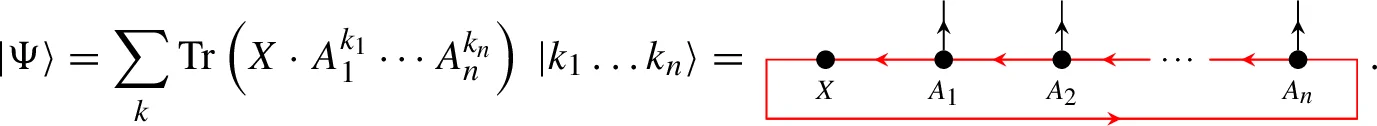

We say that the MPS is translation invariant with open boundary condition if $V_1 = \dots = V_n$, $W_0=\dots = W_n$ and $A_1 = \dots = A_n$.

Let us now show that in any coalgebra $\mathcal {C}$ the repeated coproduct of an element $x\in \mathcal {C}$, $\Delta ^{n-1}(x)$, can be represented as an MPS on *n* sites.

### Theorem 3.1

(MPS representation of coalgebras). Let $\mathcal {C}$ be a coalgebra, $V_i$ be finite dimensional vector spaces over $\mathbb {C}$ and $\phi _i: \mathcal {C}\rightarrow V_i$ linear maps (i=1,…,n). Let *W* be a vector space and $\psi : \mathcal {C}^* \rightarrow \textrm{End}(W)$ be an injective representation of the algebra $\mathcal {C}^*$. Let $A_i \in V_i \otimes \textrm{End}(W)$ be defined by

**Figure:**
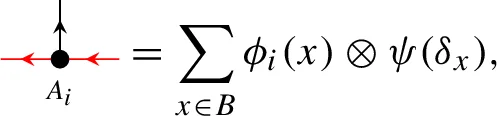

where *B* is a basis of $\mathcal {C}$, and $\delta _x$ denotes the dual basis elements (i.e. $\delta _x\in \mathcal {C}^*$ and $\delta _x(y) = \delta _{x,y}$ for any x,y∈B). Then for all $x\in \mathcal {C}$ there exists a matrix $b(x)\in \textrm{End}(W)$ such that for all n>0,

**Figure:**
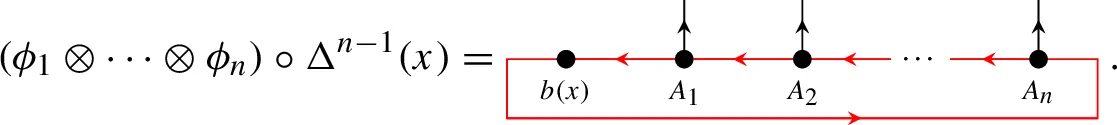

Let us remark that the tensor $A_i$ is independent of the concrete choice of the basis *B* of $\mathcal {C}$, as the expression $\sum _{x \in B} x\otimes \delta _x$ is independent of *B*. Let us also remark that might be many different choices for the matrix *b*(*x*) satisfying the required equation.

### Proof

Notice that

$$\begin{aligned} \sum _{x,y} f(x) \cdot g(y) \cdot \delta _x \delta _y = fg = \sum _{z\in B} fg(z) \cdot \delta _z = \sum _{z\in B} \sum _{(z)} f(z_{(1)}) \cdot g(z_{(2)}) \cdot \delta _z, \end{aligned}$$

where in both equations we have used that for any $f\in \mathcal {C}^*$, $f = \sum _{x\in B} f(x)\cdot \delta _x$. As this is true for all $f,g\in \mathcal {C}^*$, we conclude that

$$\begin{aligned} \sum _{x,y\in B} x\otimes y \otimes \delta _x \delta _y = \sum _{z\in B} \sum _{(z)} z_{(1)} \otimes z_{(2)} \otimes \delta _z = \sum _{z\in B} \Delta (z) \otimes \delta _z. \end{aligned}$$

This means that

**Figure:**
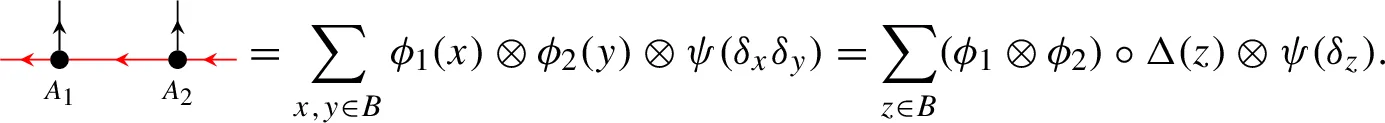

A similar equation holds for any number of consecutive tensors, i.e.

**Figure:**
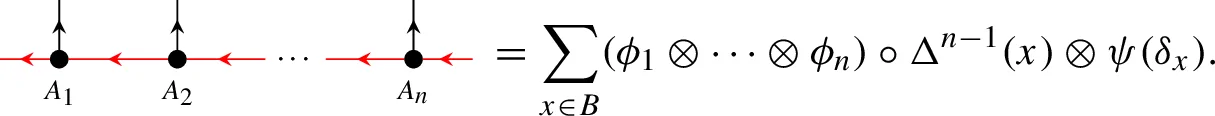

As ψ is an injective representation of $\mathcal {C}^*$, there exists a matrix *b*(*x*) for all $x\in \mathcal {C}$ such that $f(x) = \operatorname {Tr}\left( b(x) \cdot \psi (f)\right)$ holds for all $f\in \mathcal {C}^*$. Note that this equation does not uniquely determine *b*(*x*); there may be many different choices of *b*(*x*) satisfying this equation. Using any such choice of *b*(*x*), the following holds:

**Figure:**
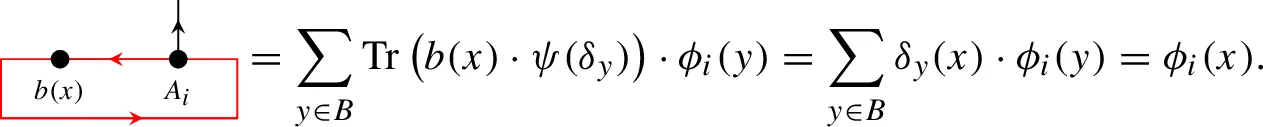

Finally, combining this statement with the previous one results in the desired equation

**Figure:**
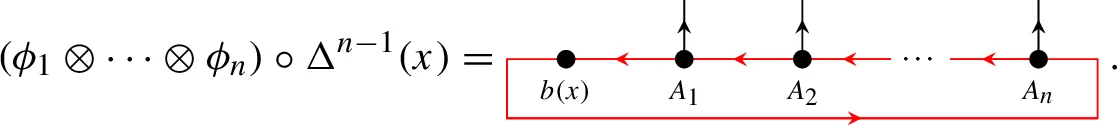

□

From now on, unless otherwise specified, we only consider translation invariant representations of coalgebras. This restriction is not essential and most statements obviously generalize to non-TI representations, but it eases the notation. For example, as all tensors are the same, we will drop the label *A* denoting the MPS tensor and simply write

**Figure:**
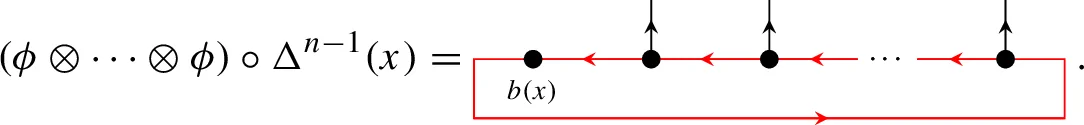

Let us now show that the construction of Theorem 3.1 can be reversed in the translation invariant case: given a translation invariant MPS with open boundary condition, one can construct a coalgebra $\mathcal {C}$ such that the MPS becomes a representing MPS of $\mathcal {C}$: Indeed, let $A\in V\otimes W \otimes W^*$ be an MPS tensor and let us write $A = \sum _i \vert i \rangle \otimes A^i$ (i=1,…,d with $d=\textrm{dim}(V)$) with $A^i \in \textrm{End}(W)$. Let us define the algebra $\mathcal {A}$ as

$$\begin{aligned} \mathcal {A} = \operatorname {Span} \bigcup _{n\in \mathbb {N}} \left\{ A^{i_1} A^{i_2} \cdots A^{i_n} \big | (i_1, \ldots , i_n) \in \{ 1,\ldots , d\}^{\times n} \right\} , \end{aligned}$$

and let $\mathcal {C} = \mathcal {A}^*$. Let us fix now a basis *B* of $\mathcal {C}$; elements of this basis are denoted by x,y,…. This basis then also fixes a basis (the dual basis) on $\mathcal {C}^* = \mathcal {A}$. Elements of this basis are denoted by $\delta _x,\delta _y,\ldots$. By definition the matrices $A^i$ are elements of $\mathcal {A}$, and thus one can expand them in this (dual) basis. One can thus write $A = \sum _{x\in B} \vert v_x \rangle \otimes \delta _x$ for some vectors $\vert v_x \rangle \in V$. This tensor then can be interpreted as a map $\phi :\mathcal {C}\rightarrow V$ by $\phi (x) = \sum _{y\in B} \delta _y(x) \vert v_y \rangle$. Note that—by the definition of the dual basis—for any x∈B, $\phi (x) = \vert v_x \rangle$. This implies that the following equation also holds: $A = \sum _{x\in B} \phi (x) \otimes \psi (\delta _x)$, with $\psi =\textrm{Id}$. Finally, as any element $x\in \mathcal {C}$ is a linear functional on $\mathcal {A}$ ($\mathcal {C} = \mathcal {A}^*$), one can find a matrix *b*(*x*) such that $x(m) = \operatorname {Tr}(b(x) m)$ for all matrices $m\in \mathcal {A}$. With this, we have obtained that for any $x\in \mathcal {C}$,

**Figure:**
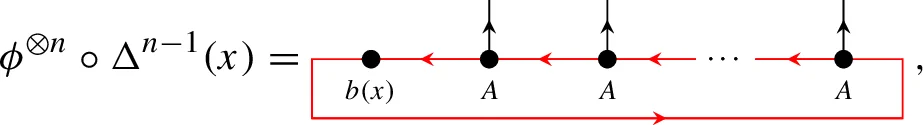

i.e. the MPS defined above forms a representation of $\mathcal {C}$ with the properties listed in Theorem 3.1.

### Cosemisimplicity and injectivity of the representing MPS

In this section we introduce cosemisimple coalgebras as well as injective MPS and examine the connection between these two properties: the MPS representation of a cosemisimple coalgebra decomposes into a sum of injective MPS, and conversely, given an MPS that decomposes into a direct sum of injective MPS, the corresponding coalgebra is cosemisimple.

Let $\mathcal {C}$ be a coalgebra (finite dimensional, over $\mathbb {C}$). As we have seen in the previous section, $\mathcal {C}^*$ has a natural algebra structure. In the following, we will talk about representations of this algebra $\mathcal {C}^*$, as the MPS construction in the previous section uses the representations of that algebra. Recall that two representations $\psi _1: \mathcal {C}^*\rightarrow \textrm{End}(W_1)$ and $\psi _2: \mathcal {C}^*\rightarrow \textrm{End}(W_2)$ are called equivalent if there is an invertible linear map $Z:W_1\rightarrow W_2$ such that $\psi _1(f) = Z^{-1} \cdot \psi _2(f) \cdot Z$ for all $f\in \mathcal {C}^*$. In particular, the dimension of the two equivalent representations coincide, $\textrm{dim}(W_1) = \textrm{dim}(W_2)$. The set of irreducible representation (irrep) equivalence classes of the algebra $\mathcal {C}^*$ is denoted by $\textrm{Irr}(\mathcal {C}^*)$, and the elements of this set (i.e. the different irrep equivalence classes) will be denoted by small Roman letters a,b,c,…. The dimension of (all) irreps from the class *a* will be denoted by $D_a$. For convenience, let us fix a concrete representation $\psi _{a}$ on vector space $W_a$ from each irrep class *a*. Recall that by the density theorem [EGH+], $\psi _a(\mathcal {C}^*) = \textrm{End}(W_a) \simeq \mathcal {M}_{D_a}$ for all irreps $\psi _a$, where $\mathcal {M}_{D_a}$ denotes the set of $D_a\times D_a$ matrices over $\mathbb {C}$; in fact $\phi (\mathcal {C}^*) = \bigoplus _{a\in I \subseteq \textrm{Irr}(\mathcal {C}^*)} \mathcal {M}_{D_a} \otimes \textrm{Id}_{m_a}$ for all representations of the form $\phi (x) = \bigoplus _{a\in I \subseteq \textrm{Irr}(\mathcal {C}^*)} \phi _a(x)\otimes \textrm{Id}_{m_a}$.

We say that the coalgebra $\mathcal {C}$ is *cosemisimple* if the algebra $\mathcal {C}^*$ is semisimple, i.e. if $\mathcal {C}^*\simeq \bigoplus _{a\in \textrm{Irr}(\mathcal {C}^*)} \mathcal {M}_{D_a}$. In particular, $\mathcal {C}^*$ has finitely many irrep classes. If $\mathcal {C}^*$ is semisimple, then any representation ψ of it, up to a basis transformation, is of the form

$$\begin{aligned} \psi (x) \simeq \bigoplus _{a\in I \subseteq \textrm{Irr}(\mathcal {C}^*)} \psi _a (x) \otimes \textrm{Id}_{m_a}, \end{aligned}$$

where *a* runs over a subset *I* of the equivalence classes of irreps of $\mathcal {C}^*$ and $\psi _a$ are the previously fixed representatives from the class *a*, and the numbers $m_a$ denote the multiplicity of the irrep $\psi _a$ in the decomposition of ψ. The representation ψ is injective if and only if all irrep classes are present in this decomposition,^1^ i.e. if $I=\textrm{Irr}(\mathcal {C}^*)$. As ψ is a direct sum of irreps, the density theorem applies and thus $\psi (\mathcal {C}^*) = \bigoplus _{a\in I \subseteq \textrm{Irr}(\mathcal {C}^*)} \mathcal {M}_{D_a} \otimes \textrm{Id}_{m_a}$.

The MPS tensor *A* in Theorem 3.1 is constructed using a representation ψ of $\mathcal {C}^*$ that is assumed to be injective. Therefore, if $\mathcal {C}$ is cosemisimple, *A* decomposes as

$$\begin{aligned} A \simeq \sum _{x\in B} \phi (x) \otimes \bigoplus _{a\in \textrm{Irr}(\mathcal {C}^*)} \psi _a (\delta _x) \otimes \textrm{Id}_{m_a}. \end{aligned}$$

As the defining property of *b*(*x*) is that $\operatorname {Tr}\left( b(x) \psi (f)\right) = f(x)$, the matrix *b*(*x*) can also be chosen w.l.o.g. in this form, i.e. such that in the same basis as ψ, it reads

$$\begin{aligned} b(x) \simeq \bigoplus _{a\in \textrm{Irr}(\mathcal {C}^*)} b_a (x) \otimes \textrm{Id}_{m_a}. \end{aligned}$$

If *b*(*x*) is in this form, then it is uniquely defined by the equation $\operatorname {Tr}\left( b(x) \psi (f)\right) = f(x)$ and the map x↦b(x) is linear and a bijection between $\mathcal {C}$ and $\bigoplus _{a\in \textrm{Irr}(\mathcal {C}^*)} \mathcal {M}_{D_a}$.

We have thus obtained that the MPS representing a cosemisimple coalgebra decomposes into a sum of MPSs with smaller bond dimension,

**Figure:**
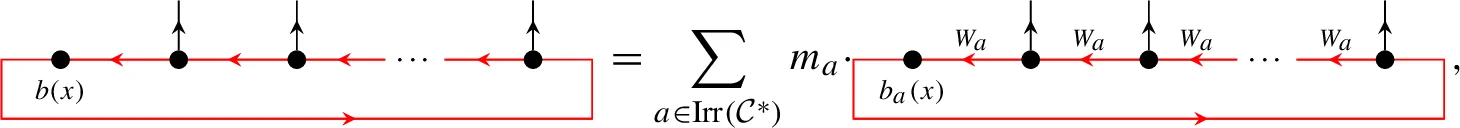

where

**Figure:**
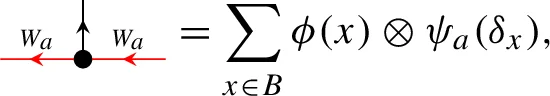

for the previously fixed irrep representatives $\psi _a$ in the irrep class *a*. In the following, we will choose the representation ψ such that it contains exactly one irrep from each irrep class (i.e. such that $m_a=1$). The previously fixed irrep representatives $\psi _a$ determine the vector spaces $W_a$ for each *a*, and thus from now on, we do not display the labels $W_a$ in graphical representation, only the label *a*. Therefore we will write

**Figure:**
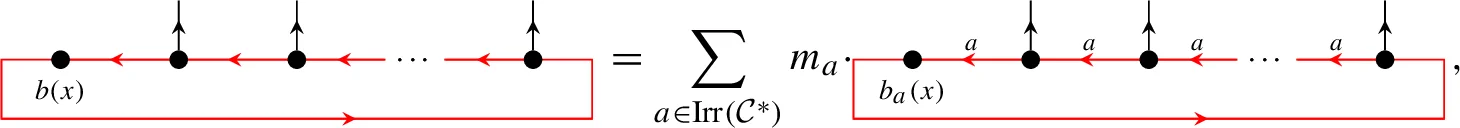

with

**Figure:**
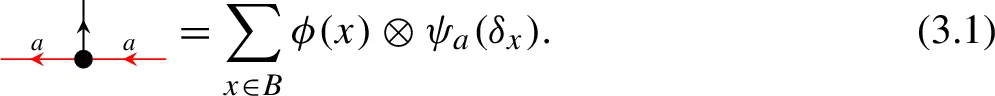

The MPS tensors in Eq. (3.1), provided that ϕ has certain properties, are special:

#### Definition 3.3

(Injective and normal MPS tensor). An MPS tensor $A\in V\otimes \textrm{End}(W)$, $A = \sum _i \vert i \rangle \otimes A^i$ is *normal* if there is an $n\in \mathbb {N}$ such that

$$\begin{aligned} \operatorname {Span} \left\{ A^{i_1} \cdots A^{i_n} \big | (i_1, \ldots , i_n) \in \{1, \ldots , \textrm{dim}(V) \}^{\times n} \right\} = \textrm{End}(W). \end{aligned}$$

The tensor is called *injective* if it is normal with n=1.

It is immediate to see that the MPS tensors defined in Eq. (3.1) are injective if and only if ϕ is injective and they are normal if and only if there is an *n* such that $\phi ^{\otimes n} \circ \Delta ^{n-1}$ is injective. We will call such a linear map *normal*.

We have thus obtained that if $\mathcal {C}$ is a cosemisimple coalgebra and ϕ is a $\mathcal {C}\rightarrow V$ linear map such that $\phi ^{\otimes n} \circ \Delta ^{n-1}$ is injective for some *n*, then the coproduct of an element *x* has a special MPS representation of the form

**Figure:**
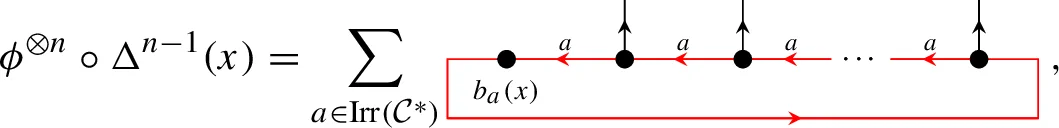

where each MPS tensor is normal. This statement now can be reversed: let us consider a set $\mathcal {S}$ of injective MPS tensors $A\in V \otimes \textrm{End}(W_A)$ for each $A\in \mathcal {S}$ such that no two of them are related to each other with a basis transformation. One then can construct a *cosemisimple* coalgebra $\mathcal {C}$ (using the construction from the previous section), a map $\phi : \mathcal {C}\rightarrow V$ and a bijection $B: \bigoplus \textrm{End}(W_A) \rightarrow \mathcal {C}$, the inverse of the map x↦b(x), such that

**Figure:**
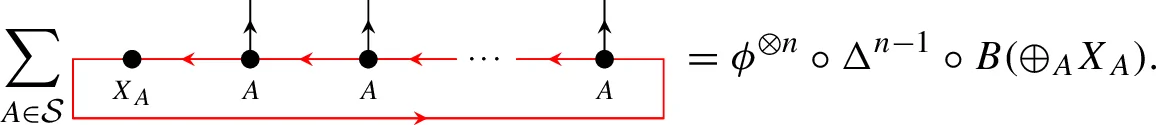

### Cocentral and non-degenerate elements

In this section we define the co-center of a coalgebra and show that in a cosemisimple coalgebra the set of cocentral elements have special MPS representations—they are translation invariant MPS with periodic boundary condition.

Let $\mathcal {C}$ be a coalgebra with coproduct Δ. Then the map $\Delta _{\textrm{op}}:\mathcal {C}\rightarrow \mathcal {C}\otimes \mathcal {C}$, $x\mapsto \Delta _{\textrm{op}}(x)$ defined by swapping the two components of the tensor product in Δ(x),

$$\begin{aligned} \Delta _{\textrm{op}}(x) = \sum _{(x)} x_{(2)} \otimes x_{(1)}, \end{aligned}$$

is also a coproduct. Using the opposite coproduct, we can define cocentral elements as:

#### Definition 3.4

An element $x\in \mathcal {C}$ is called *cocentral* or *trace-like* if it satisfies $\Delta _{\textrm{op}}(x) = \Delta (x)$.

Due to the definition of the product in $\mathcal {C}^*$, if $x\in \mathcal {C}$ is cocentral, then for all $f,g\in \mathcal {C}^*$,

$$\begin{aligned} (fg)(x) = (f\otimes g)\circ \Delta (x) = (g\otimes f)\circ \Delta _{\textrm{op}}(x) = (g\otimes f)\circ \Delta (x) = (gf)(x). \end{aligned}$$

This means thus that x:f↦f(x) is a trace-like (cyclic) linear functional on $\mathcal {C}^*$, i.e. the set of cocentral elements of $\mathcal {C}$ is exactly the set of trace-like linear functionals of $\mathcal {C}^*$. Due to their cyclicity, repeated coproducts of these elements are translation invariant:

$$\begin{aligned} \sum _{(x)} x_{(n)} \otimes x_{(1)} \otimes \dots \otimes x_{(n-1)}&\,{=}\, (\textrm{Id}\otimes \Delta ^{n-2}) \circ \Delta _{\textrm{op}} (x) {=} (\textrm{Id}\otimes \Delta ^{n-2}) \circ \Delta (x)\\&{=} \Delta ^{n-1} (x) = \sum _{(x)} x_{(1)} \otimes x_{(2)} \otimes \dots \otimes x_{(n)}. \end{aligned}$$

The corresponding MPS representation is also translation invariant, i.e. *b*(*x*) is such that

**Figure:**
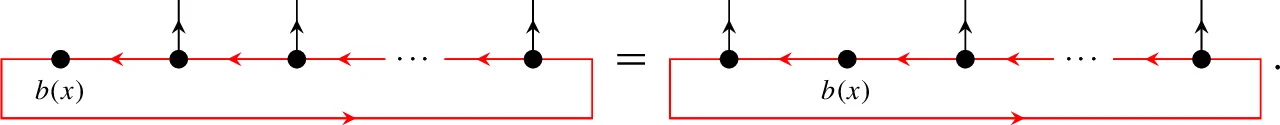

Let us assume now that $\mathcal {C}$ is cosemisimple. In this case, cocentral elements of $\mathcal {C}$ have very simple MPS representations: if $x\in \mathcal {C}$ is cocentral and ϕ is normal, then

**Figure:**
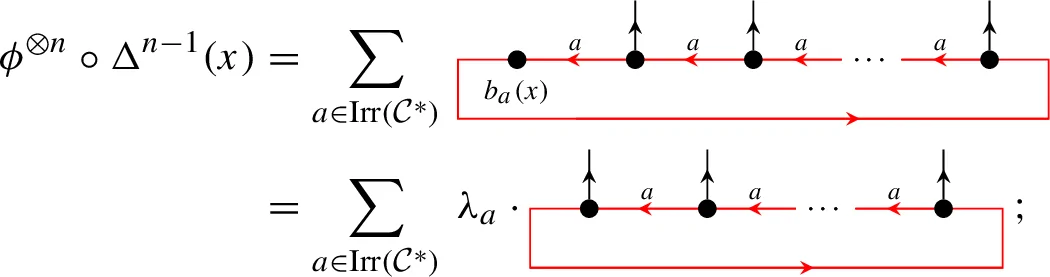

indeed, the boundary matrix $b(x) = \oplus _a b_a(x)$ is defined by $\operatorname {Tr}(b(x) \psi (f)) = f(x)$ for all *x*, and as *x* is trace-like, $\operatorname {Tr}(b_a(x) \psi _a(f)\psi _a(g)) = \operatorname {Tr}(\psi _a(f) b_a(x) \psi _a(g))$ for all $f,g\in \mathcal {C}^*$ and $a\in \textrm{Irr}(\mathcal {C}^*)$. As $\psi _a$ is an irrep, both $\psi _a(f)$ and $\psi _a(g)$ span the full matrix algebra and thus $b_a(x)$ is necessarily proportional to the identity.

Let $\tau _a$ denote the character of the irrep class *a*. As $\tau _a$ is a linear functional on $\mathcal {C}^*$, it can also be viewed as an element of the coalgebra $\mathcal {C}$. By definition, $\tau _a(f) = \operatorname {Tr}(\psi _a(f))$, and thus it can be written as $\tau _a(x) = \operatorname {Tr}(b(\tau _a) \psi (f))$ with $b_b(\tau _a) = \delta _{ab} \cdot \textrm{Id}_a$. That is, the MPS representation of $\tau _a$ is

**Figure:**
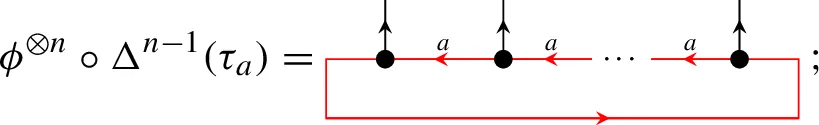

Another set of special elements in the coalgebra are those that have full-rank coproduct:

#### Definition 3.5

An element $x\in \mathcal {C}$ is called *non-degenerate* if its coproduct Δ(x) is full rank.

Equivalently, *x* is non-degenerate if and only if

$$\begin{aligned} (\textrm{Id}\otimes \mathcal {C}^*)\circ \Delta (x) = (\mathcal {C}^*\otimes \textrm{Id}) \circ \Delta (x) = \mathcal {C}, \end{aligned}$$

or, with other words, if for all $y\in \mathcal {A}$ there exist linear functionals *f* and *g* such that

$$\begin{aligned} y = (\textrm{Id}\otimes f)\circ \Delta (x) = (g\otimes \textrm{Id}) \circ \Delta (x). \end{aligned}$$

Let us now show that if $\mathcal {C}$ is cosemisimple, then *x* is non-degenerate if and only if its MPS representation is of the form

**Figure:**
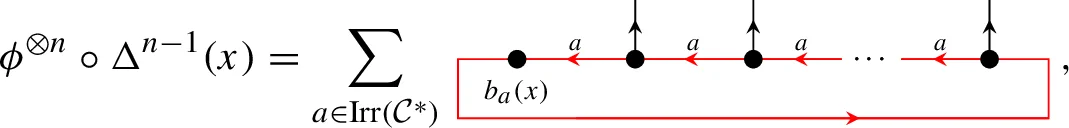

where all $b_a(x)$ are invertible. To prove this, note first that for any linear functional $f\in \mathcal {C}^*$ the element $y = (f \otimes \textrm{Id})\circ \Delta (x)$ is described by the MPS

**Figure:**
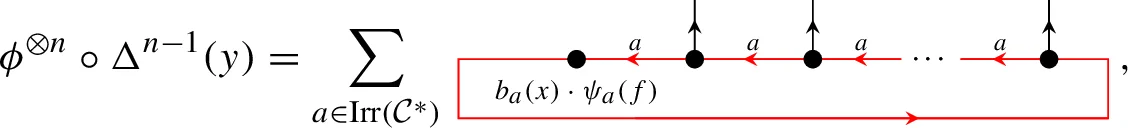

i.e. the boundary describing $y=(f \otimes \textrm{Id})\circ \Delta (x)$ is given by $b_a(y) = b_a(x) \cdot \psi _a(f)$ in every sector $a\in \textrm{Irr}(\mathcal {C}^*)$. Similarly, the boundary describing $y = (\textrm{Id}\otimes g)\circ \Delta (x)$ is given by $b_a(y) = \psi _a(g) \cdot b_a(x)$. Therefore *x* is non-degenerate if and only if for all $y\in \mathcal {C}$ there are $f,g\in \mathcal {C}^*$ such that

$$\begin{aligned} b_a(y) = \psi _a(g) \cdot b_a(x) = b_a(x) \cdot \psi _a(f). \end{aligned}$$

As $\psi _a(f)$ can be any matrix, this is equivalent with the invertibility of $b_a(x)$.

As a particular case of the previous statement, let us consider a cocentral coalgebra element $x = \sum _a \lambda _a \tau _a$, where $\tau _a\in \mathcal {C}$ are the irrep characters of $\mathcal {C}^*$. Then, *x* is non-degenerate if and only if $\lambda _a \ne 0$ for all $a\in \textrm{Irr}(\mathcal {C}^*)$. For example, the cocentral element $\Theta = \sum _a \tau _a$ with MPS representation

**Figure:**
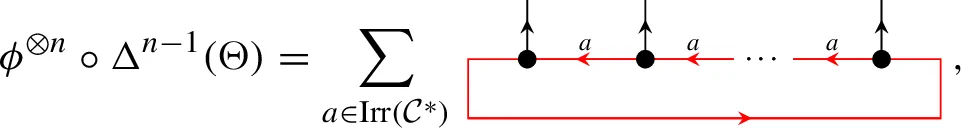

is a non-degenerate cocentral element. Using this element Θ, one can interpret *b*(*x*) (more precisely, $\psi ^{-1}(b(x))$) as the linear functional that satisfies $(\psi ^{-1}(b(x))\otimes \textrm{Id})\circ \Delta (\Theta ) = x$.

## Pre-bialgebras and their Matrix Product Operator Representations

In this section we define pre-bialgebras (bialgebras without any property imposed on the unit and counit) as well as matrix product operators. We show that the MPS representations of coalgebras from the previous section naturally generalize to pre-bialgebras providing an MPO representation for them. We further investigate this MPO representation for cosemisimple pre-bialgebras.

### Definition 4.1

(Pre-bialgebra). $\mathcal {A}$ is a *pre-bialgebra* if it is both an algebra and a coalgebra such that the coproduct Δ is multiplicative, i.e. for all $x,y\in \mathcal {A}$

$$\begin{aligned} \Delta (xy) = \Delta (x) \Delta (y). \end{aligned}$$

In this equation the multiplication in $\mathcal {A}\otimes \mathcal {A}$ is taken component-wise, i.e. $(x\otimes y ) \cdot (z\otimes w) = xz \otimes yw$. If $\mathcal {A}$ is a pre-bialgebra with product $\mu _{\mathcal {A}}$ and coproduct $\Delta _{\mathcal {A}}$, then $\mathcal {A}^*$ is also a pre-bialgebra with product $\mu _{\mathcal {A}^*} = \Delta _{\mathcal {A}}^T$ and coproduct $\Delta _{\mathcal {A}^*} = \mu _{\mathcal {A}}^T$. It is clear from context whether we refer to the coproduct of $\mathcal {A}$ or that of $\mathcal {A}^*$, and thus in the following we drop the subscript $\mathcal {A}$ and $\mathcal {A}^*$, and simply write Δ for both $\Delta _{\mathcal {A}}$ and $\Delta _{\mathcal {A}^*}$. That is, the product and coproduct in $\mathcal {A}^*$ are such that for all $f,g\in \mathcal {A}^*$ and $x,y\in \mathcal {A}$,

$$\begin{aligned} (fg)(x) = (f\otimes g) \circ \Delta (x) \quad \text {and} \quad \left[ \Delta (f)\right] (x\otimes y) = f(xy). \end{aligned}$$

The unit of $\mathcal {A}^*$ is the counit of $\mathcal {A}$ and the counit of $\mathcal {A}^*$ is the unit of $\mathcal {A}$.

In the following, we will talk about representations of the pre-bialgebra $\mathcal {A}$. These representations should be understood as representations of the algebraic structure of $\mathcal {A}$ (i.e. disregarding the coalgebra structure). The extra structure given by the coproduct allows us to define the tensor product of representations. Let $\phi _1:\mathcal {A}\rightarrow \textrm{End}(V_1)$ and $\phi _2:\mathcal {A}\rightarrow \textrm{End}(V_2)$ be two representations of $\mathcal {A}$. Then, as the coproduct Δ is multiplicative, $(\phi _1\otimes \phi _2) \circ \Delta : \mathcal {A} \rightarrow \textrm{End}(V_1\otimes V_2)$ is also multiplicative. This map is not a representation on $V_1\otimes V_2$, however, since $(\phi _1\otimes \phi _2) \circ \Delta (1)$ is not the identity, unless $\Delta (1)= 1\otimes 1$. The element $(\phi _1\otimes \phi _2) \circ \Delta (1)$ is a projector and is absorbed by any element from $(\phi _1\otimes \phi _2) \circ \Delta (\mathcal {A})$, as a1=1a=a for all $a\in \mathcal {A}$. This means that all operators in $(\phi _1\otimes \phi _2) \circ \Delta (\mathcal {A})$ can be restricted to the range of $(\phi _1\otimes \phi _2) \circ \Delta (1)$. Let $V_1\boxtimes V_2$ denote this subspace of $V_1\otimes V_2$. By definition, $(\phi _1\otimes \phi _2) \circ \Delta (1)$ restricted to $V_1\boxtimes V_2$, $(\phi _1\otimes \phi _2) \circ \Delta (1)|_{V_1\boxtimes V_2}$, is the identity, and thus the map defined by $(\phi _1 \boxtimes \phi _2)(x) = (\phi _1\otimes \phi _2) \circ \Delta (x)|_{V_1\boxtimes V_2}$ is a representation of $\mathcal {A}$. This representation is then called the tensor product of the representations $\phi _1$ and $\phi _2$. Using associativity of the coproduct, one can define the *n*-fold tensor product of representations for any n>2 integer as well: given $\phi _i:\mathcal {A}\rightarrow \textrm{End}(V_i)$ representation for i=1⋯n, the tensor product representation $\phi _1 \boxtimes \dots \boxtimes \phi _n$ is given by the restriction of $(\phi _1\otimes \dots \otimes \phi _n)\circ \Delta ^{n-1}(x)$ onto the range of $(\phi _1\otimes \dots \otimes \phi _n)\circ \Delta ^{n-1}(1)$ in the vector space $V_1 \otimes \dots \otimes V_n$.

Just as in the previous section, using the coalgebra structure of $\mathcal {A}$, one can form MPS representations of $\mathcal {A}$. As $\mathcal {A}$ has an algebra structure as well, it is natural to choose the linear map ϕ used at the construction of the MPS to be a representation of the algebra. The resulting MPS is then interpreted as an operator, and in fact, this structure is called a matrix product operator (MPO):

### Definition 4.2

(Matrix product operators). Let $(V_i)_{i=1}^n$ and $(W_j)_{j=0}^n$ be two collections of finite dimensional vector spaces over $\mathbb {C}$. An MPO is given by tensors $A_i \in V_i \otimes V_i^* \otimes W_{i-1} \otimes W_i^*$ (i=1,…,n), $A_i = \sum _{kl} \vert k \rangle \langle l \vert \otimes A_i^{kl}$ and a matrix $X\in W_n \otimes W_0^*$; the operator generated by the MPO is given by

**Figure:**
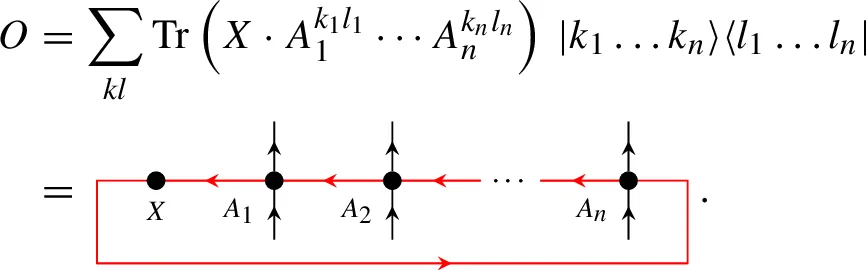

Fixing a representation $\phi : \mathcal {A} \rightarrow \textrm{End}(V)$ of $\mathcal {A}$ and an injective representation $\psi :\mathcal {A}^*\rightarrow \textrm{End}(W)$ of $\mathcal {A}^*$, one can repeat the procedure described in the previous section to form MPOs representing the coalgebra structure of $\mathcal {A}$:

**Figure:**
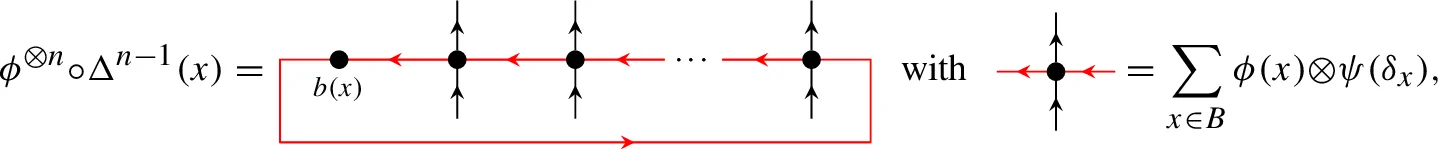

where *B* is a basis of $\mathcal {A}$, and ϕ is a representation of $\mathcal {A}$, and the matrices *b*(*x*) are such that $\operatorname {Tr}\left( b(x) \psi (f) \right) = f(x)$. As described above, the map $\phi ^{\otimes n}\circ \Delta ^{n-1}$, in general, is not a representation of $\mathcal {A}$ as 1 is not mapped to the identity operator on the space $V^{\otimes n}$:

**Figure:**
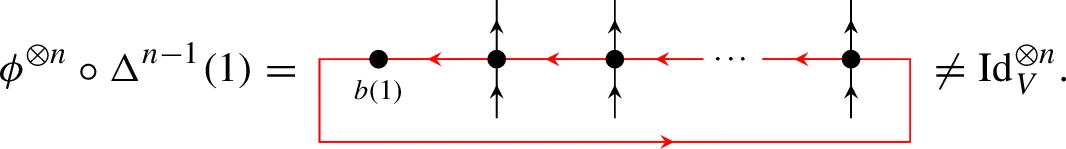

These MPOs, nevertheless, are multiplicative as $\phi ^{\otimes n}\circ \Delta ^{n-1}$ is multiplicative:

**Figure:**
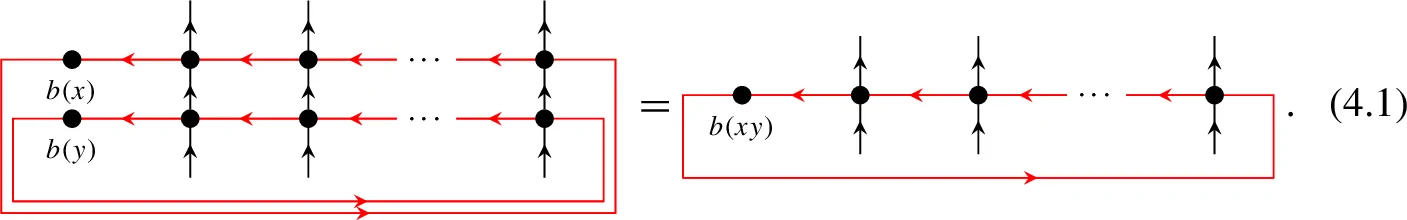

In particular, these MPOs are invariant under multiplying with $\phi ^{\otimes n} \circ \Delta ^{n-1}(1)$ from either side,

**Figure:**
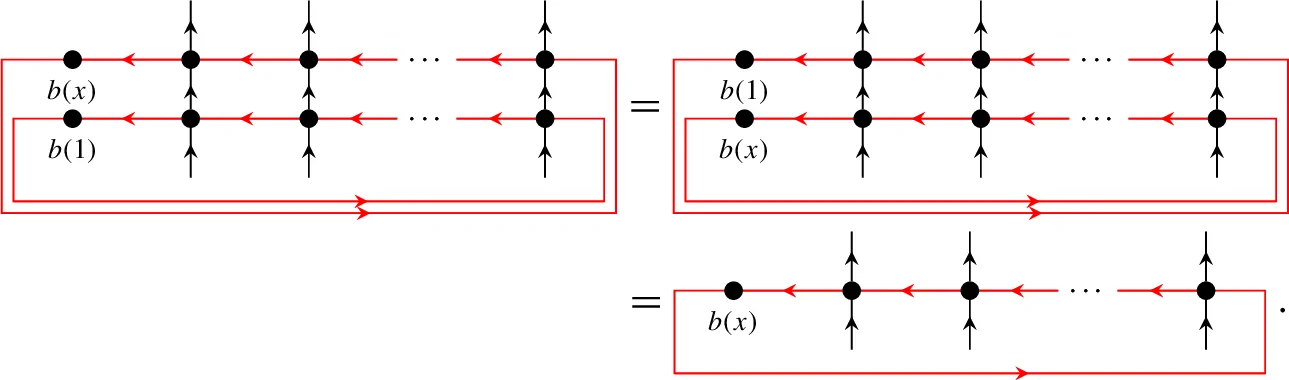

One can thus restrict $V^{\otimes n}$ to the range of $\phi ^{\otimes n} \circ \Delta ^{n-1}(1)$ (notice that this is a projector as 1·1=1), and on this space the MPOs form a representation of $\mathcal {A}$. Note that this restriction is only necessary for n>1; for n=1 the representing MPOs are simply $x\mapsto \phi (x)$:

**Figure:**
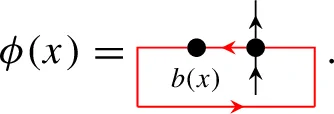

In particular, on a single site, unlike for n>1 sites, the unit is represented by the identity operator:

**Figure:**
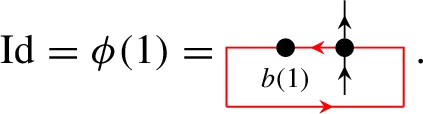

### Cosemisimplicity

Let $\mathcal {A}$ be a pre-bialgebra, then $\mathcal {A}^*$ is a pre-bialgebra as well. As such, one can form tensor products of its representations, i.e. if $\psi _1:\mathcal {A}^*\rightarrow \textrm{End}(W_1)$ and $\psi _2:\mathcal {A}^*\rightarrow \textrm{End}(W_2)$ are representations of $\mathcal {A}^*$, then $(\psi _1\otimes \psi _2)\circ \Delta _{\mathcal {A}^*}$ restricted to the range of $(\psi _1\otimes \psi _2)\circ \Delta _{\mathcal {A}^*}(\epsilon )$ is a representation^2^ of $\mathcal {A}^*$ denoted by $\psi _1\boxtimes \psi _2$.

Just as for coalgebras, we say that a pre-bialgebra $\mathcal {A}$ is cosemisimple if the algebra $\mathcal {A}^*$ is semisimple, i.e. if every representation of it decomposes into a direct sum of irreps. In particular, let us fix irreps $\psi _a:\mathcal {A}^*\rightarrow \textrm{End}(W_a)$ for each irrep class $a\in \textrm{Irr}(\mathcal {A}^*)$; then given two of these irreps, $\psi _a$ and $\psi _b$, their tensor product $\psi _a\boxtimes \psi _b$ decomposes into a direct sum of irreps as follows:

$$\begin{aligned} \psi _a\boxtimes \psi _b \simeq \bigoplus _{c\in \textrm{Irr}(\mathcal {A}^*)} \psi _c \otimes \textrm{Id}_{N_{ab}^c}, \end{aligned}$$

i.e. in the decomposition of $\psi _a\boxtimes \psi _b$ the irrep $\psi _c$ appears $N_{ab}^c$ times. These $N_{ab}^c$ non-negative integers are called *fusion multiplicities*. Note that $N_{ab}^c$ might be 0; in that case the irrep $\psi _c$ does not appear in the decomposition. The above equation holds up to a basis transformation, i.e. for all $a,b,c\in \textrm{Irr}(\mathcal {A}^*)$ there are invertible operators $Z_{ab}: \bigoplus _c W_c \otimes \mathbb {C}^{N_{ab}^c} \rightarrow W_a \boxtimes W_b$ such that for all $f\in \mathcal {A}^*$

4.2 $$\begin{aligned} \left( \psi _a\boxtimes \psi _b\right) (f) = Z_{ab} \left( \bigoplus _{c\in \textrm{Irr}(\mathcal {A}^*)} \psi _c(f) \otimes \textrm{Id}_{N_{ab}^c}\right) \left( Z_{ab}\right) ^{-1}, \end{aligned}$$

or equivalently, for all $a,b,c\in \textrm{Irr}(\mathcal {A}^*)$ there are $N_{ab}^c$ operators $Z_{ab}^{c\mu }: V_c \rightarrow V_{a}\boxtimes V_b$ and $Y_{ab}^{c\mu }: V_a\boxtimes V_b\rightarrow V_c$ (here $\mu =1\dots N_{ab}^c$ are integers) such that

$$\begin{aligned} \left( \psi _a\boxtimes \psi _b\right) (f) = \sum _{c\in \textrm{Irr}(\mathcal {A}^*)} \sum _{\mu =1}^{N_{ab}^c} Z_{ab}^{c\mu } \cdot \psi _c(f) \cdot Y_{ab}^{c\mu } \quad \text {and} \quad Y_{ab}^{c\mu } \cdot Z_{ab}^{d\nu } = \delta _{cd}\cdot \delta _{\mu \nu } \cdot \textrm{Id}_{V_c}. \end{aligned}$$

These operators then can be extended^3^ to map from (and to) $W_a \otimes W_b$ instead of $W_{a}\boxtimes W_b$, i.e. there are linear maps $V_{ab}^{c\mu }: W_c \rightarrow W_{a}\otimes W_b$ and $W_{ab}^{c\mu }: W_a \otimes W_b\rightarrow W_c$, called *fusion tensors*, such that

4.3 $$\begin{aligned} (\psi _a \otimes \psi _b)\circ \Delta (f) = \sum _{c} \sum _{\mu =1}^{N_{ab}^c} V_{ab}^{c\mu } \cdot \psi _c(f) \cdot W_{ab}^{c\mu } \quad \text {and} \quad W_{ab}^{c\mu } \cdot V_{ab}^{d\nu } = \delta _{cd} \cdot \delta _{\mu \nu } \cdot \textrm{Id}_{W_c}, \end{aligned}$$

for all $a,b,c\in \textrm{Irr}(\mathcal {A}^*)$ and $f\in \mathcal {A}^*$. The fusion tensors are rank-four tensors; the index μ, however, plays a very different role than the rest of its indices. This allows us to think of the fusion tensors as a set for rank-three tensors instead, and write

**Figure:**
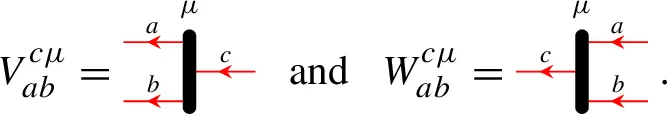

Using this notation, the graphical representation of Eq. (4.3) is the following:

**Figure:**
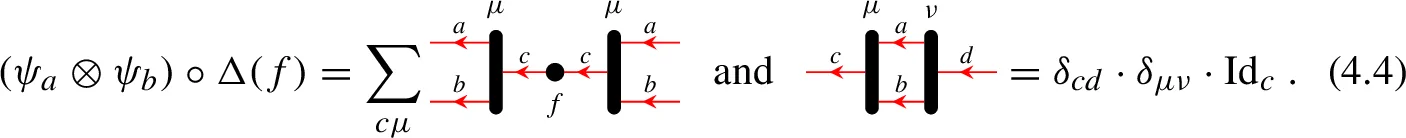

Let us stress again that the fusion tensors $V_{ab}^{c\mu }$ (and $W_{ab}^{c\mu }$) do not map to (and from) the whole space $W_a\otimes W_b$, instead only to (and from) the subspace $W_a\boxtimes W_b$. A projector onto this subspace is $(\psi _a\otimes \psi _{b})\circ \Delta (\epsilon ) \ne \textrm{Id}_a\otimes \textrm{Id}_b$, that has the graphical representation

**Figure:**
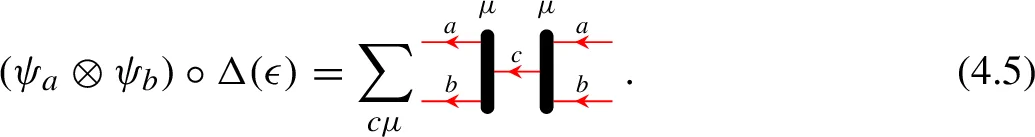

The operators $V_{ab}^{c\mu }$ and $W_{ab}^{c\mu }$ are not unique, instead only their tensor product $\sum _\mu V_{ab}^{c\mu } \otimes W_{ab}^{c\mu }$ is fixed. This allows for a basis change: $\sum _\mu V_{ab}^{c\mu } \otimes W_{ab}^{c\mu } = \sum _{\mu } \hat{V}_{ab}^{c\mu } \otimes \hat{W}_{ab}^{c\mu }$ if and only if $\hat{V}_{ab}^{c\mu } = \sum _\nu K_{\mu \nu } V_{ab}^{c\nu }$ and $\hat{W}_{ab}^{c\mu } = \sum _\nu (K^{-1})_{\nu \mu } W_{ab}^{c\nu }$ for some $N_{ab}^c \times N_{ab}^c$ complex invertible matrix *K*. Note as well that the value of $\sum _\mu V_{ab}^{c\mu } \otimes W_{ab}^{c\mu }$ depends on the choices of the irrep representatives $\psi _a$ that we have made at the beginning of this section; different choices will lead to a gauge transformation of $V_{ab}^{c\mu }$ and $W_{ab}^{c\mu }$, i.e. if the irrep representatives are chosen to be $\hat{\psi }_a = U_a \psi _a U_a^{-1}$ instead of $\psi _a$ for each $a\in \textrm{Irr}(\mathcal {A}^*)$ (here $U_a$ are general invertible matrices), then the corresponding $\hat{V}_{ab}^{c\mu }$ and $\hat{W}_{ab}^{c\mu }$ are given by

$$\begin{aligned} \hat{V}_{ab}^{c\mu } = (U_a \otimes U_b) \cdot V_{ab}^{c\mu } \cdot U_c^{-1} \quad \text {and} \quad \hat{W}_{ab}^{c\mu } = U_c \cdot W_{ab}^{c\mu } \cdot (U_a^{-1} \otimes U_b^{-1}). \end{aligned}$$

Let us now show using this graphical language how the so-called *F*-symbols of the representation category of $\mathcal {A}^*$ emerge. Using Eq. (4.4), associativity of the coproduct of $\mathcal {A}^*$ implies that for all $f\in \mathcal {A}^*$ and $a,b,c,e\in \textrm{Irr}(\mathcal {A}^*)$,

**Figure:**
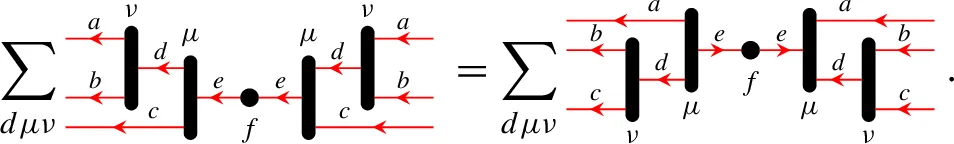

As this equation holds for all $f\in \mathcal {A}^*$, and $\psi _e(\mathcal {A}^*)=\mathcal {M}_{D_e}$ for each irrep block *e*, i.e. the matrix $\psi _e(f)$ can take any value, we conclude that

**Figure:**
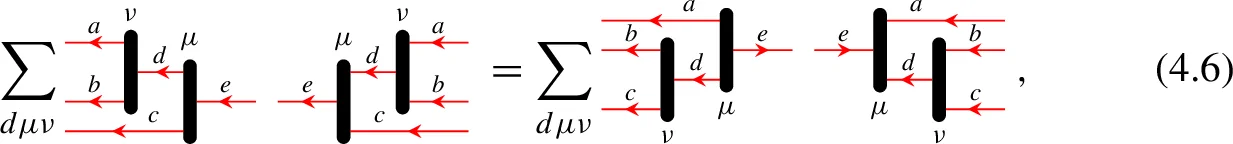

or equivalently, that there is an invertible (square) matrix $F^{abc}_e$ of dimension $\sum _{d} N_{ab}^d N_{dc}^e {=} \sum _d N_{bc}^d N_{ad}^e$ such that

**Figure:**
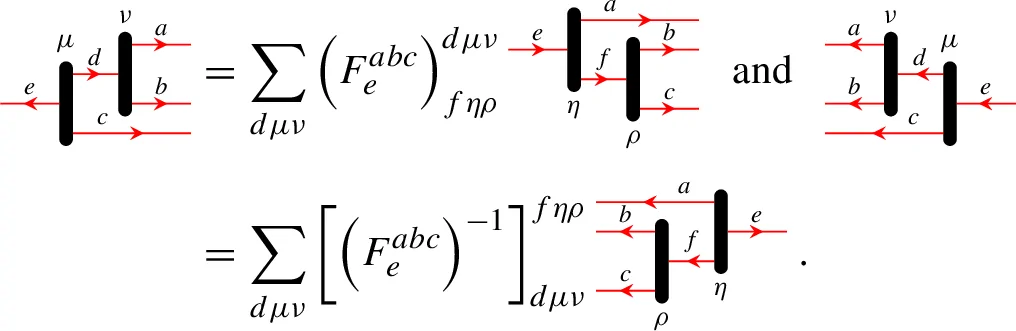

Standard arguments show that these *F*-symbols satisfy a consistency condition called the pentagon equation.

Let us now investigate the MPO representations of a cosemisimple pre-bialgebra $\mathcal {A}$. Just as in the MPS case, the MPOs representing $\mathcal {A}$ decompose into a sum of MPOs with smaller bond dimension corresponding to the irreps of $\mathcal {A}^*$. We denote these MPOs by writing the label $a\in \textrm{Irr}(\mathcal {A}^*)$ on the virtual indices of the tensor:

**Figure:**
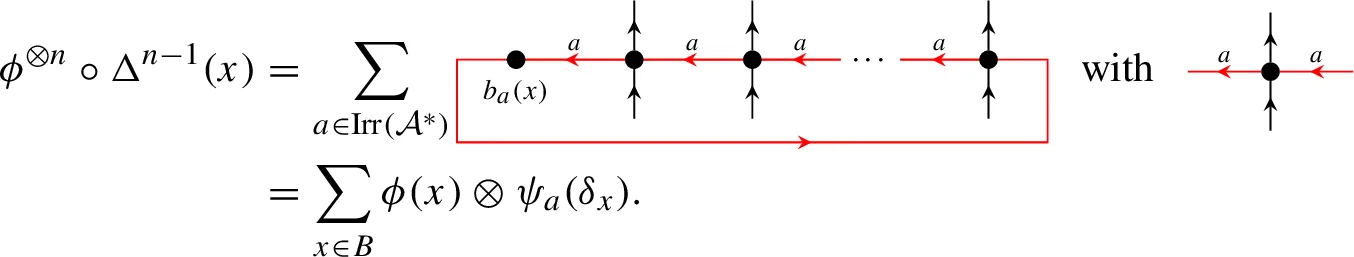

If ϕ is injective, then each smaller MPO tensor is injective and if there is a positive integer *n* such that $\phi ^{\otimes n} \circ \Delta ^{n-1}$ is injective, then they are normal. The above decomposition is thus nothing but the decomposition of the MPO into its normal components.

Let us now consider the product of two MPOs each representing an element of $\mathcal {A}$, such as in Eq. (4.1). The l.h.s. of this equation then decomposes to injective blocks as described above. On the r.h.s. both MPOs can be decomposed into injective blocks, therefore their product can also be decomposed into a sum:

**Figure:**
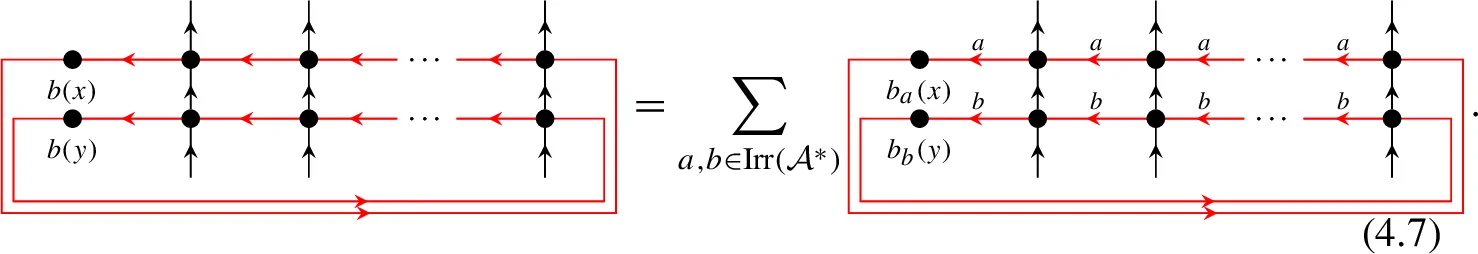

It turns out, however, that these MPOs are still not injective, i.e. they can be further decomposed. To understand why, let us investigate the tensor describing such an MPO:

**Figure:**
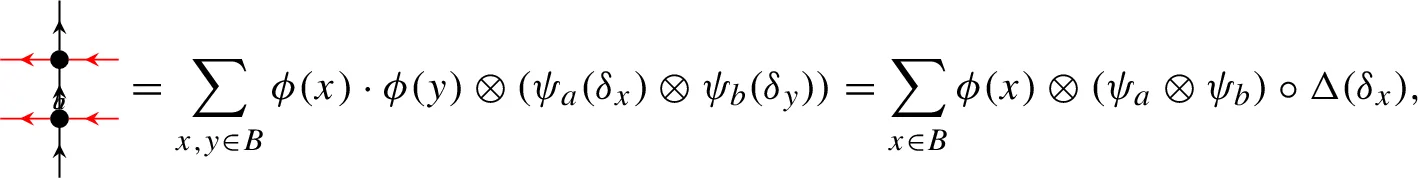

where the last equality, analogous to the proof of Theorem 3.1, holds because for every $f\in \mathcal {A}^*$,

$$\begin{aligned} \sum _{x,y\in B} f(xy) \cdot \delta _x \otimes \delta _y = \sum _{x,y\in B, (f)} f_{(1)}(x) \cdot f_{(2)}(y) \cdot \delta _x\otimes \delta _y = \sum _{(f)} f_{(1)} \otimes f_{(2)} = \sum _{x\in B} f(x) \cdot \Delta (\delta _x), \end{aligned}$$

and thus $$\sum _{x,y\in B} xy \otimes \delta _x \otimes \delta _y = \sum _{x\in B} x\otimes \Delta (\delta _x)$$.

This form of the MPO tensor involves thus the tensor product of the irreps $\psi _a$ and $\psi _b$. In Eq. (4.4) we have seen how to decompose such a representation into irreps. Using that equation, the product of the MPO tensors satisfy

**Figure:**
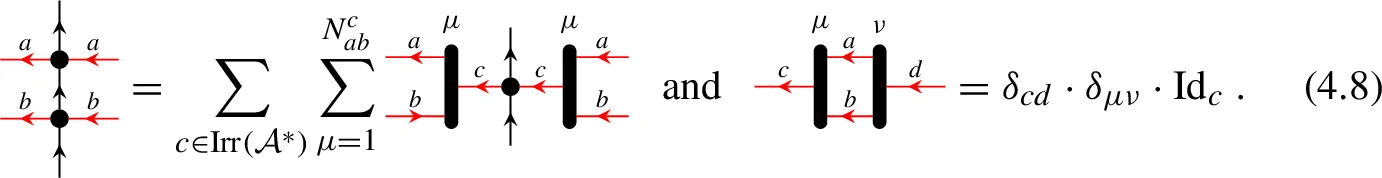

Using Eq. (4.8) in Eq. (4.7), we can finally decompose the r.h.s. of Eq. (4.1) into the sum of injective MPOs:

**Figure:**
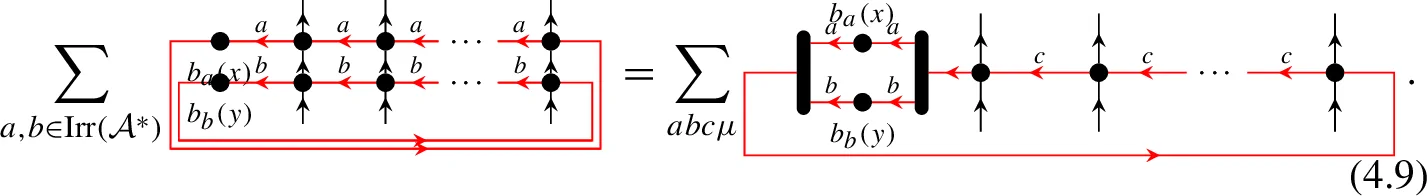

We have thus obtained that the product of two algebra elements *x* and *y* is described by the boundary

**Figure:**
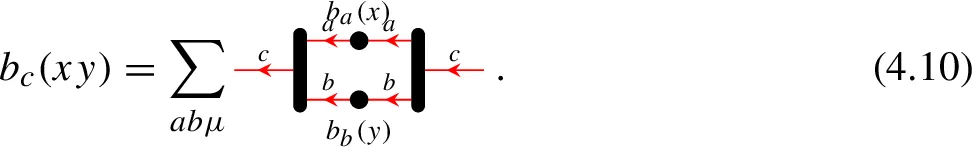

Let us note here that so far we have not investigated how the unit of the pre-bialgebra is represented. The existence of the unit in $\mathcal {A}$, in fact, imposes further restrictions on the structure of the MPOs representing pre-bialgebras. Remember that $\phi ^{\otimes n}\circ \Delta ^{n-1}(1)$ is a non-trivial projector that is represented by:

**Figure:**
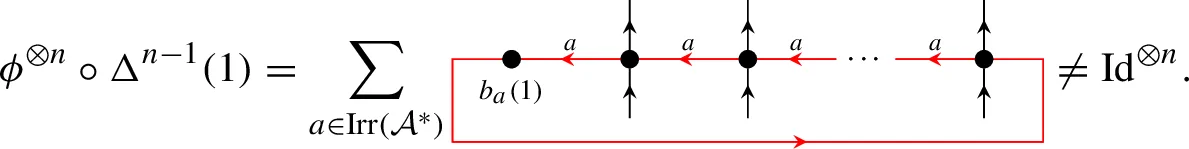

The relation 1x=x1=x, using Eq. (4.10), then implies that the matrices $b_a(1)$ have the following property:

**Figure:**
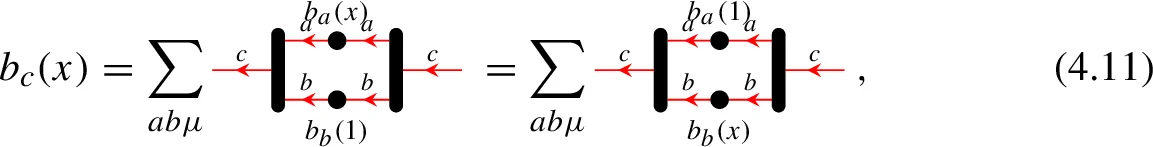

or equivalently,

**Figure:**
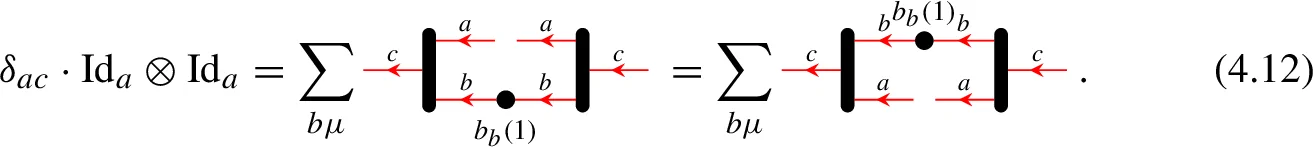

### Cocentral elements and the Grothendieck ring

Let us consider a cosemisimple pre-bialgebra $\mathcal {A}$. Then the cocenter of $\mathcal {A}$, as we have seen in Sect. 3.2, consists of elements *x* of the form

**Figure:**
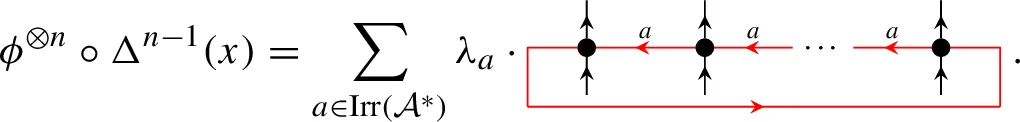

Let $\tau _a\in \mathcal {A}$ be the character of the irrep class $a\in \textrm{Irr}(\mathcal {A}^*)$, i.e. the cocentral element with MPO representation

**Figure:**
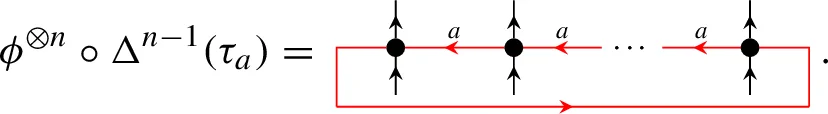

Let us evaluate the product of two irrep characters $\tau _a$ and $\tau _b$. Using the previously derived results, it is immediate to see from their MPO representations that

**Figure:**
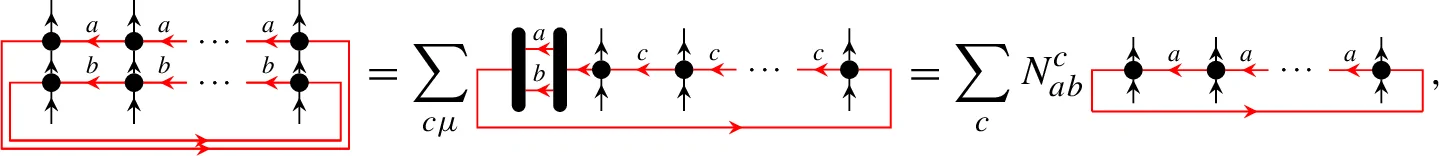

where the first equation is Eq. (4.9) using that $b_a(\tau _c) = \delta _{ac} \textrm{Id}_c$ for all $a,c\in \textrm{Irr}(\mathcal {A}^*)$, and the second is just the orthogonality relations from Eq. (4.8) together with the fact that μ runs from 1 to $N_{ab}^c$. This equation then reads as

$$\begin{aligned} \phi ^{\otimes n} \circ \Delta ^{n-1} (\tau _a) \cdot \phi ^{\otimes n} \circ \Delta ^{n-1} (\tau _a) = \sum _c N_{ab}^c \cdot \phi ^{\otimes n} \circ \Delta ^{n-1} (\tau _c), \end{aligned}$$

and thus it implies, as $\phi ^{\otimes n} \circ \Delta ^{n-1}$ is a homomorphism and it is w.l.o.g. injective, that

4.13 $$\begin{aligned} \tau _a \cdot \tau _b = \sum _c N_{ab}^c \cdot \tau _c. \end{aligned}$$

Let us remark here that to obtain this well-known result, one does not have to consider MPO representations. Instead, the same result can be obtained directly from the decomposition of the tensor product representation into irreps: let $\psi _a$ be an irrep on a vector space $V_a$ such that it is from the irrep class *a* (and thus its character is $\tau _a$), and $\psi _b$ an irrep on a vector space $V_b$ such that it is from the irrep class *b* (and thus its character is $\tau _b$). Then, by definition of the product in $\mathcal {A}^*$,

$$\begin{aligned} (\tau _a \cdot \tau _b)(f) = (\tau _a\otimes \tau _b) \circ \Delta (f) = (\operatorname {Tr}_{V_a} \otimes \operatorname {Tr}_{V_b}) \circ (\psi _a\otimes \psi _b) \circ \Delta (f) = \operatorname {Tr}\left( (\psi _a\otimes \psi _b) \circ \Delta (f) \right) . \end{aligned}$$

In this last trace the operator $(\psi _a\otimes \psi _b) \circ \Delta (f)$ is supported on $V_a \boxtimes V_b$ instead of the whole tensor product space $V_a\otimes V_b$, and thus restricting $(\psi _a\otimes \psi _b) \circ \Delta (f)$ to $V_a \boxtimes V_b$ does not change its trace:

$$\begin{aligned} (\tau _a \cdot \tau _b)(f) = \operatorname {Tr}(\psi _a\otimes \psi _b) \circ \Delta (f) = \operatorname {Tr}(\psi _a\boxtimes \psi _b)(f). \end{aligned}$$

Finally, this representation decomposes into irreps (see Eq. (4.2)), and thus the trace can be evaluated:

$$\begin{aligned} (\tau _a \cdot \tau _b)(f) = \operatorname {Tr}(\psi _a\boxtimes \psi _b)(f) = \sum _c N_{ab}^c \cdot \operatorname {Tr}\psi _c (f) = \sum _c N_{ab}^c \cdot \tau _c (f), \end{aligned}$$

that is equivalent to Eq. (4.13). We have thus seen that

#### Proposition 4.1

In a finite dimensional cosemisimple pre-bialgebra $\mathcal {A}$ over $\mathbb {C}$ the irrep characters of $\mathcal {A}^*$ correspond to the injective blocks in the MPO representation of $\mathcal {A}$. For $a\in \textrm{Irr}(\mathcal {A}^*)$, the irrep character $\tau _a\in \mathcal {A}$ has the following MPO representation:

**Figure:**
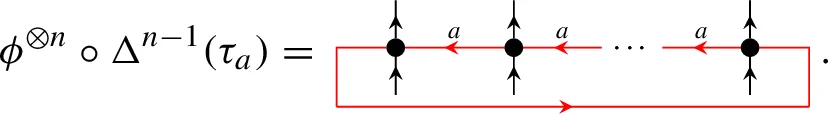

These MPOs form a closed ring^4^ over $\mathbb {Z}$, i.e. for all *a*, *b*, *c* there are non-negative integer numbers $N_{ab}^c$ such that $\tau _a \tau _b = \sum _c N_{ab}^c \tau _c$, or graphically,

**Figure:**
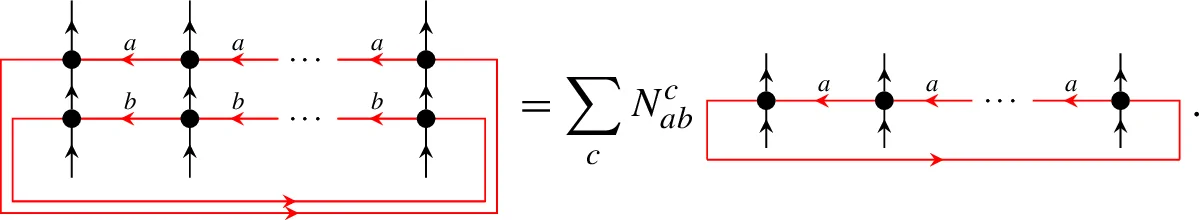

This ring is then called the Grothendieck ring of $\mathcal {A}$.

### Duality

As we have seen before, the dual $$\mathcal {A}^*$$ of a pre-bialgebra $$\mathcal {A}$$ is a pre-bialgebra as well. As such, it also has MPO representations. Let us fix a representation ψ of $$\mathcal {A}^*$$ on a vector space *W*, and a representation ϕ of $$\mathcal {A}^{**} = \mathcal {A}$$ on a vector space *V*. Then the previous construction leads to the following MPO representation:

**Figure:**
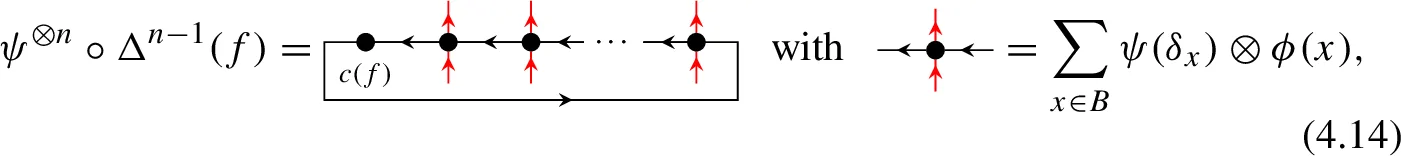

where *B* is a basis of $\mathcal {A}$. Notice that this MPO tensor is exactly 90 degree rotation of the MPO tensor describing the coproduct of elements in $\mathcal {A}$. As above, these MPOs form a representation of $\mathcal {A}^*$,

**Figure:**
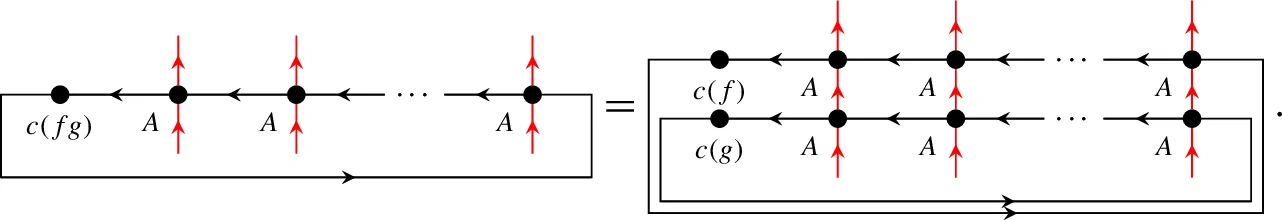

If $\mathcal {A}$ is semisimple (in general this does not follow from semisimplicity of $\mathcal {A}^*$), then this MPO representation of $\mathcal {A}^*$ decomposes into a sum of smaller bond dimensional MPOs corresponding to the characters of $\mathcal {A}$:

**Figure:**
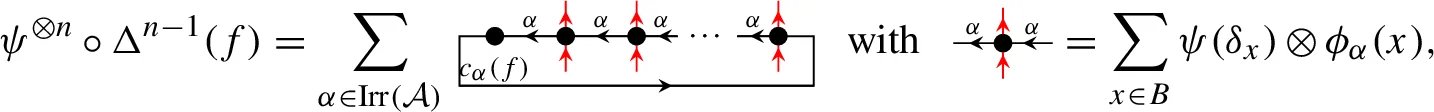

where *B*, as above, is a basis of $\mathcal {A}$. We will denote irrep classes of $\mathcal {A}$ with Greek letters, and irrep classes of $\mathcal {A}^*$ with Latin letters. Just as above, there are linear maps $\hat{V}_{\alpha \beta }^{\gamma m}$ and $\hat{W}_{\alpha \beta }^{\gamma m}$ ($m=1\dots \hat{N}_{\alpha \beta }^\gamma$), such that

**Figure:**
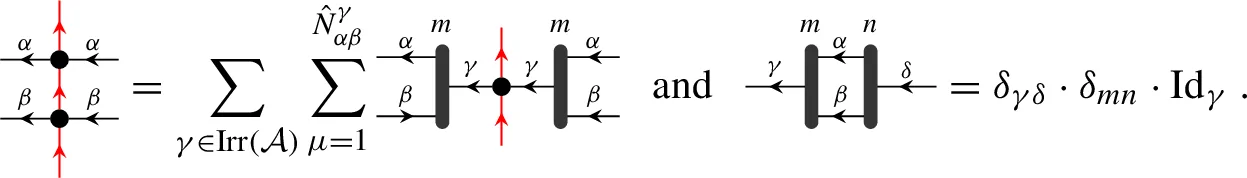

These linear maps give rise to a set of *F*-symbols (also satisfying the pentagon equations) that are, in general, different from the ones in the previous sections.

## Weak Bialgebras and Weak Hopf Algebras

In this section we introduce weak bialgebras, weak Hopf algebras, pivotal weak Hopf algebras, spherical weak Hopf algebras and $C^*$-weak Hopf algebras. All these structures are all special pre-bialgebras, and as such, we use their MPO representations to reason about the structure of these objects; this is possible as the MPO representations are w.l.o.g. injective. Our main result is Theorem 5.1, where we construct a special normalized integral Λ in any cosemisimple weak Hopf algebra over $\mathbb {C}$. This integral Λ is then used to prove that in a cosemisimple co-pivotal weak Hopf algebra over $\mathbb {C}$, there is a cocommutative projector with a property reminiscent to the definition of an integral that will allow us to define MPO-injective PEPS. We then further specialize these results to spherical- and $C^*$-weak Hopf algebras.

### Weak bialgebras

In this section we define weak bialgebras (WBA) and show that the MPO representation of the unit of a cosemisimple WBA $\mathcal {A}$ has the following property: for all irreps *a* of $\mathcal {A}^*$, $b_a(1)$ is either 0 or rank-one. We show, moreover, that the Grothendieck ring of $\mathcal {A}$ has a unit. This unit can be written in the form $\tau _E:= \sum _{e\in E} \tau _e$, where $E\subseteq \textrm{Irr}(\mathcal {A}^*)$ consists of the irreps *a* for which $b_a(1)\ne 0$.

#### Definition 5.1

(Weak bialgebra). A weak bialgebra (WBA) is a pre-bialgebra $\mathcal {A}$ such that the unit $1\in \mathcal {A}$ and counit $\epsilon \in \mathcal {A}^*$ satisfy

5.1 $$\begin{aligned}&\sum _{(1)} 1_{(1)} \otimes 1_{(2)} \otimes 1_{(3)} = \sum _{(1)(1')} 1_{(1)} \otimes 1_{(2)} 1'_{(1)} \otimes 1'_{(2)} = \sum _{(1)(1')} 1_{(1)} \otimes 1'_{(1)} 1_{(2)} \otimes 1'_{(2)}, \end{aligned}$$

5.2 $$\begin{aligned}&\sum _{(\epsilon )} \epsilon _{(1)} \otimes \epsilon _{(2)} \otimes \epsilon _{(3)} = \sum _{(\epsilon )(\epsilon ')} \epsilon _{(1)} \otimes \epsilon _{(2)} \epsilon '_{(1)} \otimes \epsilon '_{(2)} = \sum _{(\epsilon )(\epsilon ')} \epsilon _{(1)} \otimes \epsilon '_{(1)} \epsilon _{(2)} \otimes \epsilon '_{(2)}. \end{aligned}$$

We will refer to Eq. (5.1) as the unit axiom and to Eq. (5.2) as the counit axiom.

In the equations above 1 and ϵ appears twice in the same Sweedler notation. To distinguish between the two appearances, we added a prime to one of them. One can also write the unit axiom, Eq. (5.1), as

$$\begin{aligned} \Delta ^2(1) = (1\otimes \Delta (1)) \cdot (\Delta (1) \otimes 1) = (\Delta (1) \otimes 1) \cdot (1\otimes \Delta (1)), \end{aligned}$$

while the counit axiom, Eq. (5.2), is more convenient to think of as

$$\begin{aligned} \epsilon (xyz) = \sum _{(y)} \epsilon (xy_{(1)}) \epsilon (y_{(2)}z) = \sum _{(y)} \epsilon (xy_{(2)}) \epsilon (y_{(1)}z). \end{aligned}$$

As pre-bialgebras are self-dual and the above two axioms are the dual of one another, weak bialgebras are also self-dual: if $\mathcal {A}$ is a WBA, then $\mathcal {A}^*$ is also a WBA.

Let $\mathcal {A}$ be a cosemisimple WBA. The graphical representation of the unit axiom, Eq. (5.1), is

**Figure:**
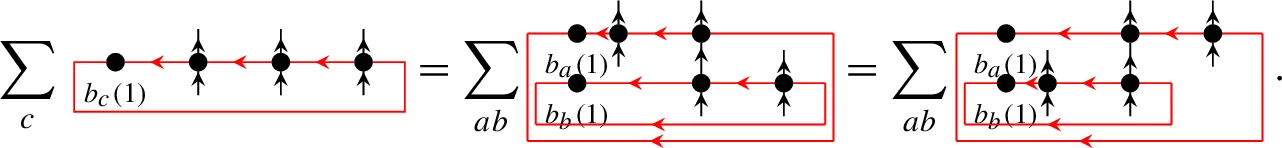

Decomposing the product of the two MPO tensors using the fusion tensors (Eq. (4.8)), we arrive at

**Figure:**
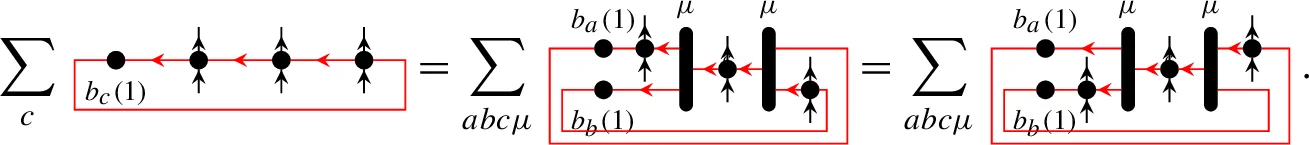

Applying linear functionals *f*, *g* and *h* on the three components, we arrive at the equations

**Figure:**
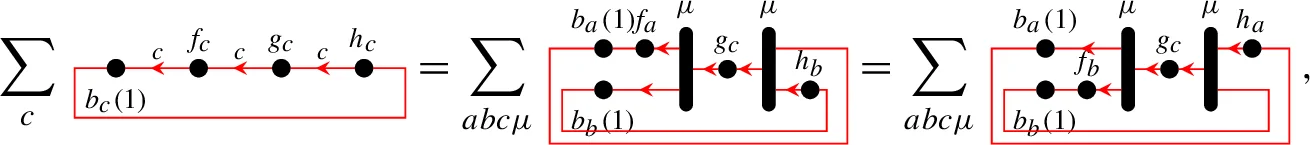

where $f_c$ denotes the part of *f* supported on the irrep sector *c*, i.e. $f_c = p_c f = f p_c$, where $p_c\in Z(\mathcal {A}^*)$ is the irrep projector onto the irrep sector *c*. This equation is true for all *f*, *g* and *h*, and thus, since $\psi _c(\mathcal {A}^*)$ is the full matrix algebra $\mathcal {M}_{D_c}$ for all irrep sectors, we obtain

**Figure:**
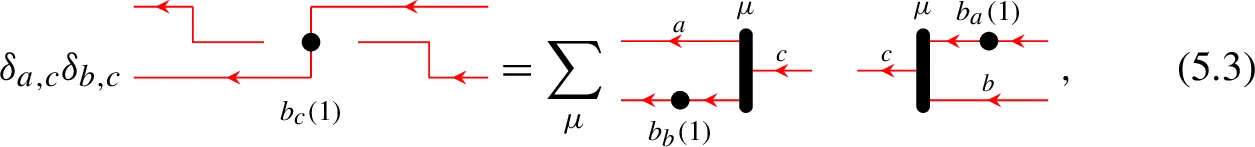

**Figure:**
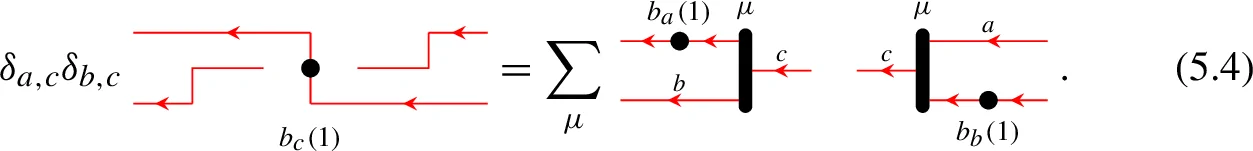

Combining these two equations, we arrive at

**Figure:**
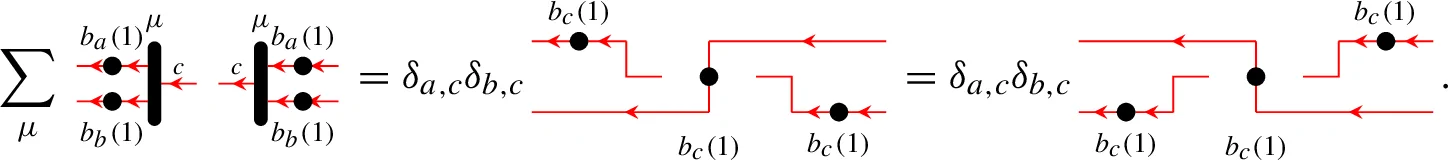

Comparing the last two expressions, we obtain that $b_c(1)$ is rank-1 for all *c* where it is non-zero, i.e. there is a vector  and a linear functional  such that

**Figure:**
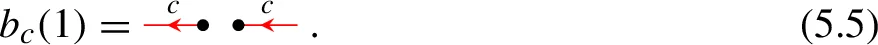

Let *E* be the set of irreps such that $b_c(1)\ne 0$. Let us now consider Eqs. (5.3) and (5.4) such that the irreps *a* and *b* are from this set *E*, while *c* is an arbitrary irrep. Using Eq. (5.5), we obtain

**Figure:**
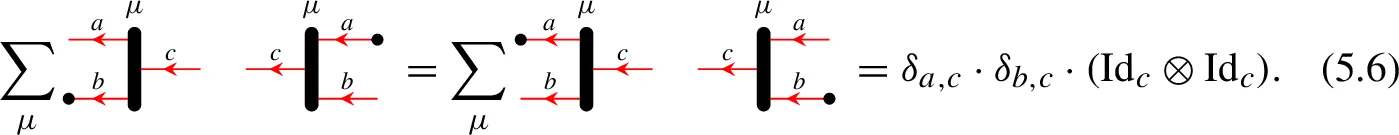

In particular, $N_{ab}^c\ne 0$ for a,b∈E and $c\in \textrm{Irr}(\mathcal {A}^*)$ if and only if a=b=c. As *b*(1) is rank-one in every sector where it is non-zero, the MPO representation of $1\in \mathcal {A}$ can be written as

**Figure:**
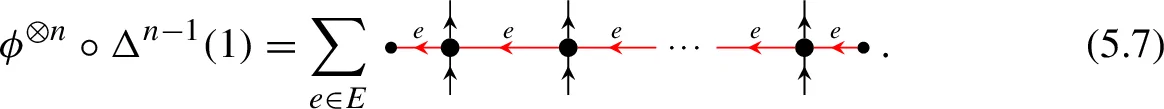

Note that the fact that $1\in \mathcal {A}$ is the unit of the algebra implies, using Eq. (4.12), that for all $a,c\in \textrm{Irr}{\mathcal {A}^*}$,

**Figure:**
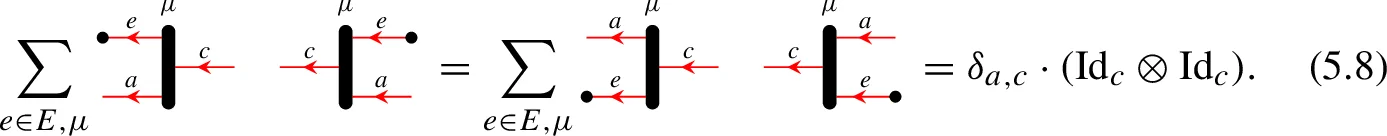

Let us now consider the element $\sum _{(1)} 1_{(1)}1_{(2)}$. The MPS representation of this element is given by

**Figure:**
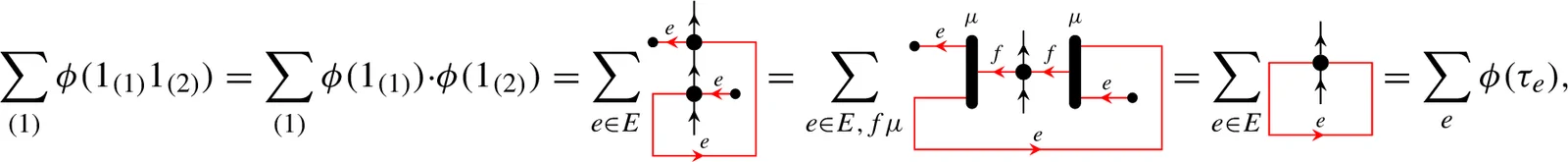

where in the fourth equality we have used Eq. (5.6). As ϕ is injective w.l.o.g., this implies that $\sum _{(1)} 1_{(1)}1_{(2)} = \sum _{e\in E} \tau _e$. Let us denote this element of the algebra as $\tau _E$. Notice now that $\Delta (1) \cdot \Delta _{\textrm{op}}(1)$ is represented by the product

**Figure:**
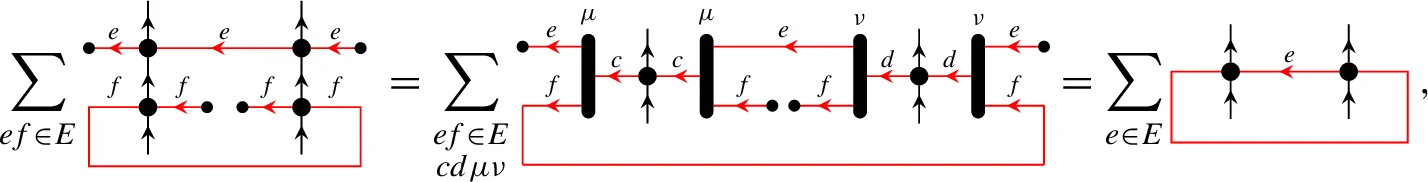

where in the last equation we have used Eq. (5.6). Again, as $\phi \otimes \phi$ is w.l.o.g. injective, we conclude that $\Delta (1) \cdot \Delta _{\textrm{op}}(1) = \Delta (\tau _E)$. Similar calculation shows that $\Delta _{\textrm{op}}(1) \cdot \Delta (1) = \Delta (\tau _E)$ as well, and thus we have proven^5^ that

$$\begin{aligned} \Delta (\tau _E) = \Delta (1) \cdot \Delta _{\textrm{op}}(1) = \Delta _{\textrm{op}}(1) \cdot \Delta (1). \end{aligned}$$

This, in fact, implies that $\tau _E$ is the unit of the Grothendieck ring of $\mathcal {A}^*$ (and of the character algebra as well), as for any co-central $\tau \in \mathcal {A}$,

$$\begin{aligned} \Delta (\tau _E \cdot \tau ) = \Delta _{\textrm{op}}(1) \cdot \Delta (1) \cdot \Delta (\tau ) = \Delta _{\textrm{op}}(1) \cdot \Delta (\tau ) = \Delta _{\textrm{op}}(1)\cdot \Delta _{\textrm{op}}(\tau ) =\Delta _{\textrm{op}}(\tau ) = \Delta (\tau ), \end{aligned}$$

i.e. $\tau _E \cdot \tau = \tau$, and similarly $\tau \cdot \tau _E = \tau$ as well. Notice that as $\tau _E$ decomposes into a sum of irrep characters, given any irrep character $\tau _a$ ($a\in \textrm{Irr}(\mathcal {A}^*)$) the following equations hold:

$$\begin{aligned} \tau _a = \tau _E \tau _a = \sum _{e\in E,b} N_{ea}^b \tau _b \quad \text {and} \quad \tau _a = \tau _a \tau _E = \sum _{e\in E,b} N_{ae}^b \tau _b. \end{aligned}$$

Here both $N_{ea}^b\ge 0$ and $N_{ae}^b\ge 0$ are integers, and thus, as the equations above are equivalent to $\sum _{e\in E} N_{ea}^b =\delta _{ab}$ and $\sum _{e\in E} N_{ae}^b =\delta _{ab}$, there are unique labels $l_a,r_a\in E$ such that for all e∈E and $a,b\in \textrm{Irr}(\mathcal {A}^*)$

5.9 $$\begin{aligned} N_{ea}^b = {\left\{ \begin{array}{ll} 0 & \text {if } e \ne l_a, \\ \delta _{ab} & \text {if } e=l_a, \end{array}\right. } \quad \text {and} \quad N_{ae}^b = {\left\{ \begin{array}{ll} 0 & \text {if } e \ne r_a, \\ \delta _{ab} & \text {if } e=r_a. \end{array}\right. } \end{aligned}$$

Using this property of the fusion multiplicities $N_{ab}^c$, we notice that in Eq. (5.8) in the sum over e∈E the summand is non-zero only for $e=l_a$ on the l.h.s. and $e=r_a$ on the r.h.s. and in these cases the sum over μ is trivial (because $N_{l_aa}^c = N_{ar_a}^c = \delta _{ac}$). We can thus simplify Eq. (5.8) to

**Figure:**
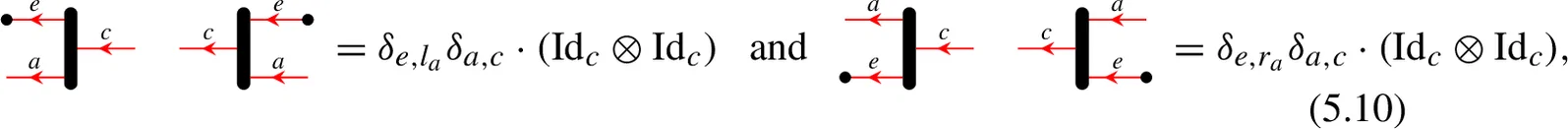

that hold for all e∈E, and $a,c\in \textrm{Irr}(\mathcal {A}^*)$. Let us note that Eq. (5.10) together with Eq. (5.9) implies Eq. (5.6), and thus it is easy to check that the element defined by Eq. (5.7) is indeed the unit of $\mathcal {A}$ and that it satisfies the unit axiom. We have thus seen that

#### Proposition 5.1

In a finite dimensional cosemisimple pre-bialgebra $\mathcal {A}$ over $\mathbb {C}$ the unit axiom Eq. (5.1) is equivalent to the following:

- there is a set $E\subseteq \textrm{Irr}(\mathcal {A}^*)$ such that $\tau _E = \sum _{e\in E} \tau _e$ is the unit of the Grothendieck ring of $\mathcal {A}^*$, or equivalently, for all $a\in \mathcal {A}$ there are unique labels $l_a,r_a\in E$ such that for all e∈E and $b\in \textrm{Irr}(\mathcal {A}^*)$
  $$\begin{aligned} N_{ea}^b = {\left\{ \begin{array}{ll} 0 & \text {if } e \ne l_a, \\ \delta _{ab} & \text {if } e=l_a, \end{array}\right. } \quad \text {and} \quad N_{ae}^b = {\left\{ \begin{array}{ll} 0 & \text {if } e \ne r_a, \\ \delta _{ab} & \text {if } e=r_a, \end{array}\right. } \end{aligned}$$
- and for all e∈E there are vectors  and linear functionals  such that for all e∈E and $a,c\in \textrm{Irr}(\mathcal {A}^*)$,

**Figure:**
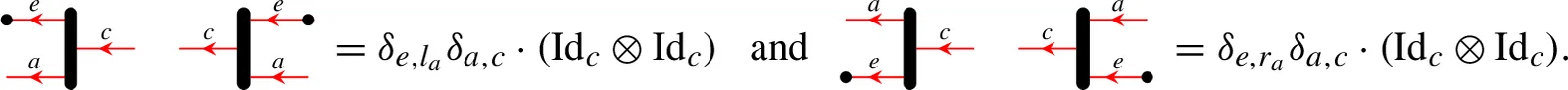

We will refer to the subset *E* of $\textrm{Irr}(\mathcal {A}^*)$ as *vacuum* and the irreps in *E* as *vacuum irrep*.

Let us now apply Eq. (5.9) for the case that a∈E. As in this case both equations in Eq. (5.9) apply, we obtain that for all $a\in \textrm{Irr}(\mathcal {A})$ and e,f∈E,

5.11 $$\begin{aligned} N_{ef}^a = {\left\{ \begin{array}{ll} 1 & \text {if } e=f=a,\\ 0 & \text {otherwise.} \end{array}\right. } \end{aligned}$$

Stating otherwise, we have obtained that for any e∈E, $l_e=r_e=e$ holds. Note the difference between the MPO representation of the unit of the algebra and the element $\tau _E$. The MPO representation of the unit of the algebra $1\in \mathcal {A}$ is

**Figure:**
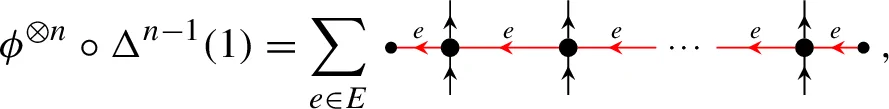

and the MPO representation of the unit of the Grothendieck ring, $\tau _E$, is

**Figure:**
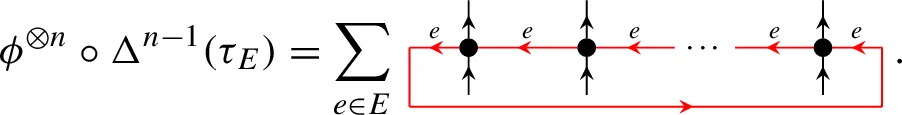

These two MPOs coincide if and only if the irrep *e* is one-dimensional for all e∈E.

Until now, we have only used the unit axiom, and not the counit axiom: the unit $\tau _E\in \mathcal {A}$ of the Grothendieck ring exists and the MPO representation of the unit $1\in \mathcal {A}$ is rank-one in each injective block even for pre-bialgebras that satisfy the unit axiom but not the counit axiom. Let us now derive a consequence of the counit axiom that we will use later on. First, note that the counit axiom can equally be written as

$$\begin{aligned} \Delta ^2(\epsilon ) = (1\otimes \Delta (\epsilon )) \cdot (\Delta (\epsilon ) \otimes \epsilon ) = (\Delta (\epsilon ) \otimes \epsilon ) \cdot (\epsilon \otimes \Delta (\epsilon )), \end{aligned}$$

and therefore the graphical representation of the counit axiom is

**Figure:**
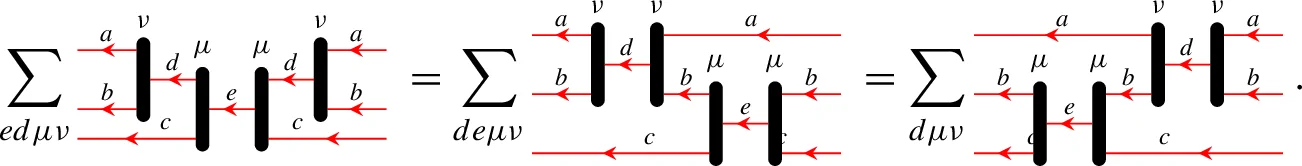

Using now the orthogonality relations in the first equation, we conclude that the following equation holds:

**Figure:**
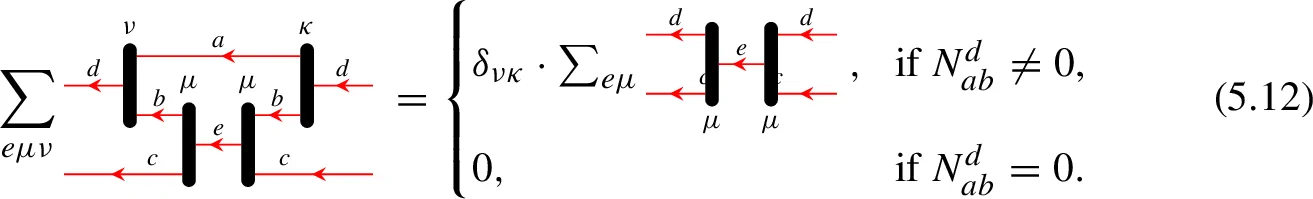

This equation, however, is not a sufficient condition for the unit of the pre-bialgebra to satisfy the counit axiom Eq. (5.2).

### Weak Hopf algebras

In this section we define weak Hopf algebras. In these algebras we define as set of elements called integrals, and show that a cosemisimple weak Hopf algebra over $\mathbb {C}$ has a special integral Λ such that is a projector and is such that $\Lambda (fg) = \Lambda (S^2(g)f)$ holds for all $f,g\in \mathcal {A}^*$.

#### Definition 5.2

(Weak Hopf algebra). A weak Hopf algebra (WHA) is a WBA $\mathcal {A}$ together with a linear map $S:\mathcal {A}\rightarrow \mathcal {A}$, called antipode, such that

$$\begin{aligned} \sum _{(x)} S(x_{(1)})x_{(2)}= \sum _{(1)} 1_{(1)} \epsilon (x1_{(2)}),\\ \sum _{(x)} x_{(1)}S(x_{(2)})= \sum _{(1)} \epsilon ( 1_{(1)} x) 1_{(2)},\\ \sum _{(x)} S(x_{(1)})x_{(2)}S(x_{(3)}) = S(x). \end{aligned}$$

Given a WHA $\mathcal {A}$ with antipode $S_{\mathcal {A}}$, it is easy to check that the map $S_{\mathcal {A}^*}:\mathcal {A}^* \rightarrow \mathcal {A}^*$ defined by

5.13 $$\begin{aligned} S_{\mathcal {A}^*}(f) = f\circ S_{\mathcal {A}}, \end{aligned}$$

for all $f\in \mathcal {A}^*$ (i.e. $S_{\mathcal {A}^*} = S_{\mathcal {A}}^T$), satisfies the antipode axioms as well, and thus $\mathcal {A}^*$ is a WHA too. From now on, we do not differentiate between the antipode of $\mathcal {A}$ and that of $\mathcal {A}^*$, and denote both by *S*. The antipode *S* of a WHA is an anti-homomorphism (S(xy)=S(y)S(x)), an anti-cohomomorphism ($\Delta \circ S = (S\otimes S) \circ \Delta _{\textrm{op}}$) and a bijection (see [BNS99] for a proof). In fact, an equivalent characterization of the antipode (of $\mathcal {A}^*$) is that it is a bijective anti-homomorphism of $\mathcal {A}^*$ such that for every $f\in \mathcal {A}^*$

5.14 $$\begin{aligned} \sum _{(f)} S(f_{(1)}) f_{(2)} \otimes f_{(3)} = \epsilon _{(1)} \otimes f \epsilon _{(2)}, \end{aligned}$$

5.15 $$\begin{aligned} \sum _{(f)} f_{(1)} \otimes f_{(2)} S(f_{(3)}) = \epsilon _{(1)} f \otimes \epsilon _{(2)}. \end{aligned}$$

Let $\mathcal {A}$ be a cosemisimple WHA and $\psi :\mathcal {A}^*\rightarrow \textrm{End}(W)$ be a representation of $\mathcal {A}^*$ on a vector space *W*. The antipode *S* of $\mathcal {A}^*$ is an anti-homomorphism, and thus the linear map $\bar{\psi }:\mathcal {A}^* \rightarrow \textrm{End}(W^*)$ defined by

$$\begin{aligned} \bar{\psi }(f) =(\psi \circ S(f))^T \end{aligned}$$

is a representation of $\mathcal {A}^*$ on the vector space $W^*$. As *S* is a bijection, $\bar{\psi }$ is an irrep if ψ is an irrep. Let $\textrm{Irr}(\mathcal {A}^*)$, as above, denote the irrep equivalence classes of $\mathcal {A}^*$ and for every $a\in \textrm{Irr}(\mathcal {A}^*)$ let us fix a representation $\psi _a$ on the vector space $V_a$ from the irrep class *a*. For each irrep $\psi _a$ the representation $\bar{\psi }_a$ is an irrep in another class, that we denote by $\bar{a}$ (note that $\bar{a}$ might coincide with *a*). As $\bar{\psi }_a$ and $\psi _{\bar{a}}$ are in the same irrep class, they are related to each other by a basis transformation, i.e. there are linear maps $Z_{a}: W_{\bar{a}} \rightarrow W_{a}^*$ such that

$$\begin{aligned} \overline{\psi _a}(f) = Z_{a} \cdot \psi _{\bar{a}}(f)\cdot Z_{a}^{-1}. \end{aligned}$$

Let us denote this equation using the graphical notation of tensor networks. We have to be careful with the notation, as $\overline{\psi _a}(f)$ is not one of the representations that we have fixed previously. We thus have to use a more verbose notation and display the representation itself and that it is a linear map from $W_a^*$ to $W_a^*$:

**Figure:**
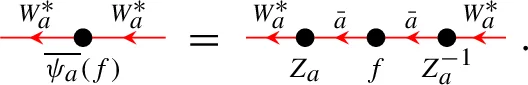

Let us now reverse the two arrows at the two ends of the figure. This changes $W_a^*$ to $W_a$, and thus, as it is one of the previously fixed vector spaces, we can simply write *a* on the line. Similarly, $\overline{\psi _a}(f)$ changes to $\overline{\psi _a}(f)^T = \psi _a\circ S(f)$, and thus, as $\psi _a$ is one of the previously fixed representations, the rank-two tensor on the l.h.s. of the equation can simply be labeled by *S*(*f*):

**Figure:**
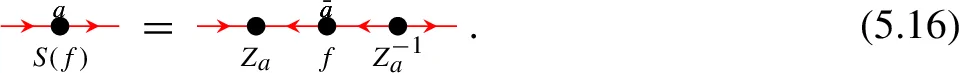

The indices of *S*(*f*) are now oriented in the opposite direction as that of *f*. Because of this, we often bend back the indices on the r.h.s. of this equation such that we obtain

**Figure:**
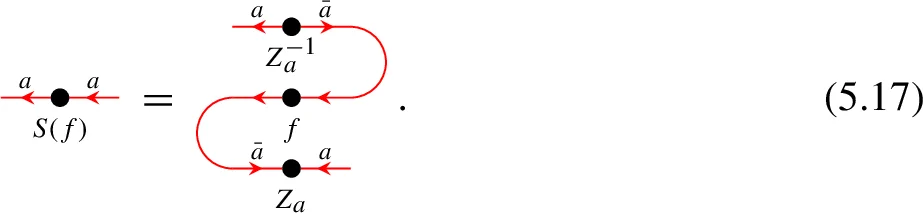

Let us warn here the reader to the subtleties of our notation. First, $Z_a$ has two incoming indices, and thus the only way to differentiate between the two indices is via the labels *a* and $\bar{a}$. Note that even for irrep labels for which $a=\bar{a}$, this forces us to write *a* and $\bar{a}$ on the two legs of the tensor. Second, there are two different operators with very similar graphical representations:

**Figure:**
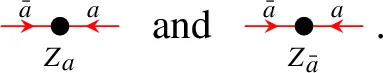

These two tensors should be read in the opposite direction: the first index of $Z_a$ is the one labeled by $\bar{a}$ on its left, while the first index of $Z_{\bar{a}}$ is the one labeled by *a* on its right.

As we have mentioned above, the antipode of $\mathcal {A}$ is the transpose of the antipode of $\mathcal {A}^*$, see Eq. (5.13). Therefore for all $f\in \mathcal {A}^*$ and $x\in \mathcal {A}$ the following holds:

**Figure:**
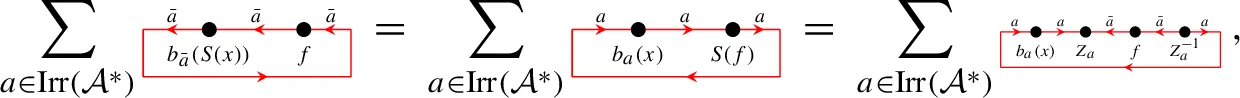

where in the second equation we have used Eq. (5.16). As this equation holds for all $f\in \mathcal {A}^*$, the boundary condition *b*(*S*(*x*)) describing *S*(*x*) can be expressed as

**Figure:**
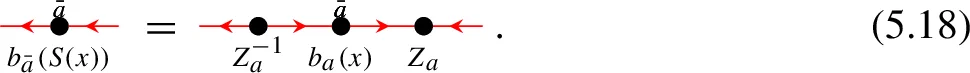

This allows us to write the repeated coproduct of *S*(*x*) as

**Figure:**
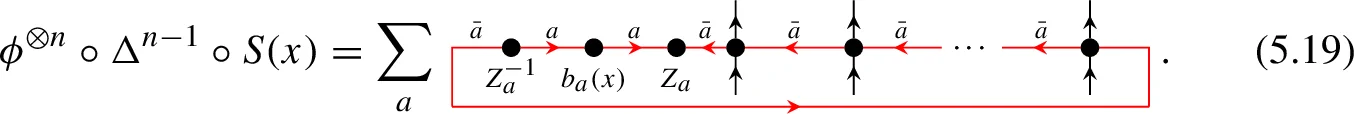

Let us consider now a new rank-4 tensor, denoted by gray dot, defined by

**Figure:**
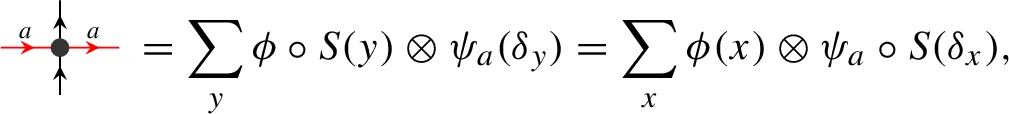

where in the second equation we have used that if *B* is a basis with basis elements *y*, then the set $x=S(y) \ (y\in B)$ forms a basis as well and its dual basis is $\delta _x = S^{-1}(\delta _y) \ (y\in B)$. Note that we have oriented the virtual indices of this tensor in the opposite direction as in the MPO tensor given by black dots. This is because *S* is an anti-homomorphism; more precisely, we can relate the two tensors using Eq. (5.16):

**Figure:**
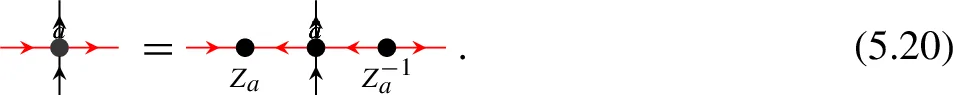

Using this gray tensor, one can rewrite Eq. (5.19) as

**Figure:**
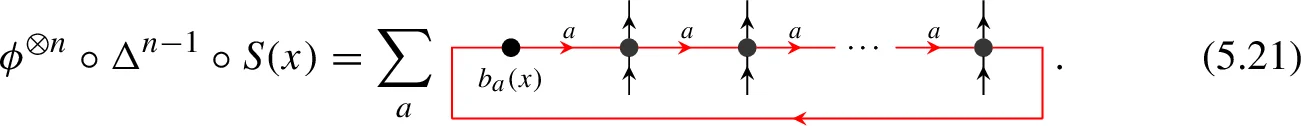

This MPO, by definition, is $\phi ^{\otimes n} \circ S^{\otimes n} \circ \Delta _{\textrm{op}}^{n-1} (x)$ (read from left to right), as the virtual index of the MPO tensor is written in the opposite direction than as usual. As ϕ is w.l.o.g. injective, we have obtained the relation

$$\begin{aligned} \Delta ^{n-1} \circ S (x) = S^{\otimes n}\circ \Delta _{\textrm{op}}^{n-1} (x), \end{aligned}$$

that is, in fact, a simple consequence of the fact that *S* is an anti-cohomomorphism ($\Delta \circ S = (S\otimes S) \circ \Delta _{\textrm{op}}$).

Using the tensors $Z_a$ that we defined in Eq. (5.16), the antipode axioms of $\mathcal {A}^*$, Eqs. (5.14) and (5.15), can be expressed as

**Figure:**
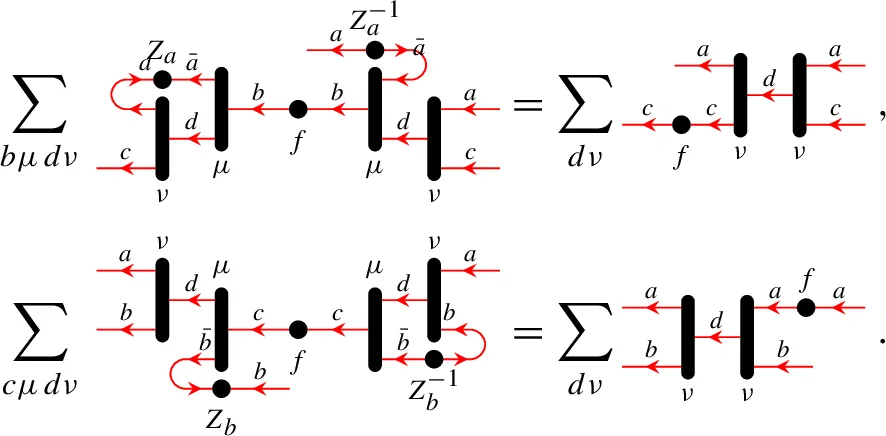

Let us explain briefly how to arrive at these graphical representations of Eq. (5.14) and Eq. (5.15). In Eq. (5.14), on the l.h.s. we first need to take two repeated coproduct of *f*: this is achieved by the two pairs of fusion tensors (see Eq. (4.4)). Then the antipode of the first component is taken (see Eq. (5.17)) and it is multiplied with the second component of the coproduct. On the r.h.s. of the same equation, we multiply *f* with the second component of $\Delta (\epsilon )$ (see Eq. (4.5)). The second equation is obtained similarly. As these equations hold for any $f \in \mathcal {A}^*$, we conclude that

**Figure:**
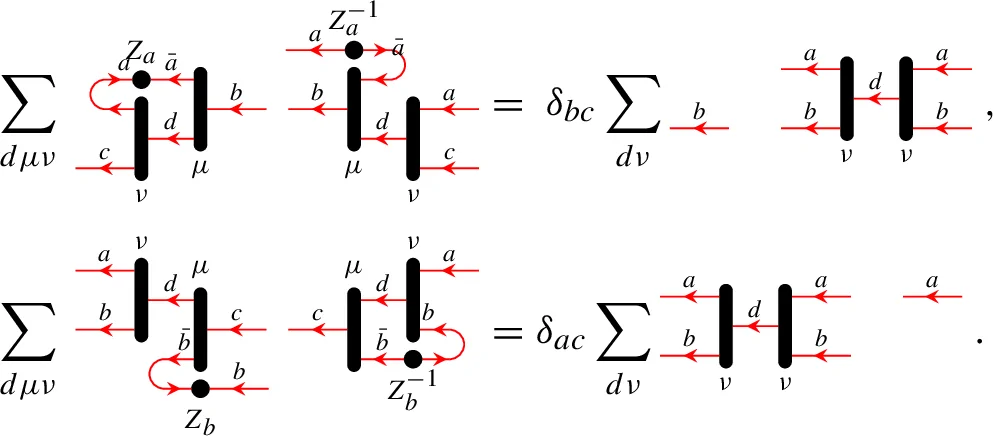

Using the orthogonality relations of the fusion tensors and multiplying the first equation by $Z_{a}$, and the second equation by $Z_{b}^{-1}$, we obtain

**Figure:**
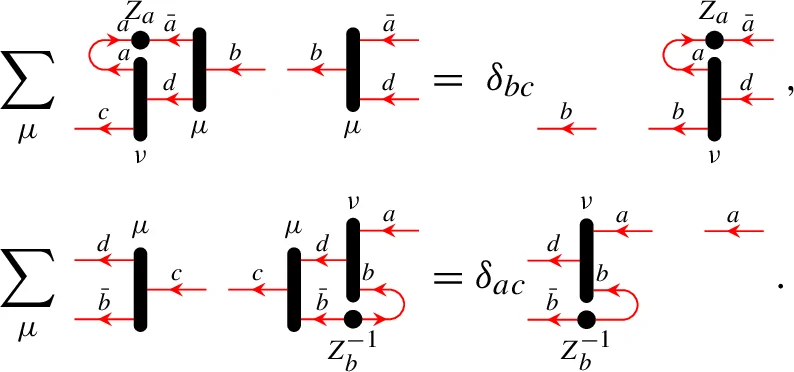

In particular, there are complex matrices $C_{ab}^d$ of size $N_{ab}^d \times N_{\bar{a}d}^b$ and $\hat{C}_{ab}^d$ of size $N_{ab}^d\times N_{d\bar{b}}^a$ such that

**Figure:**
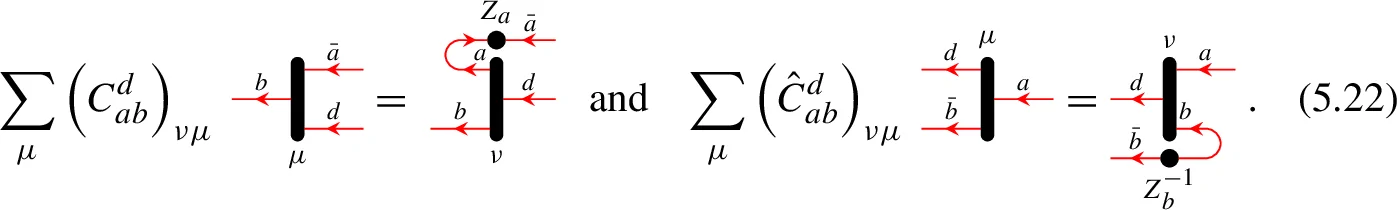

Let us now show that $C_{ab}^d$ and $\hat{C}_{ab}^d$ are both invertible. In both equations the tensors on both sides are linearly independent, therefore $N_{ab}^d\le N_{\bar{a}d}^b$ and $N_{ab}^d \le N_{d\bar{b}}^a$. Applying these inequalities twice, we obtain that $N_{ab}^d\le N_{\bar{a}d}^b \le N_{\bar{\bar{a}}b}^d$, and that $N_{ab}^d \le N_{d\bar{b}}^d \le N_{a\bar{\bar{b}}}^d$. As $a\mapsto \bar{\bar{a}}$ is a permutation of $\textrm{Irr}(\mathcal {A}^*)$, repeating these equations a few^6^ times we obtain that $N_{ab}^d\le N_{\bar{\bar{a}}b}^d\le \dots \le N_{ab}^d$ and that $N_{ab}^d \le N_{a\bar{\bar{b}}}^d \le \dots \le N_{ab}^d$, i.e. in all of the inequalities above equality holds. In particular, for all $a,b,c\in \textrm{Irr}(\mathcal {A}^*)$, the numbers $N_{ab}^d$ possess the following symmetries:

$$\begin{aligned} N_{ab}^d = N_{\bar{a}d}^b \quad \text {and} \quad N_{ab}^d = N_{d\bar{b}}^a. \end{aligned}$$

This implies that in both equation in Eq. (5.22) we transform $N_{ab}^d$ linearly independent vectors to $N_{ab}^d$ linearly independent vectors, i.e. both $C_{ab}^d$ and $\hat{C}_{ab}^d$ are invertible. It is easy to check that the argument above can be repeated backwards, i.e. Eq. (5.22) implies the antipode axioms. We have thus seen that

#### Proposition 5.2

Let $\mathcal {A}$ be a finite dimensional cosemisimple WBA. Then the map $S:\mathcal {A}^*\rightarrow \mathcal {A}^*$ given by

**Figure:**
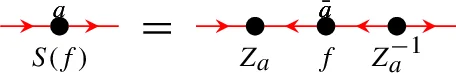

is an antipode making $\mathcal {A}^*$ a WHA if and only if there are invertible matrices $C_{ab}^d$ of size $N_{ab}^d \times N_{\bar{a}d}^b$ and $\hat{C}_{ab}^d$ of size $N_{ab}^d\times N_{d\bar{b}}^a$ such that

**Figure:**
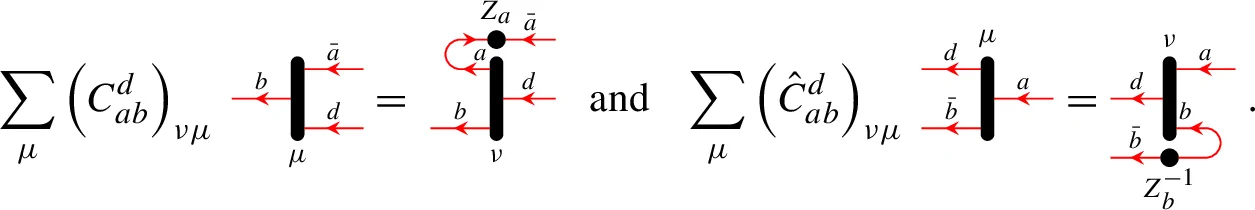

As it is interesting on its own, let us restate here the symmetry properties of the fusion multiplicities $N_{ab}^c$ together with some of the consequences of these symmetries:

#### Proposition 5.3

Let $\mathcal {A}$ be a finite dimensional cosemisimple weak Hopf algebra, and $a,b,c\in \textrm{Irr}(\mathcal {A}^*)$. Then

5.23 $$\begin{aligned} N_{ab}^d = N_{\bar{a}d}^b \quad \text {and} \quad N_{ab}^d = N_{d\bar{b}}^a. \end{aligned}$$

Moreover, the following equations hold:

- $$\bar{\bar{a}} = a$$ for all $$a\in \textrm{Irr}(\mathcal {A}^*)$$,
- $l_a = r_{\bar{a}}$ and $r_a=l_{\bar{a}}$ for all $a\in \textrm{Irr}(\mathcal {A}^*)$, where $l_a,r_a\in E$ are as in Eq. (5.9),
- $$e = \bar{e}$$ for all e∈E.

#### Proof

We have already proven Eq. (5.23) above. Let us now show that $a=\bar{\bar{a}}$ for all irrep labels $a\in \textrm{Irr}(\mathcal {A}^*)$. Applying the left side of Eq. (5.23) twice with d=a and $b=r_a$, we obtain that

$$\begin{aligned} 1 = N_{ar_a}^a = N_{\bar{a}a}^{r_a} = N_{\bar{\bar{a}}r_a}^a. \end{aligned}$$

Using now Eq. (5.9), this latter fusion multiplicity can only be non-zero if $a = \bar{\bar{a}}$.

Let us now show that for all $a\in \textrm{Irr}(\mathcal {A})$, $l_{\bar{a}} = r_a$ holds. Using the left equation in Eq. (5.23) with $b=r_a$ and d=a, we obtain that

$$\begin{aligned} 1 = N_{ar_a}^a = N_{\bar{a}a}^{r_a} = N_{r_a\bar{a}}^{\bar{a}}, \end{aligned}$$

where the last equation is the right equation in Eq. (5.23). As $r_a\in E$ and the above fusion number is non-zero, Eq. (5.9) implies that for all $a\in \textrm{Irr}(\mathcal {A})$, $r_a = l_{\bar{a}}$. The equation $r_{\bar{a}} = l_{a}$ can be shown in a similar way.

Let us finally prove that the permutation $a\mapsto \bar{a}$ leaves the set *E* invariant, and in fact for every e∈E, $\bar{e} = e$. Let us use Eq. (5.23) with a=b=d=e∈E. We obtain that

$$\begin{aligned} 1 = N_{ee}^e = N_{\bar{e}e}^e, \end{aligned}$$

and thus, as e∈E, using Eq. (5.9), this latter fusion number can only be non-zero if $e= \bar{e}$. □

Let us show now that in a cosemisimple WHA the matrices $Z_a$ can be expressed with the help of the fusion tensors and the unit of the underlying WBA. Therefore, if in a cosemisimple WBA there exists an antipode making it a WHA, this antipode is uniquely determined by the WBA structure. This statement, in fact, is more general than what we show here, and also holds in the non-cosemisimple case (see e.g. [BNS99]).

Let $c\in \textrm{Irr}(\mathcal {A}^*)$ and let us set $\bar{a}=\bar{c}$, d=c and $b=r_c\in E$ in the left equation in Eq. (5.22), and $d=\bar{c}$, $b = \bar{c}$ and $a=r_c\in E$ in the right equation in Eq. (5.22) and use that $\bar{\bar{c}} = c$. As $N_{\bar{c}c}^{r_{c}} = 1$, we obtain that there are non-zero complex numbers $C_{cr_c}^c$ and $\hat{C}_{r_c\bar{c}}^{\bar{c}}$ such that

**Figure:**
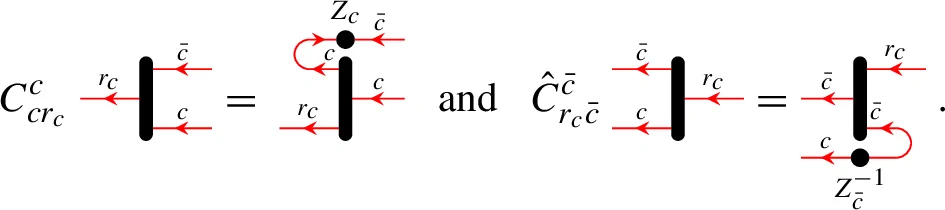

Taking the tensor product of these two equations, applying the vectors describing $b_{r_c}(1)$ and using Eq. (5.10), we conclude that there are non-zero numbers $w_c$ such that

**Figure:**
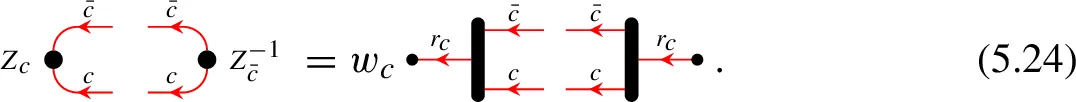

We have thus obtained that the matrices $Z_c$ can be expressed with the help of the fusion tensors and the vectors describing the unit of the WBA, and thus the antipode of a cosemisimple WHA is unique and completely determined by the WBA structure.

Let us now define a linear functional that will play a central role in the rest of the paper. Before the definition, note that while the number $w_c$ defined in Eq. (5.24) depend on the concrete choice^7^ of the matrices $Z_c$ and $Z_{\bar{c}}^{-1}$, the number $w_c w_{\bar{c}}$ is independent from this choice, and it is also independent from all the choices we have made before (the fusion tensors and the vectors describing the unit of the algebra). This is because all these objects appear in pairs in the following equation:

**Figure:**
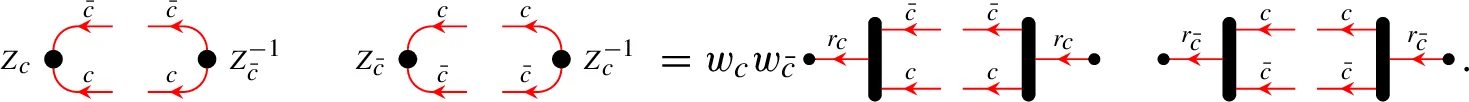

Let $d_a$ be complex numbers such that $d_a^2 = w_a w_{\bar{a}}$ and $d_a = d_{\bar{a}}$. Later we will prove that $w_aw_{\bar{a}}>0$ and thus one can choose $d_a$ to be positive. Until then, $d_a=d_{\bar{a}}$ is an arbitrary square root of $w_aw_{\bar{a}}$. Let us now define the linear functional $g\in \mathcal {A}^*$ through

**Figure:**
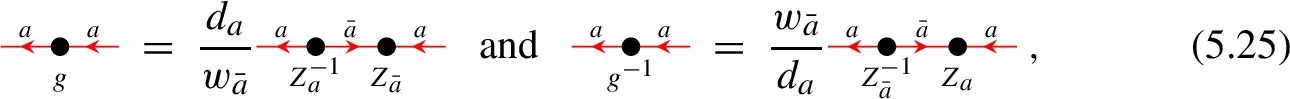

where we have used that *f* for any matrix *m* of the form $\bigoplus _a m_a$, $m_a\in \textrm{End}(V_a)$, one can find a linear functional such that $\psi _a(f) = m_a$. The linear functional *g* defined in this way is independent of all previous choices that we have made, except for the choice of the square root $d_a=d_{\bar{a}}$ of $d_a^2$. The importance of this linear functional is that the conjugation by it describes the action of $S^2$. Indeed, applying Eq. (5.16) twice, we obtain that

**Figure:**
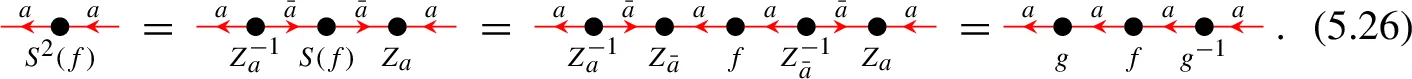

Let us now show a few additional properties of *g*. First, note that $w_{\bar{a}}/d_a = d_a/w_a = d_{\bar{a}}/w_a$, and thus

**Figure:**
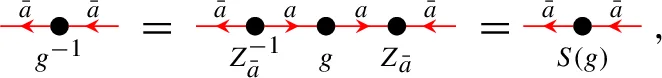

i.e. *g* satisfies $g^{-1} = S(g)$. Second, the traces of $\psi _a(g)$ and $\psi _a(g^{-1})$ evaluate to the following:

**Figure:**
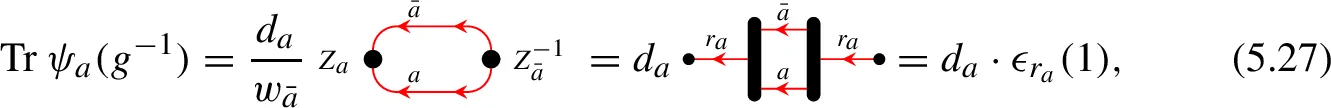

**Figure:**
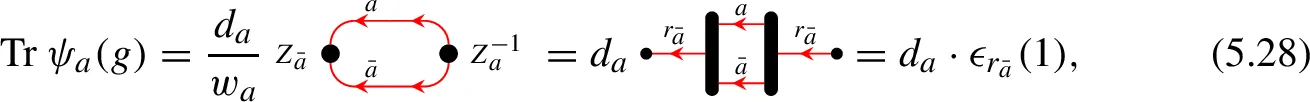

where $\epsilon _{r_a}\in \mathcal {A}^*$ is the irrep projector onto the irrep sector $r_a$ and $\epsilon _{r_{\bar{a}}}\in \mathcal {A}^*$ is the irrep projector onto the irrep sector $r_{\bar{a}}$.

In the following, we will encounter algebra elements of the form $x = \sum _a (\textrm{Id}\otimes g) \circ \Delta (\tau _a)$. These algebra elements have the following MPO representation:

**Figure:**
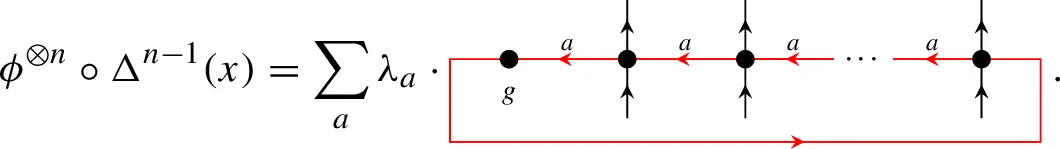

The algebra elements are exactly those that satisfy $\Delta _{\textrm{op}}(x) = (S^{-1}\otimes \textrm{Id})\circ \Delta (x)$. Indeed,

**Figure:**
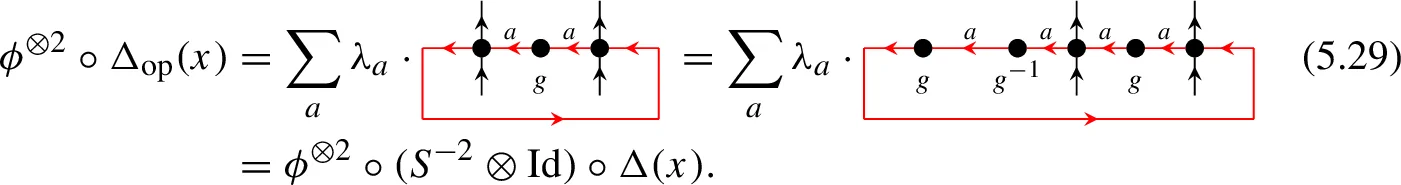

One can easily see that all elements that satisfy the above cocommutation relation are in fact of this form. Such algebra elements will be called *q-traces*:

#### Definition 5.3

(Q-trace). Let $\mathcal {A}$ be a weak Hopf algebra. The algebra element $x\in \mathcal {A}$ is called a q-trace if

$$\begin{aligned} \Delta _{\textrm{op}}(x) = (S^{-2} \otimes \textrm{Id}) \circ \Delta (x). \end{aligned}$$

In the following, we will construct an algebra element that is not only a q-trace, but it is also a *left integral*:

#### Definition 5.4

(Integrals). A left integral of a WHA $\mathcal {A}$ is an element $\Lambda \in \mathcal {A}$ such that for all $x\in \mathcal {A}$

$$\begin{aligned} (1\otimes x) \cdot \Delta (\Lambda ) = (S(x)\otimes 1) \cdot \Delta (\Lambda ). \end{aligned}$$

The element $\Lambda \in \mathcal {A}$ is called a right integral if S(Λ) is a left integral. An integral that is both left and right is a two-sided integral. A non-degenerate (see Definition 3.5) integral Λ is normalized if $\Lambda ^2 = \Lambda$. Finally, an integral is called a Haar integral if it is two-sided, non-degenerate and normalized.

We will now prove the main result of this section: that in a cosemisimple WHA there is a special integral that is non-degenerate, normalized and a q-trace:

#### Theorem 5.1

In a finite dimensional cosemisimple WHA $\mathcal {A}$ over $\mathbb {C}$ the element $\Lambda \in \mathcal {A}$ defined^8^ by

**Figure:**
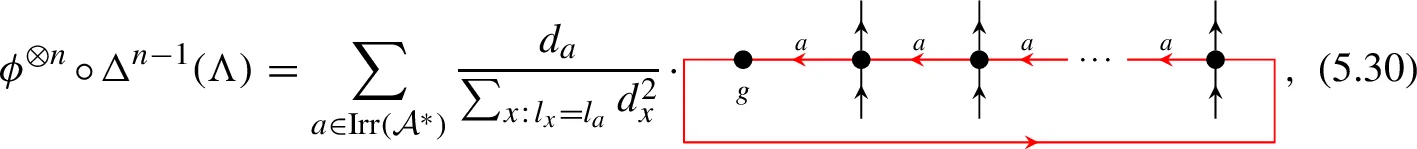

is a non-degenerate normalized left integral that is also a q-trace.

In the proof of Theorem 5.1 we will use the following two lemmas that we prove in Sect. A:

#### Lemma 5.1

Let $\mathcal {A}$ be a cosemisimple WHA, and *g* be the linear functional defined in Eq. (5.25). Then there exists an $N_{ab}^c\times N_{ab}^c$ matrix $B_{ab}^c$ such that the linear functional $g\in \mathcal {A}^*$ satisfies

**Figure:**
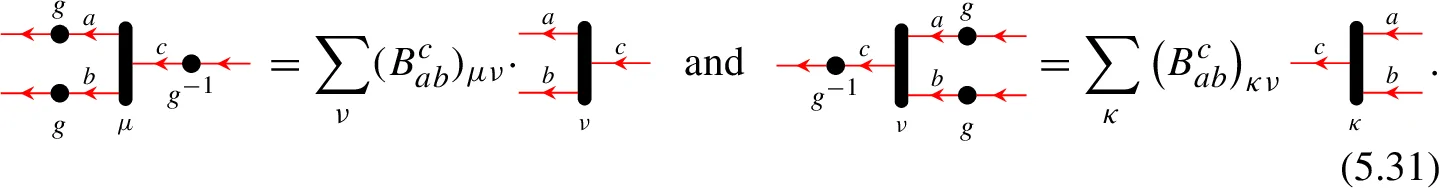

Moreover, $\left( B_{ab}^c\right) ^2 = \textrm{Id}_{N_{ab}^c}$ and the following equation holds as well:

**Figure:**
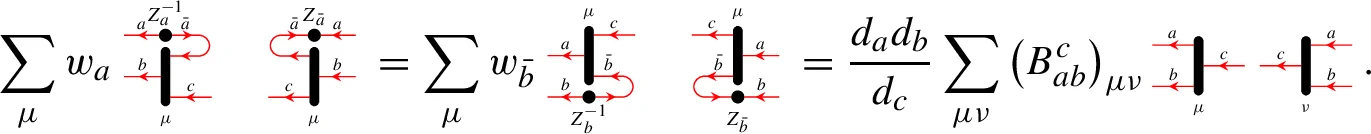

Investigating the trace of $B_{ab}^c$ we can prove that the numbers $d_a^2$ are positive and thus that the denominator in the r.h.s. of Eq. (5.30) is non-zero. We postpone the proof of this lemma to Sect. A as well.

#### Lemma 5.2

For all $a\in \textrm{Irr}(\mathcal {A}^*)$, $d_a^2 = w_a w_{\bar{a}}>0$. Let moreover $T_{ab}^c$ be defined by $T_{ab}^c = \sum _{\mu } \left( B_{ab}^c\right) _{\mu \mu }$. Then the following equations hold:

$$\begin{aligned} \sum _b T_{ab}^c \cdot d_b = d_a \cdot \delta _{l_a l_c} \cdot d_c \quad \text {and} \quad \sum _{x:l_x = l_a} d_x^2 = \sum _{x:l_x =r_a} d_x^2. \end{aligned}$$

With these two lemmas in hand, we can proceed to the proof of Theorem 5.1.

#### Proof of Theorem 5.1

Let us define *L* as

**Figure:**
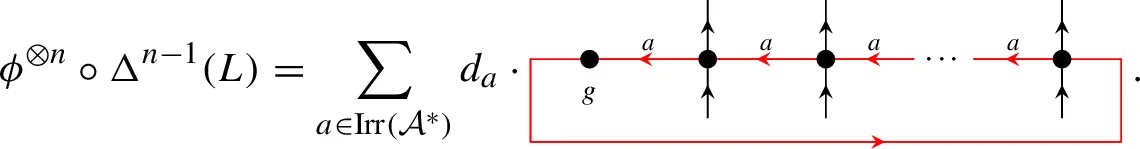

We will prove that *L* a non-degenerate left integral that is related to Λ as follows. Let us define *N* via

**Figure:**
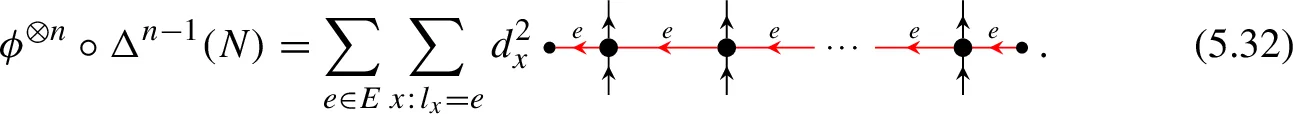

This algebra element *N* is central, as *Nx* is described by the MPO

**Figure:**
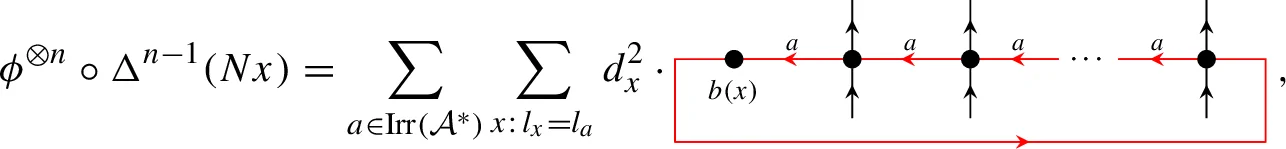

while *xN* is described by the MPO

**Figure:**
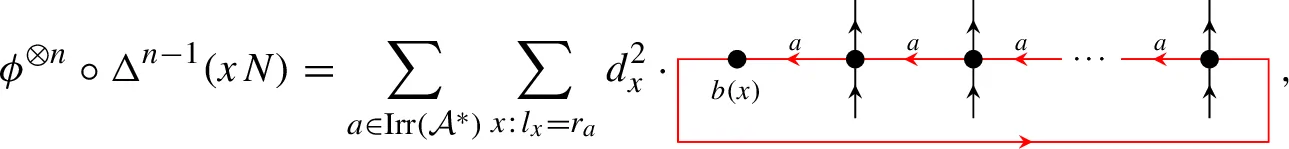

and, by Lemma 5.2, $\sum _{x:l_x = l_a} d_x^2 = \sum _{x: l_x = r_a} d_x^2$. Note as well that, as $d^2_x>0$ for all *x* (see again Lemma 5.2), *N* is invertible with inverse

**Figure:**
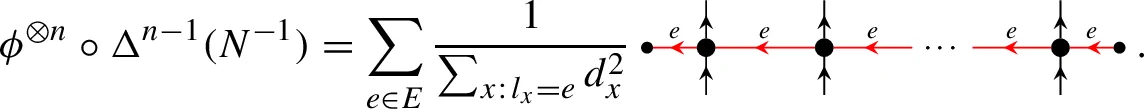

In fact, $\Lambda = N^{-1} L = LN^{-1}$. Therefore, in order to prove that Λ is a normalized left integral, we only have to prove that *L* is a left integral, and that $L^2 = N L$. Note here that Λ is of the form Eq. (5.29), and thus it is a q-trace. It is also non-degenerate as *g* is invertible and the normalization constant is non-zero.

Let us prove now that *L* is a left integral. Let us start with the following simple corollary of Lemma 5.1:

**Figure:**
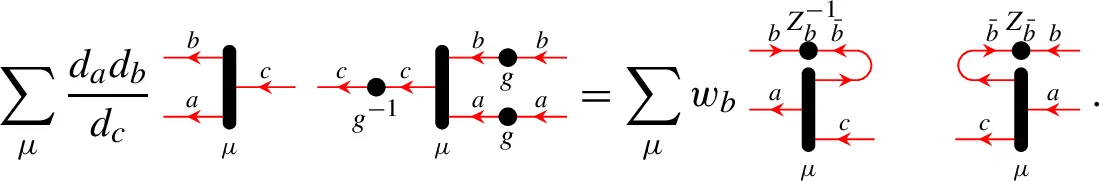

Let us rearrange this equation to obtain

**Figure:**
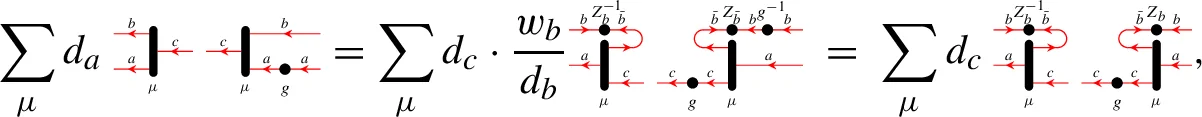

where in the second equality we have used that

**Figure:**
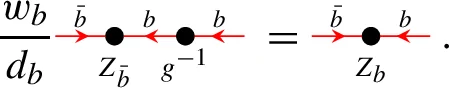

This implies that for all $x\in \mathcal {A}$,

**Figure:**
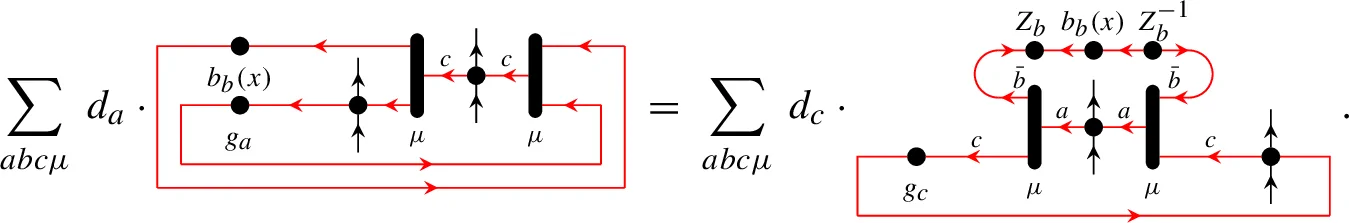

We can now use Eq. (4.8) to obtain

**Figure:**
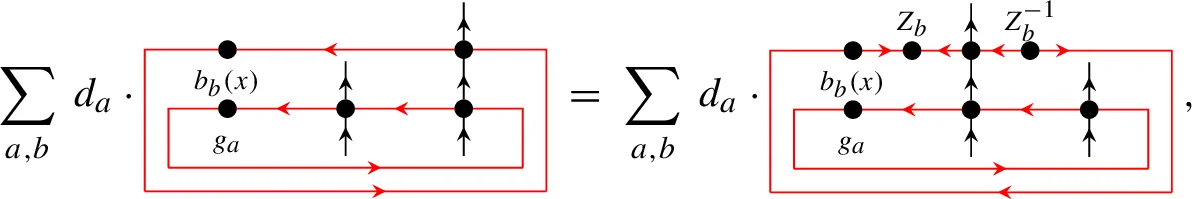

or equivalently, using that the representation ϕ is w.l.o.g. injective, that $(1\otimes x) \cdot \Delta (L) = (S(x)\otimes 1) \cdot \Delta (L)$, i.e. that *L* is a left integral. This integral is automatically non-degenerate (see Sect. 3.2) as $b_a(L) = d_a\psi _a(g)$ is invertible for all $a\in \textrm{Irr}(\mathcal {A}^*)$.

Let us now show that $L^2 = N L$, and thus that Λ is a normalized integral. The matrices $b_c(L^2)$ describing $L^2$ are of the form (see Eq. (4.10))

**Figure:**
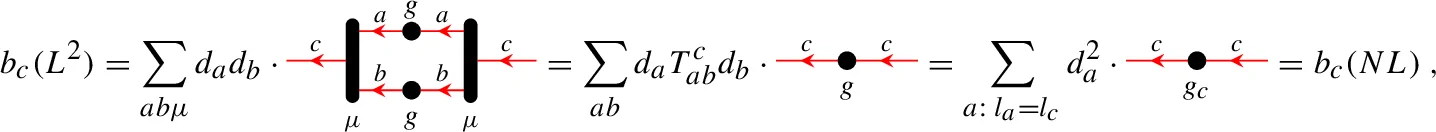

where in the second equality we have used the definition of $T_{ab}^c$, and in the third, the last point of Lemma 5.2. □

A few remarks are in place. First, it is known that every cosemisimple WHA is finite dimensional (see [Nik03] and Theorem 3.13 in [BNS99]), and thus the assumption on $\mathcal {A}$ being finite dimensional is redundant. Second, the normalized integral Λ provides a separability element $I\in \mathcal {A}\otimes \mathcal {A}$ via $I = (S^{-1}\otimes \textrm{Id}) \circ \Delta (\Lambda )$ for the algebra $\mathcal {A}$. Therefore $\mathcal {A}$ is separable, and in particular, it is semisimple, i.e. we have obtained Theorem 2.26. of [EGH+]. Finally, let us note that Λ is closely related to the canonical left integral $\hat{L}$ defined in [Nik03] (see also [BNS99] and [EGH+]). In [Nik03], the canonical left integral is defined as

$$\begin{aligned} \hat{L}(f) = \sum _{x\in B} x( f S^2(\delta _x)), \end{aligned}$$

where *B* is a basis of $\mathcal {A}$ and $\delta _x$ denotes the dual basis. In a finite dimensional cosemisimple weak Hopf algebra, this expression can be re-expressed using the matrix *b*(*x*):

$$\begin{aligned} \hat{L}(f) = \sum _{x\in B} \sum _{a\in \textrm{Irr}(\mathcal {A}^*)} \operatorname {Tr}\{ b_a(x) \cdot \psi _{a}(f) \cdot \psi _a ( S^2(\delta _x))\} \end{aligned}$$

Using now Eq. (5.26), we can also write

$$\begin{aligned} \hat{L}(f) = \sum _{a\in \textrm{Irr}(\mathcal {A}^*)} \sum _{x\in B} \operatorname {Tr}\{ b_a(x) \cdot \psi _{a}(f) \cdot \psi _{a}(g) \cdot \psi _a (\delta _x) \cdot \psi _a(g^{-1})\} \end{aligned}$$

As we sum over *x*, the matrix $\psi _a (\delta _x)$ takes all possible values in $\textrm{End}(W_a)$. Conversely, iterating over elements of $\bigoplus _{a\in \textrm{Irr}(\mathcal {A}^*)} \textrm{End}(V_a)$ defines a basis in $\mathcal {A}^*$ through the representation $\psi = \bigoplus _a \psi _a$. Let us fix a basis $\vert ai \rangle$ in each $W_a$ ($i=1, \ldots , \textrm{dim}(W_a)$), and choose the basis *B* such that for all *x*, there is $a\in \textrm{Irr}(\mathcal {A}^*)$ and $i,j=1, \ldots , \textrm{dim}(W_a)$ such that $\psi (\delta _x) = \vert ai \rangle \langle aj \vert$. It is easy to see that then $b_a(x) = \vert aj \rangle \langle ai \vert$. Therefore one can also write

$$\begin{aligned} \hat{L}(f)&= \sum _{a\in \textrm{Irr}(\mathcal {A}^*)} \sum _{ij} \operatorname {Tr}\{ \vert aj \rangle \langle ai \vert \cdot \psi _{a}(fg) \cdot \vert ai \rangle \langle aj \vert \cdot \psi _a(g^{-1})\}\\&= \sum _{a\in \textrm{Irr}(\mathcal {A}^*)} \sum _i \langle ai \vert \psi _{a}(fg) \vert ai \rangle \cdot \sum _j \langle aj \vert \psi _a(g^{-1}) \vert aj \rangle . \end{aligned}$$

Using the trace formula Eq. (5.27), $\operatorname {Tr}\psi _a(g^{-1}) = d_a \epsilon _{r_a}(1)$, $\hat{L}(f)$ can be further written as

$$\begin{aligned} \hat{L}(f) = \sum _{a\in \textrm{Irr}(\mathcal {A}^*)} d_a \epsilon _{r_a}(1) \cdot \operatorname {Tr}\{\psi _{a}(gf) \}, \end{aligned}$$

i.e. $\hat{L}$ is described by the boundary $\epsilon _{r_a}(1) \cdot d_a \psi _{a}(g)$, or equivalently, the MPO representation of $\hat{L}$ is given by

**Figure:**
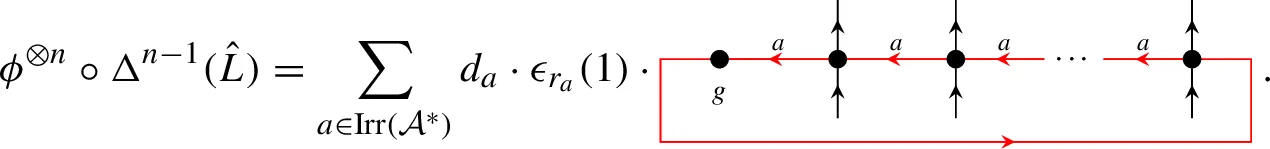

### Pivotal weak Hopf algebras

In certain WHAs one can define a special cocentral element using the integral Λ defined in the previous section. This element satisfies an equation reminiscent to the definition of an integral, but here, instead of the antipode, another operation appears. The WHAs where the construction works are called *pivotal* WHAs, and their defining property is that the antipode is special: the square of the antipode is an inner automorphism of the algebra realized by a *group-like* element (see the definition below). While such a structure seems to be restrictive, it is conjectured that any semisimple WHA is actually pivotal [EGH+].

#### Definition 5.5

(Group-like elements). Let $\mathcal {A}$ be a pre-bialgebra. $G\in \mathcal {A}$ is group-like if it is invertible and

$$\begin{aligned} \Delta (G) = (G\otimes G) \cdot \Delta (1) =\Delta (1) \cdot (G\otimes G). \end{aligned}$$

Group-like elements of a WHA $\mathcal {A}$ form a group: if $G,H\in \mathcal {A}$ are group-like, then *GH* is also group-like; $1\in \mathcal {A}$ is also group-like, and finally, if $G\in \mathcal {A}$, then $G^{-1}$ is group-like as well. In a WHA $S(G) = G^{-1}$ for any group-like *G* [BNS99].

$\mathcal {A}^*$ is a pre-bialgebra if and only if $\mathcal {A}$ is a pre-bialgebra; the unit of $\mathcal {A}^*$ is ϵ, and thus a linear functional $k\in \mathcal {A}^*$ is group-like if it is invertible and

$$\begin{aligned} \Delta (k) = (k\otimes k) \cdot \Delta (\epsilon ) =\Delta (\epsilon ) \cdot (k\otimes k). \end{aligned}$$

In a cosemisimple WHA $\mathcal {A}$, group-like elements of $\mathcal {A}^*$ have a nice characterization using the graphical representation of the fusion tensors. An element $k\in \mathcal {A}^*$ is group-like if and only if it is invertible and

**Figure:**
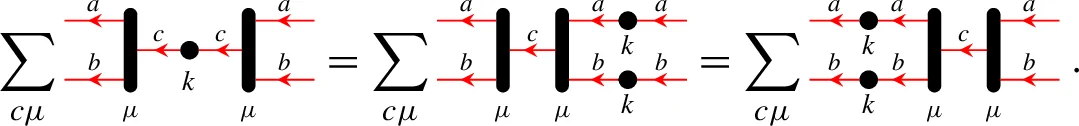

Using now the orthogonality relations of the fusion tensors, we conclude that *k* is group-like if and only if for all $a,b,c\in \textrm{Irr}(\mathcal {A}^*)$ and $\mu =1\dots N_{ab}^c$,

**Figure:**
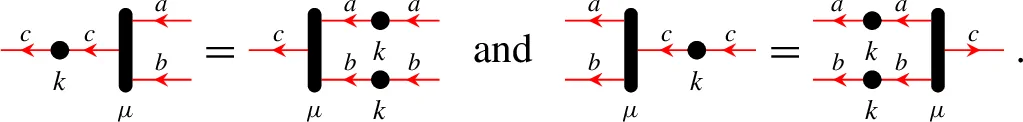

In the previous section (Lemma 5.2) we have seen a group-like element: the element $g^2\in \mathcal {A}^*$ is group-like, where *g* is defined by Eq. (5.25). Conjugation by this element describes $S^4$. A *pivotal* WHA is one where not only the fourth power, but also the square of the antipode is realized as a conjugation by a group-like element:

#### Definition 5.6

(Pivotal WHA). A WHA $\mathcal {A}$ is pivotal if there is a group-like element *G* such that for all $x\in \mathcal {A}$,

$$\begin{aligned} S^2(x) = G \cdot x \cdot G^{-1}. \end{aligned}$$

Such a group-like element *G* is called a pivotal element of $\mathcal {A}$.

If a WHA $\mathcal {A}$ is such that $\mathcal {A}^*$ is pivotal, then, as $S^2$ is an inner automorphism of $\mathcal {A}^*$ described by the linear functional *g* defined in Eq. (5.25), all pivotal elements *k* of $\mathcal {A}^*$ are of the form k=ξg for some $\xi \in \mathcal {A}^*$ central element. That is, there are numbers $\xi _a$ such that for all *a*,

**Figure:**
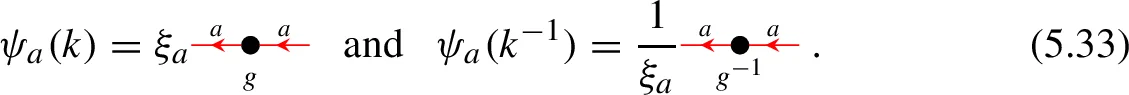

As $S(g) = g^{-1}$ and $S(k) = k^{-1}$, $\xi _{\bar{a}} = 1/\xi _a$. As $g^2$ is group-like (see Lemma 5.1), the numbers $\xi _a$ satisfy $\left( \xi _a \xi _b/\xi _c\right) ^2 = 1$ for all *a*, *b*, *c* such that $N_{ab}^c\ne 0$.

Let us consider a WHA $\mathcal {A}$ such that $\mathcal {A}^*$ is pivotal and let us fix the numbers $\xi _a$ such that the element $k\in \mathcal {A}^*$ defined by $k_a = \xi _a g_a$ is pivotal. In this case, as

**Figure:**
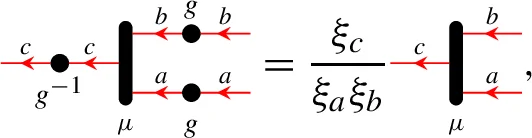

Lemma 5.1 takes the form

**Figure:**
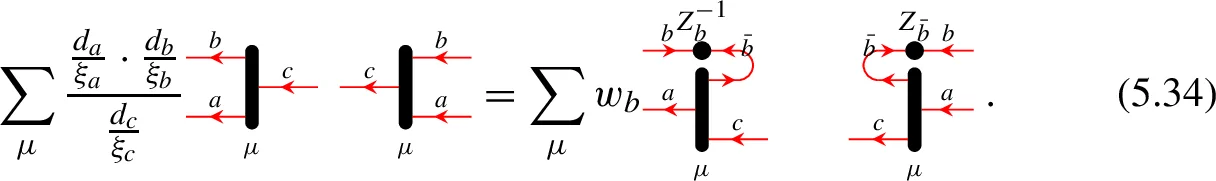

Let us now notice that the matrix $B_{ab}^c$ defined in Lemma 5.2 is proportional to the identity, more precisely, $B_{ab}^c = \xi _c/(\xi _a \xi _b) \cdot \textrm{Id}_{N_{ab}^c}$, and thus that $T_{ab}^c = N_{ab}^c\cdot \xi _c/(\xi _a \xi _b)$. We thus obtain that

5.35 $$\begin{aligned} \sum _b N_{ab}^c \cdot \frac{d_b}{\xi _b} = \delta _{l_a l_c} \cdot \frac{d_{\bar{a}}}{\xi _{\bar{a}}} \cdot \frac{d_c}{\xi _c}. \end{aligned}$$

With these statements we can now prove the following theorem:

#### Theorem 5.2

Let $\mathcal {A}$ be a finite dimensional weak Hopf algebra over $\mathbb {C}$ such that $\mathcal {A}^*$ is semisimple and pivotal with pivotal element $k\in \mathcal {A}^*$ such that $k_a = \xi _a g_a$. Then the element $\Omega \in \mathcal {A}$ defined by

**Figure:**
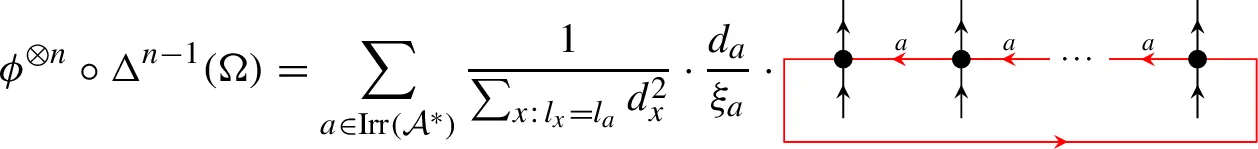

is non-degenerate and cocommutative, it is a projector, and there exists a linear map $T:\mathcal {A}\rightarrow \mathcal {A}$ such that

$$\begin{aligned} (1\otimes x) \cdot \Delta (\Omega ) = (T(x)\otimes 1) \cdot \Delta (\Omega ). \end{aligned}$$

This^9^ map *T* moreover satisfies

$$\begin{aligned} \Delta \circ T = (T\otimes k^{-1} \otimes T) \circ \Delta _{\textrm{op}}^2. \end{aligned}$$

The number $d_a/\xi _a$ is the *quantum dimension* of the irrep class *a*. This number depends on the concrete choice of *k* (equivalently, on the choice of the numbers $\xi _a$). Before proceeding to the proof, note that $\Omega = (g^{-1}\otimes \textrm{Id})\circ \Delta (\Lambda )$, where Λ is defined in Eq. (5.30). From this, using that *g* is group-like and the properties of Λ, simple (but tedious) algebraic calculations show all the desired properties. In particular, one can obtain that $T = (S\otimes g) \circ \Delta$. Instead of this algebraic calculation, below we provide a proof based on the graphical notation.

#### Proof of Theorem 5.2

By definition, Ω is cocommutative and as for all $a\in \textrm{Irr}(\mathcal {A}^*)$, $d_a\ne 0$, it is also non-degenerate. Let us now show that Ω is a projection. As in the proof of Theorem 5.1, note that Ω can be written as $N^{-1} \hat{\Omega } = \hat{\Omega } N^{-1}$, where $\hat{\Omega }$ is given by

5.36 $$\begin{aligned} \hat{\Omega } = \sum _{a\in \textrm{Irr}(\mathcal {A}^*)} \frac{d_a}{\xi _a} \cdot \tau _a, \end{aligned}$$

and *N* is given by Eq. (5.32). Then Ω is a projector if and only if $\hat{\Omega }$ satisfies $\hat{\Omega }^2 = N \hat{\Omega }$. Let us calculate $\hat{\Omega }^2$:

$$\begin{aligned} \hat{\Omega }^2 = \sum _{ab} \frac{d_ad_b}{\xi _a\xi _b} \cdot \tau _a\tau _b = \sum _{abc} \frac{d_ad_b}{\xi _a\xi _b} \cdot N_{ab}^c \tau _c =\sum _{ac} \frac{d_a}{\xi _a} \cdot \left( \sum _b N_{ab}^c \frac{d_b}{\xi _b}\right) \cdot \tau _c , \end{aligned}$$

and thus using Eq. (5.35), we obtain that

$$\begin{aligned} \hat{\Omega }^2 = \sum _{ac} \delta _{l_al_c} \frac{d_ad_{\bar{a}}}{\xi _a\xi _{\bar{a}}} \cdot \frac{d_c}{\xi _c}\cdot \tau _c = \sum _{c} \left( \sum _{a:l_a = l_c} d_a^2\right) \cdot \frac{d_c}{\xi _c}\cdot \tau _c = N \hat{\Omega }, \end{aligned}$$

where in the second equation we have used that $\xi _{\bar{a}} = \xi _a^{-1}$ and that $d_a = d_{\bar{a}}$. We have thus obtained that $\Omega = N^{-1} \hat{\Omega }$ is a projector.

Let us now check that $(1\otimes x) \cdot \Delta (\Omega ) = (T(x)\otimes 1) \cdot \Delta (\Omega )$ holds. Similar to the proof in Theorem 5.1, we start with Eq. (5.34):

**Figure:**
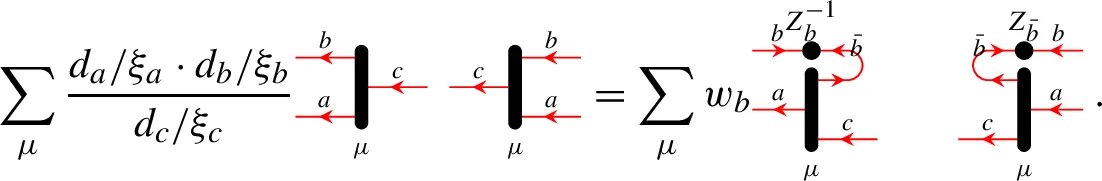

This implies that for all $x\in \mathcal {A}$,

**Figure:**
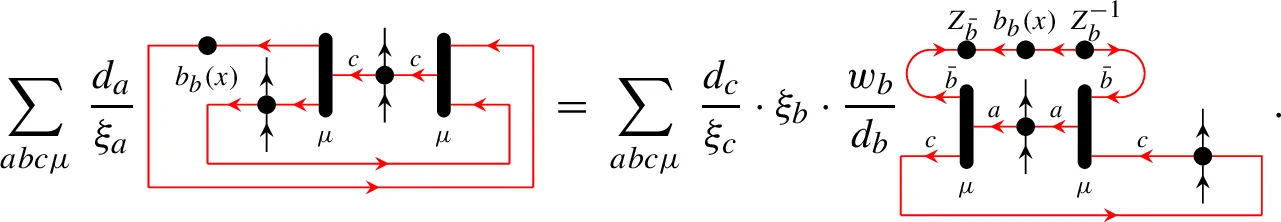

We can now use Eq. (4.8) to obtain

**Figure:**
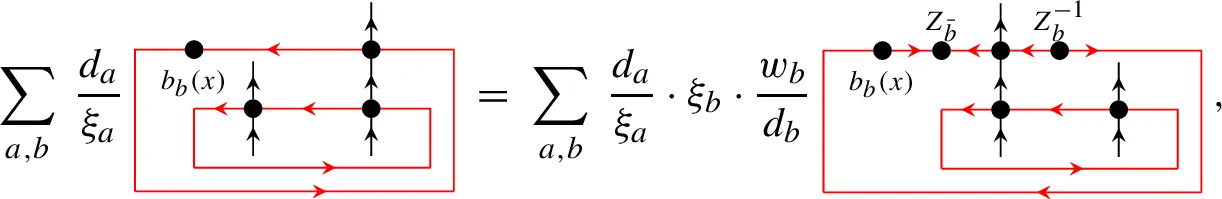

or $(1\otimes x) \cdot \Delta (\Omega ) = (T(x)\otimes 1) \cdot \Delta (\Omega )$, where *T* is defined by the formula

**Figure:**
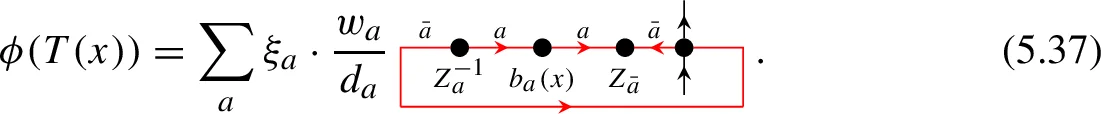

This means, in particular, that

**Figure:**
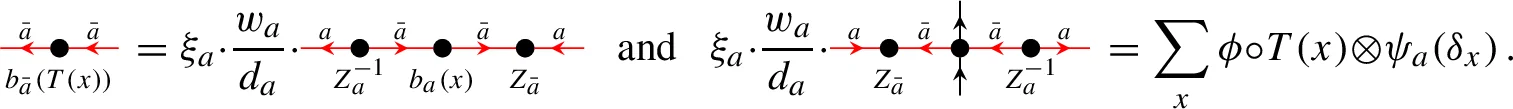

Let us now consider the MPO representation of $\Delta \circ T(x)$ and, using Eq. (5.25), observe that

**Figure:**
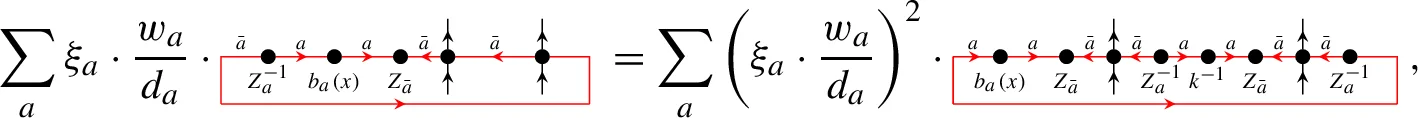

or, as ϕ is w.l.o.g. injective (notice that on the r.h.s. the arrows on the virtual index are reversed),

$$\begin{aligned} T\circ \Delta (x) = (T\otimes k^{-1} \otimes T) \circ \Delta _{\textrm{op}}^{2} (x). \end{aligned}$$

□

Note that the graphical representation of the action of *T*, Eq. (5.37), is very similar to that of *S*:

**Figure:**
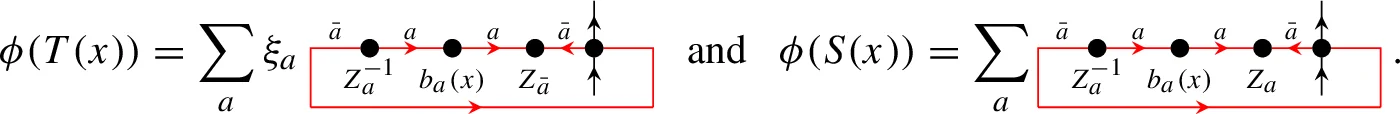

The difference between *T*(*x*) and *S*(*x*) is that in *T*(*x*) the matrix $\xi _a Z_{\bar{a}}$ appears instead of $Z_a$. As

**Figure:**
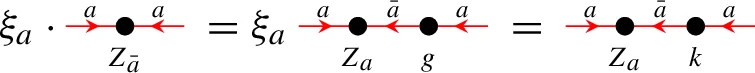

we directly obtain that $T(x) = (k^{-1} \otimes \textrm{Id})\circ \Delta (S(x))$, or, by using that *S* is an anti-homomorphism and that $k^{-1}\circ S = k$, that $T(x) = (S\otimes k) \circ \Delta (x)$. The inverse of this relation, as it can be see from Eq. (5.37), is $S(x) = (k^{-1}\otimes \textrm{Id})\circ \Delta \circ T(x)$, or $S(x) = (T\otimes k^{-1})\circ \Delta (x)$.

### Spherical weak Hopf algebras

In this section we define spherical weak Hopf algebras as pivotal weak Hopf algebras satisfying an additional property. Semisimple spherical weak Hopf algebras are such that their representation category is a spherical multi-fusion category. We specialize Theorem 5.2 to the case where $\mathcal {A}$ is not only pivotal, but also spherical.

#### Definition 5.7

(Spherical WHA). A pivotal WHA $\mathcal {A}$ is called spherical if the following two conditions hold. First, that for any irrep δ from the vacuum, $\epsilon (1_\delta ) \ne 0$, where $1_\delta$ is the projector onto the irrep sector δ. Second, it has a pivotal element *G* such that for all $\alpha \in \textrm{Irr}(\mathcal {A})$,

$$\begin{aligned} \frac{t_\alpha (G)}{\epsilon (1_{\lambda _\alpha })} = \frac{t_\alpha (G^{-1})}{\epsilon (1_{\rho _\alpha })}, \end{aligned}$$

where $t_\alpha$ is the irrep character of the irrep class α, $\lambda _\alpha$ is the unique irrep from the vacuum such that $t_{\lambda _\alpha } t_\alpha \ne 0$, and $\rho _\alpha$ is the unique irrep from the vacuum such that $t_\alpha t_{\rho _\alpha } \ne 0$. Such a pivotal element is called a spherical element of $\mathcal {A}$.

Let us now consider a WHA over $\mathbb {C}$ such that $\mathcal {A}^*$ is semisimple and spherical. Let *k* be a spherical element of $\mathcal {A}^*$ with

**Figure:**
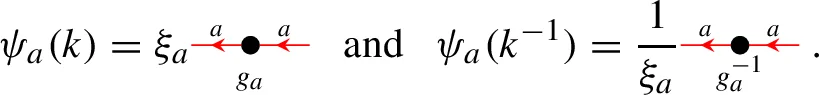

The traces of $\psi _{a}(k)$ and $\psi _a(k^{-1})$ can be then evaluated using Eq. (5.27) and (5.28):

$$\begin{aligned} \tau _a (k^{-1}) = \operatorname {Tr}\{\psi _a(k^{-1})\} = \frac{1}{\xi _a} \cdot \operatorname {Tr}\{\psi _a(g^{-1})\} = \frac{d_{a}}{\xi _a} \cdot \epsilon _{r_{a}} (1),\\ \tau _a(k ) = \operatorname {Tr}\{\psi _a (k)\} = \xi _a \cdot \operatorname {Tr}\{\psi _a(g)\} = \xi _a d_{a} \cdot \epsilon _{r_{\bar{a}}} (1). \end{aligned}$$

Sphericity of *k* implies that $\xi _a^2=1$ for all $a\in \textrm{Irr}(\mathcal {A}^*)$, i.e. that $\xi _a = \pm 1$. Together with the fact that $\xi _{\bar{a}} = 1/\xi _a$, this implies that $\xi _a = \xi _{\bar{a}}$.

Let us define now Ω as in Theorem 5.2, assuming that the pivotal element $k\in \mathcal {A}^*$ is spherical. Then Ω, using that $\xi _a = \xi _{\bar{a}}$, satisfies additionally that

$$\begin{aligned} (T\otimes k^{-1})\circ \Delta (\Omega ) = S(\Omega ) = \Omega . \end{aligned}$$

We have thus seen that

#### Theorem 5.3

Let $\mathcal {A}$ be a finite dimensional weak Hopf algebra over $\mathbb {C}$ such that $\mathcal {A}^*$ is semisimple and spherical with spherical element $k\in \mathcal {A}^*$ such that $\psi _a(k) = \xi _a \psi _{a}(g)$. Then the element $\Omega \in \mathcal {A}$ defined by

**Figure:**
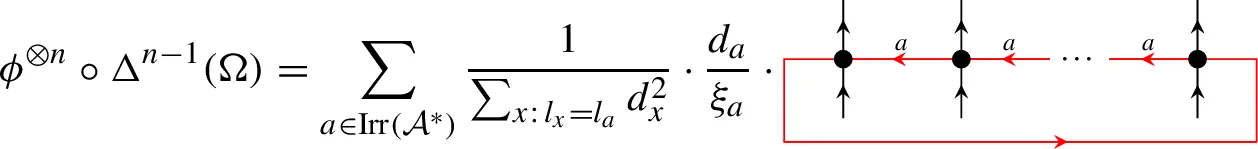

is non-degenerate and cocommutative, it is a projector, and there exists a linear map $T:\mathcal {A}\rightarrow \mathcal {A}$ such that

$$\begin{aligned} (1\otimes x) \cdot \Delta (\Omega ) = (T(x)\otimes 1) \cdot \Delta (\Omega ),\\ \Delta \circ T = (T\otimes k^{-1}\otimes T) \circ \Delta _{\textrm{op}}^2. \end{aligned}$$

Moreover, Ω satisfies

$$\begin{aligned} (T\otimes k^{-1})\circ \Delta (\Omega ) = S(\Omega ) = \Omega . \end{aligned}$$

### $C^*$-weak Hopf algebras

In this section we define $C^*$-weak Hopf algebras. We show how the ∗-operation acts on the representing MPOs. We then show that a $C^*$-weak Hopf algebra is spherical, and thus Theorem 5.3 applies.

#### Definition 5.8

($$C^*$$-*WHA).* A finite dimensional pre-bialgebra $$\mathcal {A}$$ over $$\mathbb {C}$$ is a ∗-pre-bialgebra if there is an anti-linear map $$*:\mathcal {A}\rightarrow \mathcal {A}$$ such that it is an involution ($$x^{**} = x$$), anti-homomorphism ($$(xy)^* = y^* x^*$$) and cohomomorphism ($$\Delta \circ * = (*\otimes *)\circ \Delta $$). It is a $$C^*$$-pre-bialgebra, if it is a ∗-pre-bialgebra and $$\mathcal {A}$$ has a faithful ∗-representation. A pre-bialgebra $$\mathcal {A}$$ is a ∗-WHA if it is both a ∗-pre-bialgebra and a WHA, and it is a $$C^*$$-WHA if it is both a $$C^*$$-pre-bialgebra and a WHA.

An equivalent characterization of a finite dimensional $C^*$-WHA is that it is a semisimple ∗-WHA such that it has a complete set of irreps $\phi _\alpha \; (\alpha \in \textrm{Irr}(\mathcal {A}))$ that are ∗-representations, i.e. $\phi _\alpha (x^*) = \phi _\alpha (x)^\dagger$. The dual of a $C^*$-WHA is also a $C^*$-WHA with ∗-operation defined by [BNS99]

5.38 $$\begin{aligned} f^*(x) = \overline{f\circ *\circ S(x)}, \end{aligned}$$

where $\overline{\lambda }$ denotes the complex conjugate of λ for all $\lambda \in \mathbb {C}$. As it is a $C^*$-WHA, $\mathcal {A}^*$ also possesses a complete set of ∗-representations. In this section, $\psi _a\; (a\in \textrm{Irr}(\mathcal {A}^*))$ will always denote such a complete set of irreps.

Let us now observe that the ∗-operation of $\mathcal {A}$ brings the character of $\psi _a$ into the character of $\psi _{\bar{a}}$:

#### Proposition 5.4

Let $\mathcal {A}$ be a $C^*$-WHA, and $\tau _a\in \mathcal {A}$ $(a\in \textrm{Irr}(\mathcal {A}^*))$ be the irrep characters of $\mathcal {A}^*$. Then $\tau _a^* = \tau _{\bar{a}}$ for all *a*.

#### Proof

Let us evaluate $\tau _a^*$ on a linear functional *f*:

$$\begin{aligned} \tau _a^*(f) = f(\tau _a^*) = \overline{f^* (S^{-1}(\tau _a))} = \overline{f^*(\tau _{\bar{a}})} = \overline{\tau _{\bar{a}} (f^*)}, \end{aligned}$$

where in the second equation we have used Eq. (5.38) with $x=S^{-1}(\tau _a)$. Finally note that, as $\psi _a$ is a ∗-representation,

$$\begin{aligned} \tau _{\bar{a}}(f^*) = \operatorname {Tr}\psi _{\bar{a}}(f^*) = \operatorname {Tr}\psi _{\bar{a}} (f)^\dagger = \overline{\operatorname {Tr}\psi _{\bar{a}}(f)} = \overline{\tau _{\bar{a}}(f)}, \end{aligned}$$

and thus for all $f\in \mathcal {A}^*$, $\tau _a^*(f) = \tau _{\bar{a}}(f)$. □

As the MPO representation of $\tau _a$ is the TI MPO defined by the injective block *a* of the MPO tensor representing the $C^*$-WHA, this proposition states that the injective MPO blocks are permuted under the ∗-operation:

**Figure:**
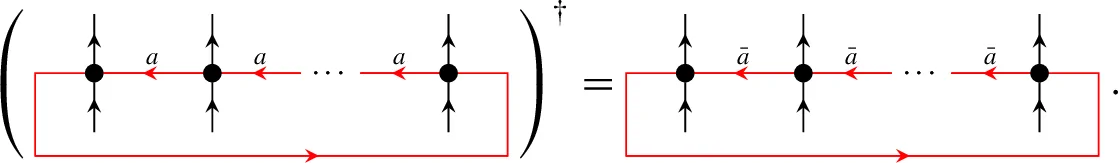

On the l.h.s. of this equation the dagger can be taken component-wise. More precisely, let us define a gray MPO tensor as

**Figure:**
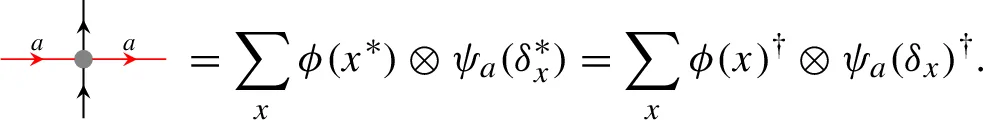

Notice that this MPO tensor is oriented in the opposite direction of the original (black) MPO tensor. By construction, the MPO defined by this MPO tensor is the Hermitian conjugate of the MPO describing $\tau _a$:

**Figure:**
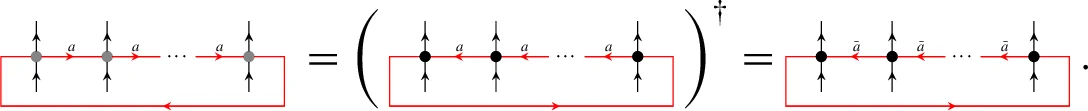

In fact, this equation holds with arbitrary boundary condition as well, i.e. the MPO representation of $x^*$ can be written as

**Figure:**
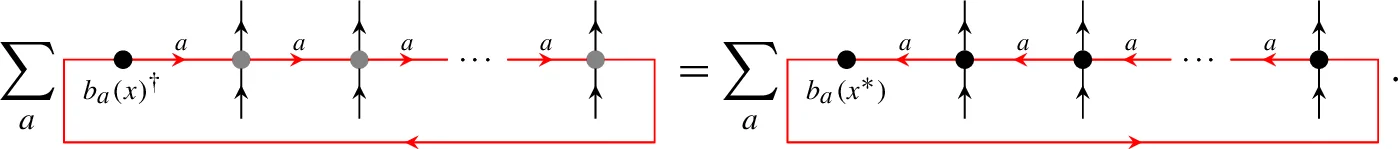

Let us finally note that the gray and the black tensors can be related to each other using Eq. (5.38). First note that there are two ways of expressing $y^*$:

$$\begin{aligned} \sum _x x \cdot \delta _x(y^*) = y^* = \sum _x x^* \cdot \overline{\delta _x(y)} = \sum _x x^* \cdot \delta _x^*\circ S^{-1}(y^*), \end{aligned}$$

where in the last equation we have used the fact that the conjugation can be expressed with the ∗ operation of $\mathcal {A}$ and $\mathcal {A}^*$ as follows:

$$\begin{aligned} \overline{f(x)} = \overline{f\circ * \circ S \circ S^{-1} \circ * (x)} = f^*\circ S^{-1}(x^*). \end{aligned}$$

As this equation holds for all *y*, we have obtained that

$$\begin{aligned} \sum _ x x^* \otimes \delta _x^* = \sum _x x \otimes S(\delta _x), \end{aligned}$$

or graphically,

**Figure:**
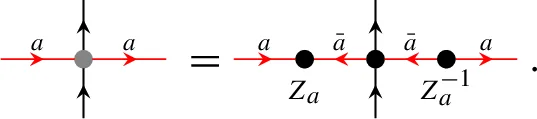

Note finally that then *b*(*x*) and $b(x^*)$ is also related:

**Figure:**
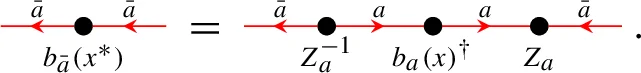

Let us now prove the well-known result that a $C^*$-WHA is pivotal, and in fact, spherical [EGH+, BNS99]:

#### Proposition 5.5

Let $\mathcal {A}$ be a $C^*$-WHA. Then $\mathcal {A}^*$ is also a $C^*$-WHA, it is spherical and the linear functional $g\in \mathcal {A}^*$ defined in Eq. (5.25) is a spherical element of it that is also positive.

#### Proof

As mentioned above, we can fix the irrep representatives $\psi _a$ of $\mathcal {A}^*$ to be ∗ representations. This implies, as the ∗ operation of $\mathcal {A}^*$ is a cohomomorphism, $\Delta = (* \otimes *) \circ \Delta \circ *$, that

**Figure:**
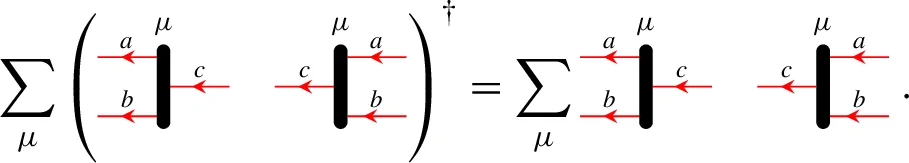

This equation implies that for all $a,b,c\in \textrm{Irr}(\mathcal {A}^*)$ there exist a matrix $A_{ab}^c$ such that the dagger of the fusion tensor, denoted by gray tensors, can be expressed as

**Figure:**
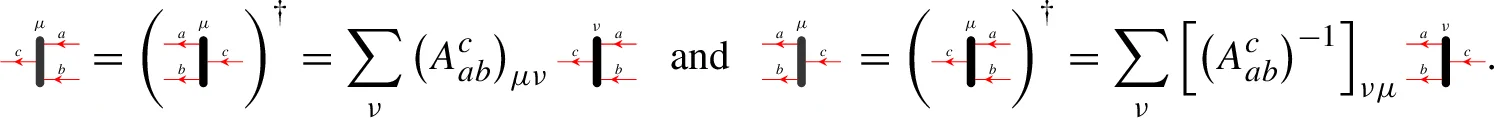

These matrices $A_{ab}^c$ are in fact positive, as using the orthogonality relations, they are in the form

**Figure:**
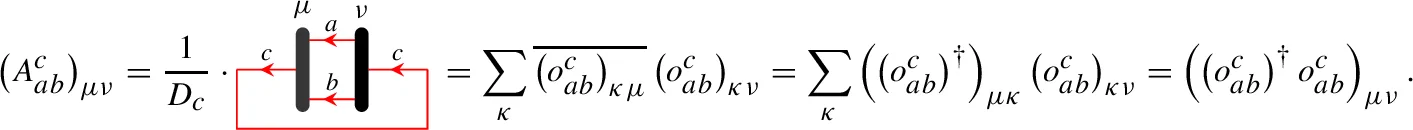

Therefore changing the fusion tensors to

$$\begin{aligned} \hat{V}_{ab}^{c\mu } = \sum _{\kappa } \left( \left( o_{ab}^c\right) ^{-1}\right) _{\mu \nu } V_{ab}^{c\nu } \quad \text {and} \quad \left( \hat{W}_{ab}^c\right) _{\mu \nu } = \sum _{\kappa } \left( o_{ab}^c\right) _{\mu \kappa } \left( V_{ab}^c\right) _{\kappa \nu }, \end{aligned}$$

we obtain $\left( \hat{V}_{ab}^{c\mu }\right) ^\dagger = \hat{W}_{ab}^{c\mu }$. That is, w.l.o.g. we can assume that the fusion tensors are the hermitian conjugates of each other,

**Figure:**
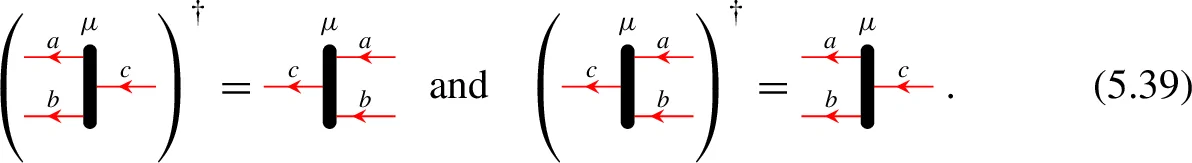

Let us now investigate what restrictions the $C^*$ structure of $\mathcal {A}^*$ imposes on the representation of the unit of the algebra. The unit $1\in \mathcal {A}$ is invariant under both the ∗-operation as well as under the action of the antipode, therefore

$$\begin{aligned} 1(f) = 1^*(f) = \overline{1(S(f^*))} = \overline{1(f^*)}. \end{aligned}$$

Let us write  and , then this equation reads

$$\begin{aligned} \sum _{e\in E} \langle v_e \vert \psi _e (f) \vert w_e \rangle = \sum _{e\in E} \overline{\langle v_e \vert \psi _e (f^*) \vert w_e \rangle } = \sum _{e\in E} \langle w_e \vert \psi _e (f^*)^\dagger \vert v_e \rangle = \sum _{e\in E} \langle w_e \vert \psi _e (f) \vert v_e \rangle , \end{aligned}$$

where in the last equation we have used that $\psi _e$ is a ∗-representation. As this equation holds for all *f*, we have obtained that

**Figure:**
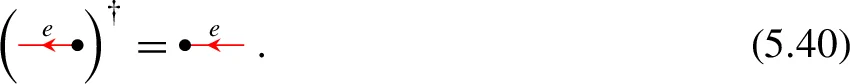

Using that the fusion tensors and the vectors representing the unit are self-adjoint, we can now prove that the matrices $Z_a$ can be chosen such that $\left( Z_{\bar{a}}^{-1}\right) ^\dagger = Z_a$. To see that, let us first take the dagger of Eq. (5.24) using Eq. (5.39) and (5.40):

**Figure:**
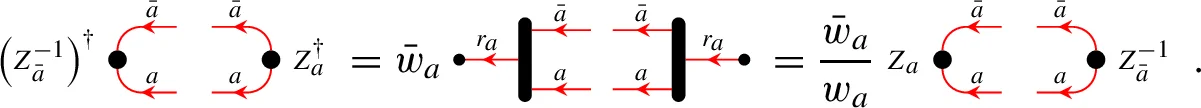

This implies that there is $\lambda _a\in \mathbb {C}$ such that $\left( Z_{\bar{a}}^{-1}\right) ^\dagger = \lambda _a Z_a$ and $Z_a^\dagger = \bar{w}_a/(w_a \lambda _a) \cdot Z_{\bar{a}}^{-1}$. Changing *a* to $\bar{a}$ in the first equation, we obtain that $\left( Z_{a}^{-1}\right) ^\dagger = \lambda _{\bar{a}} Z_{\bar{a}}$, or, after rearranging, $\left( Z_{\bar{a}}^{-1}\right) ^\dagger = \overline{\lambda _{\bar{a}}} Z_a$, and thus $\lambda _a = \overline{\lambda _{\bar{a}}}$. This implies that if *a* is such that $\bar{a}\ne a$, then $\lambda _a = \mu _a \overline{\mu _{\bar{a}}}$ can be solved, e.g. by $\mu _a = \lambda _a$ and $\mu _{\bar{a}}=1$. With this choice, $\lambda _{\bar{a}} = \mu _{\bar{a}} \overline{\mu _{a}}$ also holds. If $\bar{a} = a$, one has to be more careful. In this case, $\lambda _a = \overline{\lambda _{\bar{a}}}$ implies that $\lambda _a$ is real. To solve $\lambda _a = \mu _a \overline{\mu _{\bar{a}}} = |\mu _a|^2$, we have to show that $\lambda _a$ is not only real, but also positive. To show that, note that $\left( Z_{a}^{-1}\right) ^\dagger = \lambda _a Z_a$, or $\textrm{Id}_a = \lambda _a Z_a Z_a^\dagger$. As both $\textrm{Id}_a$, and $Z_a Z_a^\dagger$ are positive, this implies that $\lambda _a$ is positive, and thus $\lambda _a = \mu _a \overline{\mu _{\bar{a}}}$ can be solved as well. Let $\hat{Z}_a = \mu _a Z_a$, then $\left( \hat{Z}_{\bar{a}}^{-1}\right) ^\dagger = \hat{Z}_a$. This means that we can assume w.l.o.g. that $\left( Z_{\bar{a}}^{-1}\right) ^\dagger = Z_a$.

Let us show now that the linear functional $g\in \mathcal {A}^*$ defined in Eq. (5.25) is a pivotal element that is positive and spherical. First, the equation $\left( Z_{\bar{a}}^{-1}\right) ^\dagger = Z_a$ implies that both $w_a$ and $\psi _a(g)$ are positive, and thus *g* is positive as well. Let us show now that the matrix $B_{ab}^c$ defined in Lemma 5.2 is positive as well. This matrix can be obtained by

**Figure:**
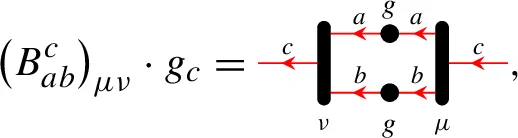

i.e. it is a positive matrix. As $B_{ab}^c$ is positive and it squares to the identity, it is the identity, and thus *g* is a (positive) pivotal element. Note that it is spherical as well, because

$$\begin{aligned} \operatorname {Tr}\psi _a(g) = d_a \cdot \epsilon _{r_a}(1) = d_a \cdot \epsilon _{r_{\bar{a}}}(1) = \operatorname {Tr}\psi _a(g^{-1}). \end{aligned}$$

We have thus derived the well-known result that in a $C^*$-WHA there is a positive spherical element. □

Let us now use this positive pivotal element in the construction in Theorem 5.3. With this choice, the resulting element Ω has MPO representation

**Figure:**
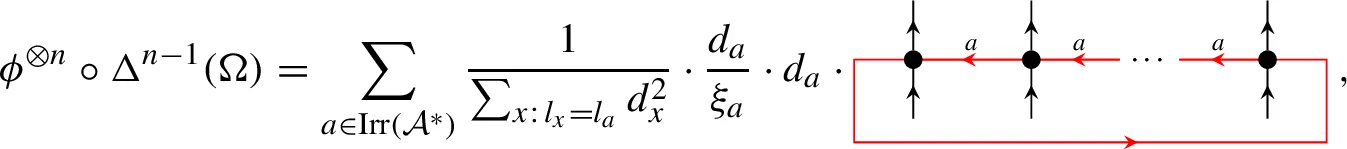

where all $d_a$ are positive and $d_a = d_{\bar{a}}$. As $d_a$ are positive, Ω is a positive linear functional on $\mathcal {A}^*$: for any positive *f* the representing matrix $\psi _a(f)$ is positive, and thus $\Omega (f) = \sum _a d_a \cdot \operatorname {Tr}\psi _a(f)\ge 0$. Similarly, $d_a = d_{\bar{a}}$ implies that $\Omega ^* = \Omega$, and therefore, as Ω is also a projector, it is a positive element of $\mathcal {A}$.

We have thus seen that the following theorem holds:

#### Theorem 5.4

Let $\mathcal {A}$ be a finite dimensional $C^*$-weak Hopf algebra over $\mathbb {C}$. Then $\mathcal {A}^*$ is semisimple and spherical with positive spherical element $g\in \mathcal {A}^*$ defined in Eq. (5.25). Then the element $\Omega \in \mathcal {A}$ defined by

**Figure:**
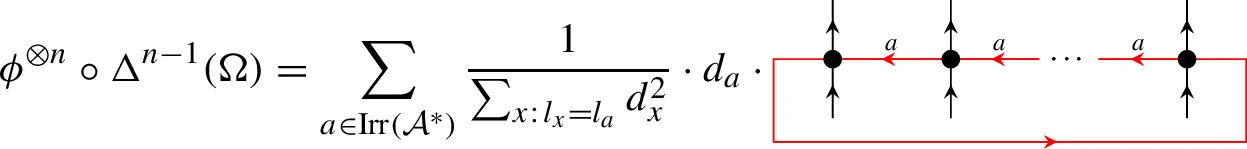

is a positive non-degenerate trace-like linear functional on $\mathcal {A}^*$, it is an orthogonal projector, and there exists a linear map $T:\mathcal {A}\rightarrow \mathcal {A}$ such that

$$\begin{aligned} (1\otimes x) \cdot \Delta (\Omega ) = (T(x)\otimes 1) \cdot \Delta (\Omega ),\\ \Delta \circ T = (T\otimes g\otimes T) \circ \Delta _{\textrm{op}}^2. \end{aligned}$$

Moreover, Ω satisfies

$$\begin{aligned} (T\otimes g^{-1})\circ \Delta (\Omega ) = S(\Omega ) = \Omega . \end{aligned}$$

Let us note that the (positive) numbers $d_a$ satisfy (see Lemma 5.2) $\sum _b N_{ab}^c d_b = \delta _{l_al_c} d_a \cdot d_c$. In particular, if *E* consists of a single irrep *e*, then $\delta _{l_al_c}$ is never zero and thus $d_b$ defines a positive eigenvector for the matrix $N_a$ defined by $(N_a)_b^c = N_{ab}^c$. As $N_{a}$ is a non-negative matrix, this implies that the corresponding eigenvalue, $d_{a}$, is the spectral radius of $N_{a}$. This number is also called the Perron-Froebenius dimension of $a\in \textrm{Irr}(\mathcal {A}^*)$.

Note finally that if $\mathcal {A}$ is a $C^*$ weak Hopf algebra, then $\mathcal {A}^*$ is also a $C^*$ weak Hopf algebra. This allows us to define an element $\omega \in \mathcal {A}^*$ that is a positive non-degenerate trace-like linear functional on $\mathcal {A}$. Using this ω then one can define a scalar product on $\mathcal {A}$ by

$$\begin{aligned} \langle x \vert y \rangle = \omega (x^* y). \end{aligned}$$

### $C^*$-Hopf algebras

In this section we further particularize the results obtained in the previous sections to $C^*$-Hopf algebras. As a $C^*$-Hopf algebra is a special $C^*$-WHA, Theorem 5.4 applies. We will show that the element Ω obtained from this theorem is in fact the Haar integral of the Hopf algebra.

Before stating the definition of a $C^*$ Hopf algebra, note that for a WHA $\mathcal {A}$ the following statements are all equivalent [BNS99]:

- $$\Delta _{\mathcal {A}}(1) = 1\otimes 1$$,
- $$\Delta _{\mathcal {A}^*}(\epsilon ) = \epsilon \otimes \epsilon $$,
- $$\sum _{(x)} S(x_{(1)}) x_{(2)} = \epsilon (x) 1$$,
- $$\sum _{(x)} x_{(1)} S(x_{(2)}) = \epsilon (x) 1$$.

Keeping this equivalence in mind, one can define Hopf algebras and $\mathcal {C}^*$ Hopf algebras as follows:

#### Definition 5.9

(Hopf algebra and $C^*$ *Hopf algebra).* A Hopf algebra is a weak Hopf algebra such that $\Delta (1) = 1\otimes 1$. A $C^*$ Hopf algebra is a $C^*$ weak Hopf algebra such that $\Delta (1) = 1\otimes 1$.

Any $C^*$-Hopf algebra $\mathcal {A}$ is semisimple, and thus, due to the Larson-Radford theorem, $S^2=\textrm{Id}$. This implies that the (unique) positive pivotal element of $\mathcal {A}^*$ is ϵ, and thus the numbers $\operatorname {Tr}(\psi _a(g)) = d_a$ appearing in Theorem 5.5 are in fact $d_a = \operatorname {Tr}(\psi _a(\epsilon )) = D_a$, the dimension of the irrep *a*. Moreover, Ω in Theorem 5.4 and Λ in Theorem 5.1 coincide. In particular, as in a $C^*$ Hopf algebra the unique normalized integral is the Haar integral (Larson-Sweedler theorem), Ω is the Haar integral of $\mathcal {A}$. We have thus seen that

#### Theorem 5.5

Let $\mathcal {A}$ be a finite dimensional $C^*$ Hopf algebra over $\mathbb {C}$. Then $\mathcal {A}^*$ is semisimple and spherical and its positive spherical element is $\epsilon \in \mathcal {A}^*$. The element $\Omega \in \mathcal {A}$ defined by

**Figure:**
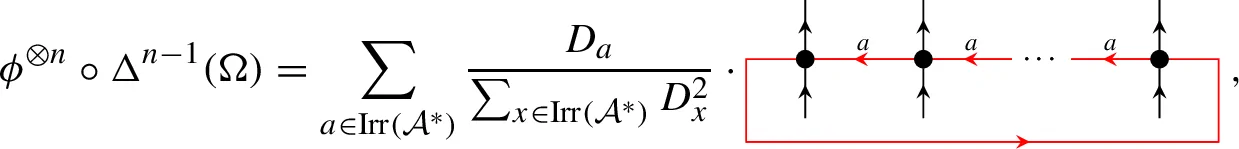

where $D_a$ is the dimension of the irrep *a*, is the Haar integral of $\mathcal {A}$.

In particular, as g=ϵ, the map T=S and the equations

$$\begin{aligned} (1\otimes x) \cdot \Delta (\Omega ) = (T(x)\otimes 1) \cdot \Delta (\Omega ),\\ \Delta \circ T = (T\otimes g\otimes T) \circ \Delta _{\textrm{op}}^2. \end{aligned}$$

simplify to the definition of the integral and to the fact that *S* is an anti-cohomomorphism:

$$\begin{aligned} (1\otimes x) \cdot \Delta (\Omega ) = (S(x)\otimes 1) \cdot \Delta (\Omega ),\\ \Delta \circ S = (S\otimes S) \circ \Delta _{\textrm{op}}. \end{aligned}$$

## Pulling-Through Algebras

In the previous sections we have seen (Theorem 5.2, and its variants Theorem 5.3 and Theorem 5.4) that in a semisimple pivotal WHA over $\mathbb {C}$ there is a special non-degenerate cocentral element Ω that behaves almost like an integral. In this section we abstract this notion and investigate the structure such an element provides, independent from semisimplicity or weak Hopf algebras. The main reason behind this abstraction is that this is exactly the structure we need to use in order to define a PEPS with symmetries, see Sect. 7.

### Definition 6.1

(Pulling-through algebra). A finite dimensional pre-bialgebra over $\mathbb {C}$ is called a pulling-through algebra if there is a cocentral, non-degenerate element $\Omega \in \mathcal {A}$, a linear map $T:\mathcal {A}\rightarrow \mathcal {A}$ and a group-like linear functional $g\in \mathcal {A}^*$ such that for all $x\in \mathcal {A}$,

6.1 $$\begin{aligned}&(1\otimes x) \cdot \Delta (\Omega ) = (T(x) \otimes 1) \cdot \Delta (\Omega ), \end{aligned}$$

6.2 $$\begin{aligned}&\Delta \circ T = (T\otimes g \otimes T)\circ \Delta _{\textrm{op}}^2 . \end{aligned}$$

Equation 6.1 will be called *pulling-through* equation. The rationale behind the name will become clear when we give a tensor network description in Eq. (6.4) below. In a pulling-through algebra, Ω uniquely determines *T*: if there was another map $\hat{T}:\mathcal {A}\rightarrow \mathcal {A}$ also satisfying Eq. (6.1), then

$$\begin{aligned} (T(x) \otimes 1) \cdot \Delta (\Omega ) = (1\otimes x) \cdot \Delta (\Omega ) = (\hat{T}(x) \otimes 1) \cdot \Delta (\Omega ), \end{aligned}$$

and thus by non-degeneracy of Ω, $\hat{T}(x) = T(x)$. Using that $\Delta (\Omega ) = \Delta _{\textrm{op}}(\Omega )$, one can show that *T* is an involution:

$$\begin{aligned} (1\otimes x) \cdot \Delta (\Omega )&= (T(x) \otimes 1) \cdot \Delta (\Omega ) = (T(x) \otimes 1) \cdot \Delta _{\textrm{op}}(\Omega )\\&= (1 \otimes T^2(x))\cdot \Delta _{\textrm{op}}(\Omega ) = (1 \otimes T^2(x))\cdot \Delta (\Omega ), \end{aligned}$$

where the third equation is the pulling-through equation for y=T(x). As above, due to non-degeneracy of Ω, $x= T^2(x)$, i.e. *T* is an involution. In particular, *T* is invertible, and thus there can be at most one linear functional $g\in \mathcal {A}^*$ satisfying Eq. (6.2). Let us show now that *T* is an anti-homomorphism by using the pulling-through equation Eq. (6.1) twice:

$$\begin{aligned} (T(xy)\otimes 1)\Delta (\Omega ) = (1\otimes xy)\Delta (\Omega ) = (T(y)\otimes x)\Delta (\Omega ) = (T(y) T(x)\otimes 1)\Delta (\Omega ), \end{aligned}$$

and thus non-degeneracy of Ω implies that T(xy)=T(y)·T(x).

Let us note that in a pre-bialgebra $\mathcal {A}$ there might be several different elements Ω that make it a pulling-through algebra. For example, it is easy to check that given an element $\Omega \in \mathcal {A}$ defining a pulling-through structure on $\mathcal {A}$ and any central group-like element $c\in \mathcal {A}^*$ (if there is any), the element $\hat{\Omega } = (\textrm{Id}\otimes c) \circ \Delta (\Omega )$ defines another pulling-though structure. This is the case if in the construction of Theorem 5.2 we use two different pivotal elements *g* and $\hat{g}$ to arrive at the cocentral elements Ω and $\hat{\Omega }$, respectively.

Let $\mathcal {A}$ be a pulling-through algebra. As $\mathcal {A}$ is a pre-bialgebra, it has MPO representations: given any representation ϕ of $\mathcal {A}$ and an injective representation ψ of $\mathcal {A}^*$ on a vector space *W*, let us define an MPO tensor by

**Figure:**
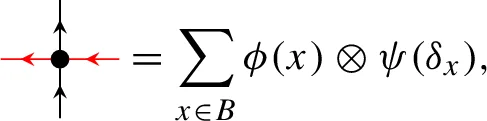

where *B* is a basis of $\mathcal {A}$, and $\delta _x$ denotes the dual basis (i.e. $\delta _x(y) = \delta _{xy}$). Given this MPO tensor, for all $x\in \mathcal {A}$ there is a matrix $b(x)\in \textrm{End}(W)$ such that

**Figure:**
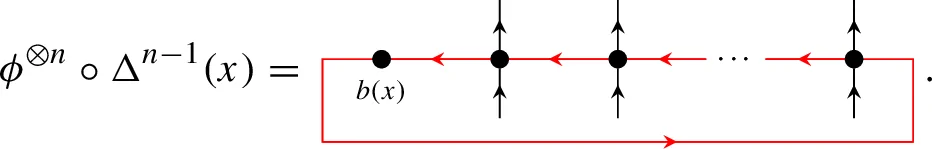

Notice that in this section we do not assume semisimplicity of $\mathcal {A}^*$. The MPOs, nevertheless, are still multiplicative,

**Figure:**
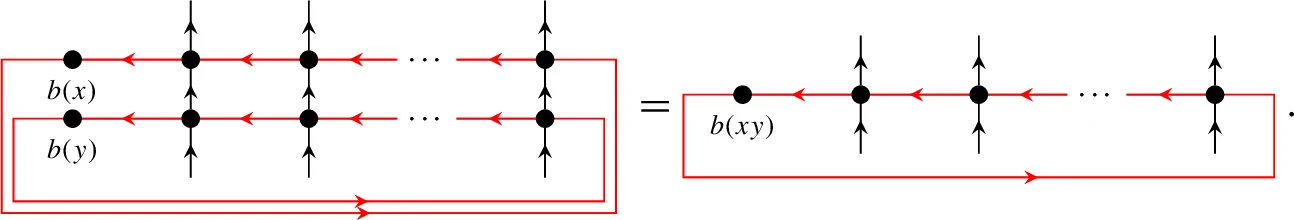

Let us introduce a new MPO tensor, denoted by white dot, as

**Figure:**
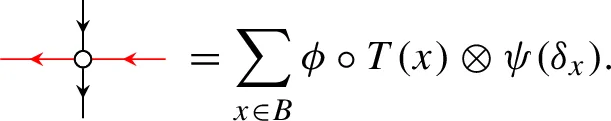

Note that as *T* is an anti-homomorphism, this tensor is flipped upside down w.r.t. the tensor denoted by full dot, i.e. the input index of ϕ is on the top of the tensor, not on the bottom. *T*(*x*) can be expressed both with the original tensor and this new tensor:

**Figure:**
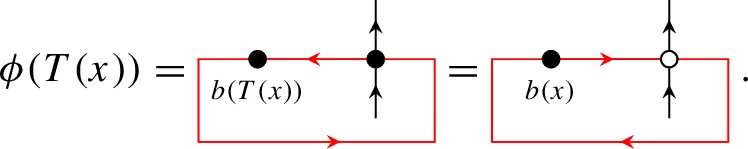

Note that the orientation of the red lines in the second MPO is the opposite of the orientation of the red lines in the first MPO. This is because to be able to compare the tensor networks on the two sides of the equation, the white tensor had to be rotated.

With the help of this new tensor, the identity $\sum _{(xy)} (xy)_{(1)} \otimes T((xy)_{(2)}) = \sum _{(x)(y)} x_{(1)} y_{(1)} \otimes T(y_{(2)}) T(x_{(2)})$ has a particularly nice MPO representation:

**Figure:**
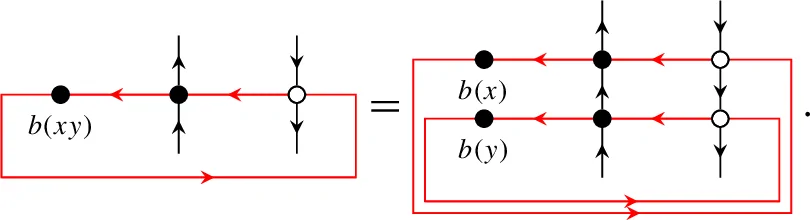

Let small green dot denote the (representation of the) group-like element *g*; then the tensor network representation of the identity $\Delta \circ T(x) = (T\otimes g\otimes T) \circ \Delta ^2_{op}(x)$ is

**Figure:**
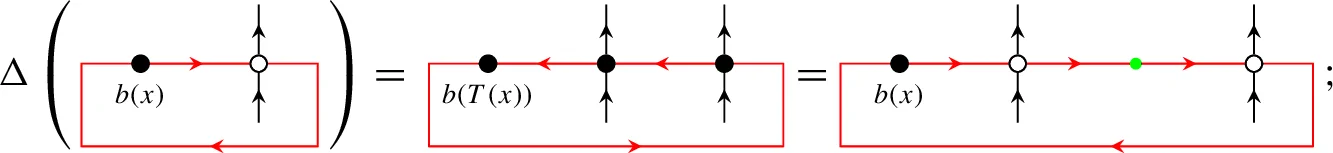

notice here that the third MPO is oriented in the opposite direction as the second one. This is due to the fact that it is the MPO corresponding to $\Delta _{\textrm{op}}$, and not to Δ. This equation describes the coproduct of the white tensor:

**Figure:**
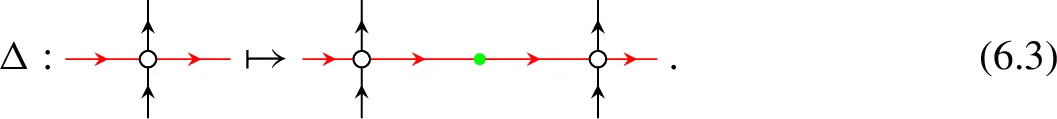

We will denote the boundary corresponding to the pulling-through element Ω by a small blue dot without label:

**Figure:**
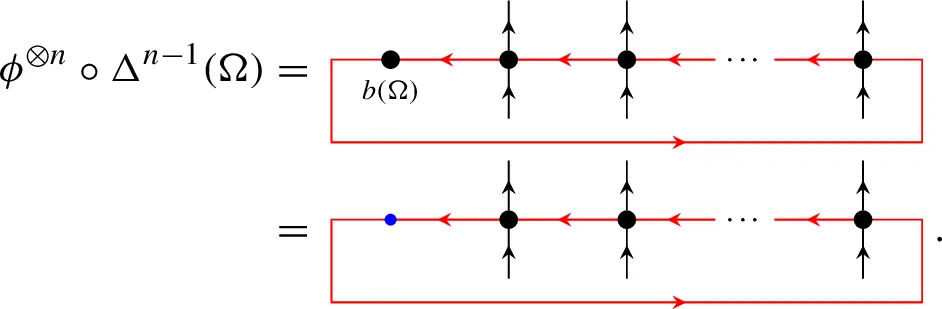

As Ω is co-central, the corresponding MPO is translation invariant, that is,

**Figure:**
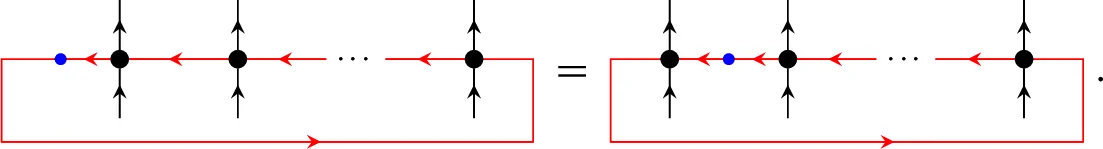

Let us now explain how these MPO representations will be used when depicting two-dimensional tensor networks in the next section. Keeping in mind that the orientation of each MPO tensor has to be kept, we can draw MPOs in arbitrary form such as on a circle. For example, the fact that Ω is cocentral can be represented by the equation

**Figure:**
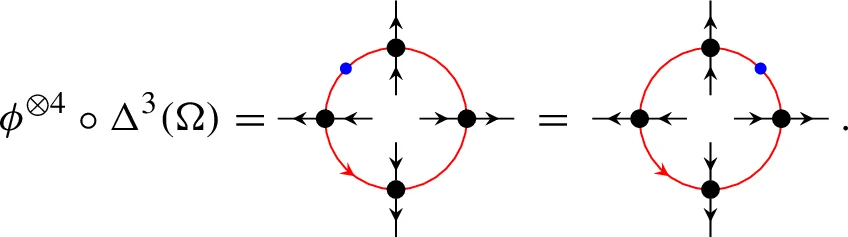

The pulling through equation $(1\otimes x) \cdot \Delta (\Omega ) = (T(x) \otimes 1) \cdot \Delta (\Omega )$ is represented by the following tensor network notation:

**Figure:**
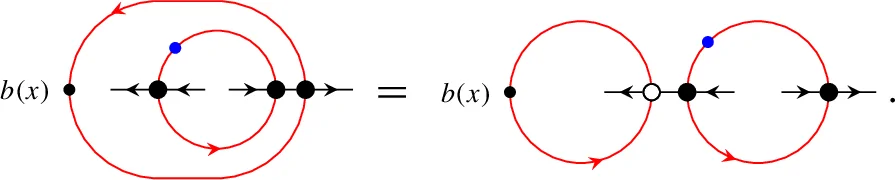

As this equation holds for all $x\in \mathcal {A}^*$, it also holds with open indices:

**Figure:**
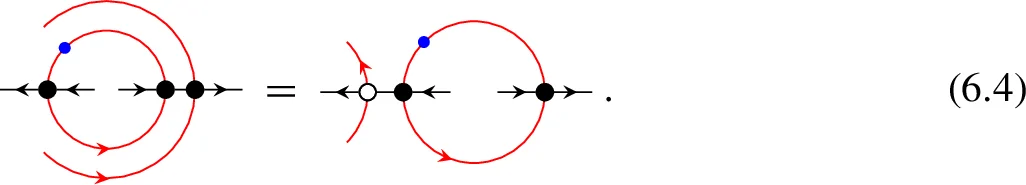

Applying *T* on the left tensor component of this equation, we obtain

**Figure:**
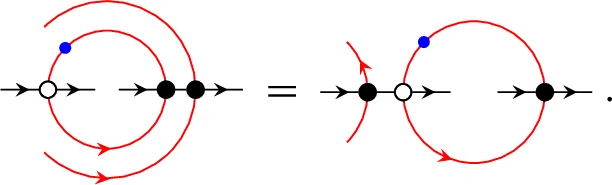

This equation is in fact why $(1\otimes x) \cdot \Delta (\Omega ) = (T(x) \otimes 1) \cdot \Delta (\Omega )$ is called pulling-through equation: it states that the MPO described by the black MPO tensors can be pulled through the circular MPO describing $(T\otimes \textrm{Id})\circ \Delta (\Omega )$.

To familiarize ourselves with the notation, we derive some equations that we will use in the following section. Taking different coproducts of Eq. (6.4), and using Eq. (6.3), we arrive at, for example,

**Figure:**
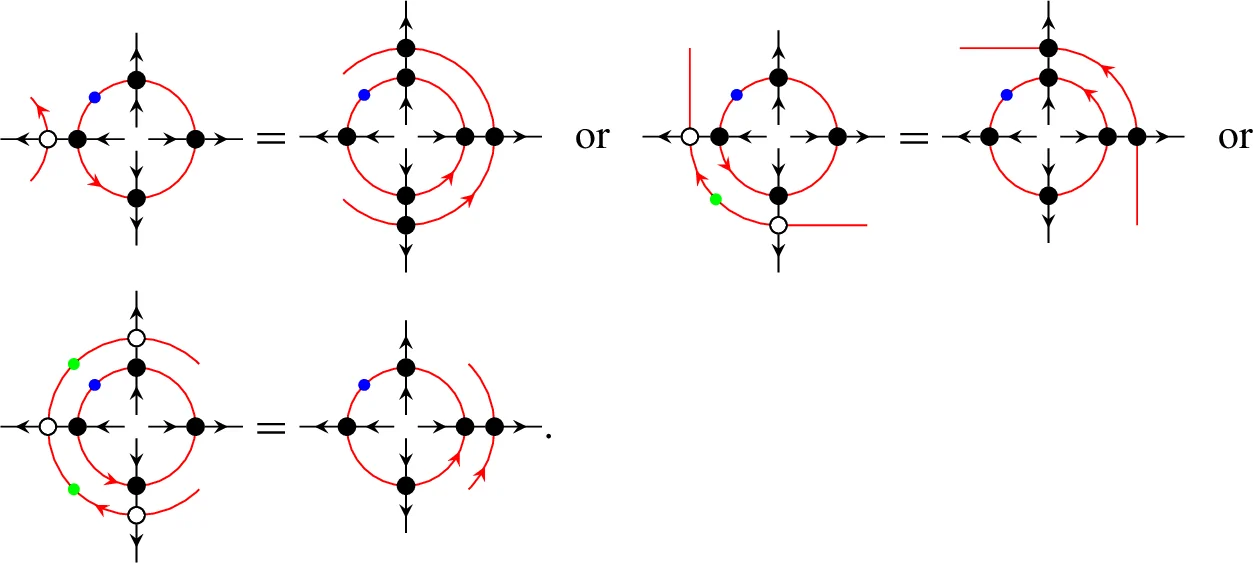

Applying *T* on some of the tensor components reverses the orientation of the lines corresponding to the physical indices of those tensor components, and changes the black tensors into white ones and vice versa, that is, for example, the following holds:

**Figure:**
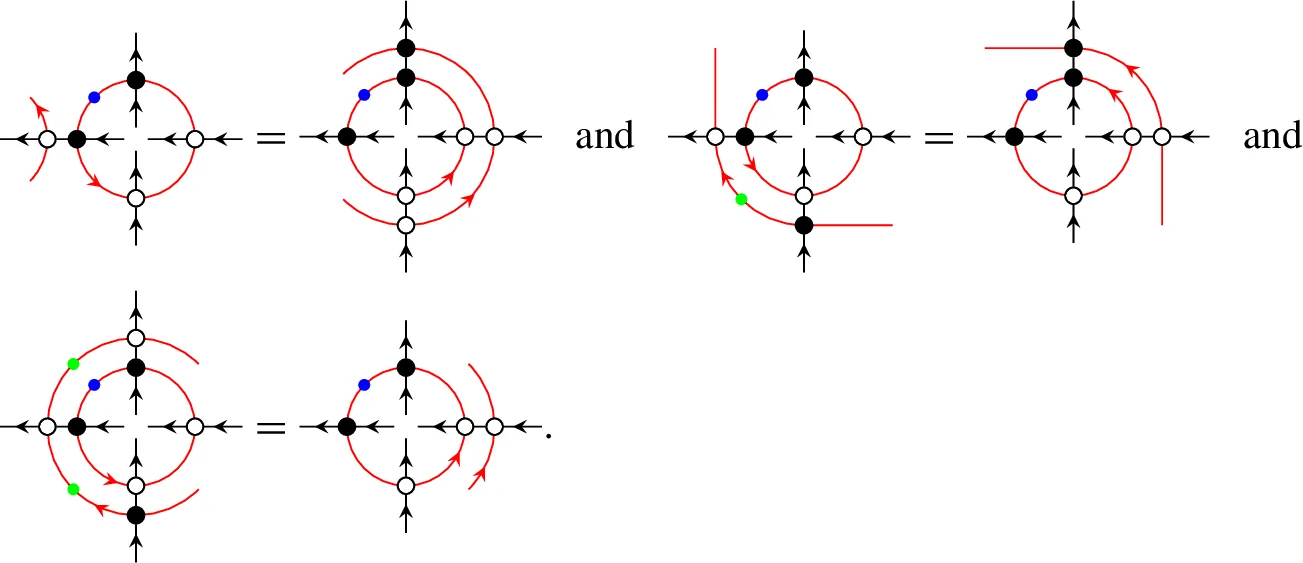

These identities are obtained from the previous ones by applying *T* on the two tensor components on the bottom and on the right.

As $g\in \mathcal {A}^*$ is group-like, applying *g* on the product of MPO tensors is the same as applying it on the individual tensors. For example, applying the linear functional *g* on the upper left tensor component of the equation

**Figure:**
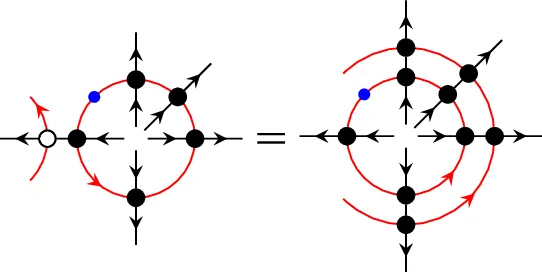

results in the equation

**Figure:**
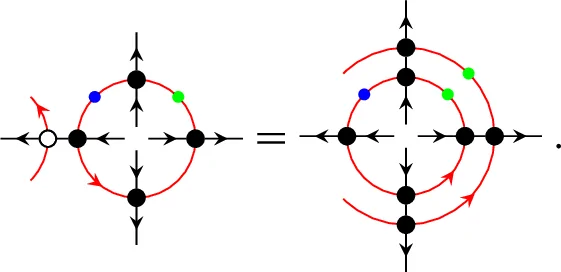

Non-degeneracy of Ω means that the action of the linear functional λ defined by $(\lambda \otimes \textrm{Id})\circ \Delta (\Omega )=1$ removes closed loops:

**Figure:**
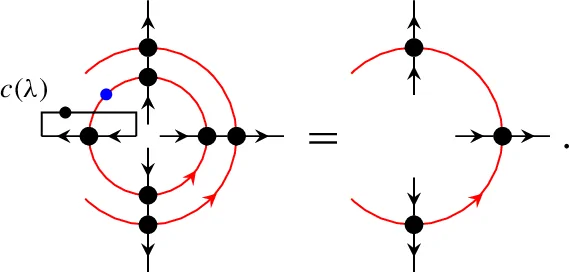

Finally note that the above equations also hold for non-translation invariant MPO representations of the pulling-through algebra.

## MPO-Injective PEPS

In this section we define projected entangled pair states that possess certain symmetries described by a pulling-through algebra $\mathcal {A}$. Such a PEPS will also be called $\mathcal {A}$-injective PEPS or MPO-injective PEPS. As the primary examples for pulling-through algebras are pivotal cosemisimple weak Hopf algebras, and the representations of such algebras form a pivotal fusion category, our definition can be translated into a category theoretical language. In fact, when the pulling-through algebra corresponds to a $C^*$-WHA, we believe that our definition is equivalent to the formalism presented in [LFH+20]. We expect that the pulling-through structure is actually more general than cosemisimple weak Hopf algebras; for example, certain non-semisimple pivotal Hopf algebras will also admit a pulling-through structure. States corresponding to those models have unusual properties. We show that MPO-injectivity is a topological property in the sense that it is invariant under the blocking of tensors (and thus under renormalization); in fact, our definition has been designed to satisfy this property. We also show, in the special case where $\mathcal {A}$ is a $C^*$-Hopf algebra, the relation between this PEPS and the generalization of Kitaev’s toric code to $C^*$-Hopf algebras [BMCA13]. Following [BMW+17], we then construct a set of states that we call local excitations and that are states that differ only locally from the PEPS. We also construct a set of local operators that form a representation of the Drinfeld double $\mathcal {D}(\mathcal {A}^{*})$ of the Hopf algebra $\mathcal {A}^{*}$ and show that these local operators transform the local excitations among each other. We identify the anyons of the model as the irrep sectors of the local excitations under the action of these local operators.

Let us first recall the definition of two dimensional PEPS. A 2D PEPS is defined on a directed pseudo-graph^10^$\mathcal {G}= (\mathcal {V},\mathcal {E})$ that can be drawn on an orientable 2-manifold manifold such that no edges intersect (like in the case of a planar graph). The figure below shows a local part of such a graph (locally it *is* a planar graph). For convenience we have numbered the vertices and we have displayed a circular arrow around vertex 5 depicting the orientation of the 2-manifold:

**Figure:**
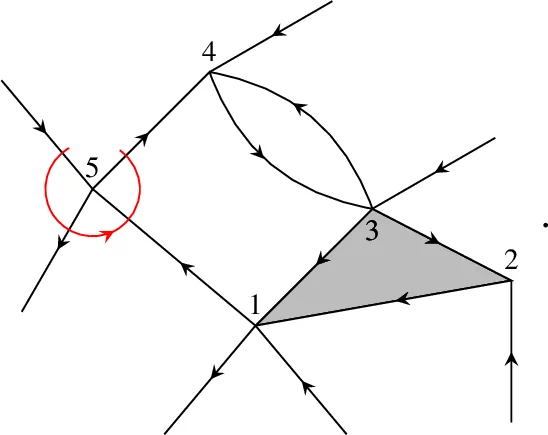

The areas enclosed by minimal (unoriented) cycles in the graph are called plaquettes. Such a plaquette is denoted by light gray shading between the vertices 1, 2 and 3. Let $\mathcal {E}^{op}$ denote the set of edges in $\mathcal {E}$ with reversed orientation, and for any $e\in \mathcal {E}$ or $e\in \mathcal {E}^{op}$ let $\bar{e}$ denote the edge *e* with the opposite orientation.

To every edge $e\in \mathcal {E}$ we assign a finite dimensional complex vector space, $V_e$. Let us also assign a vector space $V_{\bar{e}}$ to every oppositely oriented edge $\bar{e}\in \mathcal {E}^{op}$ such that $V_{\bar{e}} = V_e^*$. Note that this relation is symmetric, i.e., $V_{e} = V_{\bar{e}}^* = V_{\bar{\bar{e}}}$. To every vertex we assign a (finite dimensional) Hilbert space, $\mathcal {H}_v$. Finally, to each vertex *v* we assign a tensor $A_v\in \mathcal {H}_v \otimes \bigotimes _{e\in \mathcal {N}_v} V_{e}$, where $\mathcal {N}_v$ denotes the set of edges $e\in \mathcal {E}\cup \mathcal {E}^{op}$ that connect *v* with another vertex such that the orientation of *e* points away from *v*. Given all these data, the state defined by the PEPS is obtained by contracting $\bigotimes _v A_v$ along the edges of the graph; note that this contraction is possible, because if *e* is an edge between *v* and *w*, then the tensor component corresponding to *e* in the tensor $A_v$ is $V_e$, while in the tensor $A_w$ it is $V_{\bar{e}} = V_e^*$. The state defined by the PEPS can be represented, using the graphical notation of tensor calculus, as

**Figure:**
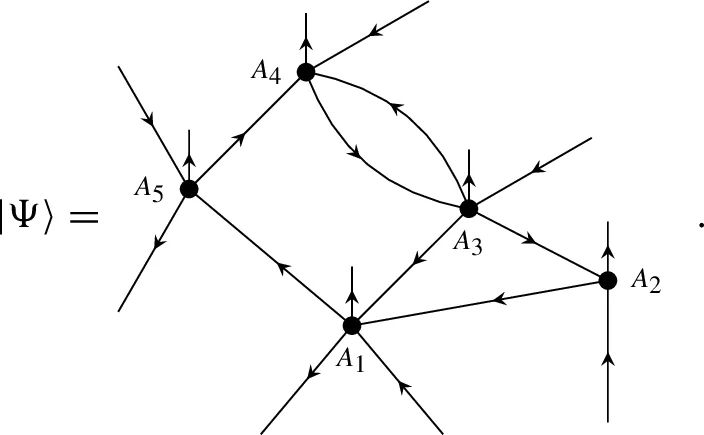

Let us now define MPO-injective PEPS. These are PEPS such that the PEPS tensors are invariant under certain symmetry operations acting on their virtual degrees of freedom, i.e. on the tensor components $\bigotimes _{e\in \mathcal {N}_v} V_{e}$ of the tensor $A_v$. More precisely, a PEPS tensor $A_v\in \mathcal {H}_v\otimes \bigotimes _{e\in \mathcal {N}_v} V_{e}$ is called $O_v$-injective for an operator $O_v\in \textrm{End}(\bigotimes _{e\in \mathcal {N}_v} V_{e})$, if there are tensors $B_v$ and $C_v$ such that

**Figure:**
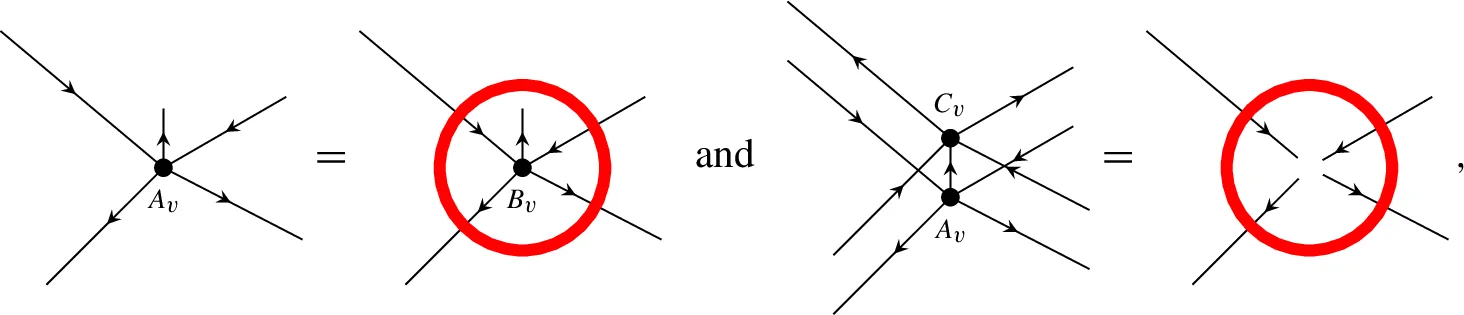

where the red circle denotes the operator $O_v$. Note that if $O_v$ is a projector, then one can choose $B_v = A_v$. If, on the other hand, $O_v$ is nilpotent, $B_v$ and $A_v$ are different. This is the case when the symmetries of the tensor form a non-semisimple algebra. In the following, we will define the symmetry operators $O_v$ for each vertex $v\in \mathcal {V}$. To do so, we need the following additional data. First, a pulling-through algebra $(\mathcal {A},\Omega ,T,g)$, and second, representations $\phi _{e}:\mathcal {A}\rightarrow \textrm{End}(V_{e})$ on the vector spaces $V_{e}$ such that $\phi _{\bar{e}} = \bar{\phi }_{e}$, where $\bar{\phi }(x) = [\phi \circ T(x)]^T$ for all $x\in \mathcal {A}$. Note that as *T* is idempotent, this relation is symmetric: if $\phi _{\bar{e}} = \bar{\phi }_{e}$, then $\phi _{e} = \bar{\phi }_{\bar{e}}$ as well. Finally, for every plaquette in the graph, we will choose a vertex from the ones surrounding the plaquette. Below we denote such a choice by putting a black dot close to the vertex chosen inside each plaquette:

**Figure:**
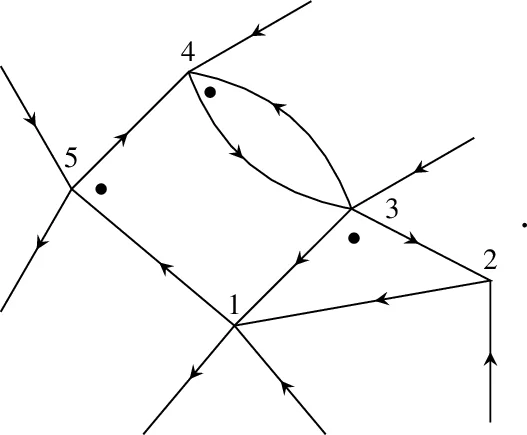

The symmetry operator $O_v$ around each vertex *v* is an MPO representation of the element Ω of the pulling-through algebra $\mathcal {A}$. The concrete form of the MPO is designed such that it is a generalization of *G*-injective PEPS [SCP10] and such that the PEPS remains MPO-injective even after blocking. Let us explain through an example what this MPO representation exactly is. The symmetry operator $O_v$, around the vertex 3, takes the following form:

**Figure:**
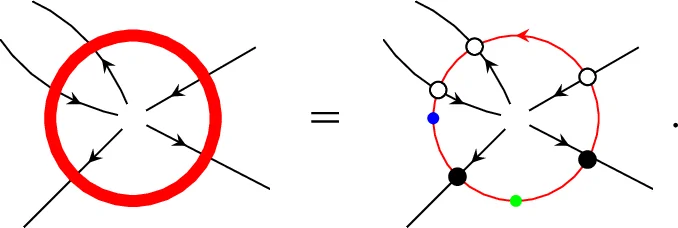

As stated above, this MPO is an MPO representation of Ω. The blue dot represents the boundary b(Ω); remember that as Ω is cocommutative, the placement of the boundary is not relevant. The orientation of the virtual index of the MPO follows the orientation of the surface, as the arrow on it shows. The MPO consists of two different MPO tensors. For an outgoing edge *e*, we use the MPO tensor denoted by black dots given by

**Figure:**
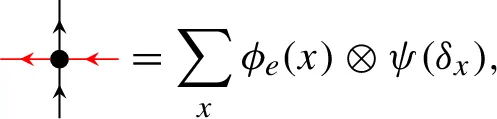

where *x* runs over a basis of $\mathcal {A}$ and ψ is a representation of $\mathcal {A}^*$ on the virtual vector space denoted by red indices. For an incoming edge *e*, we use the white MPO tensor given by

**Figure:**
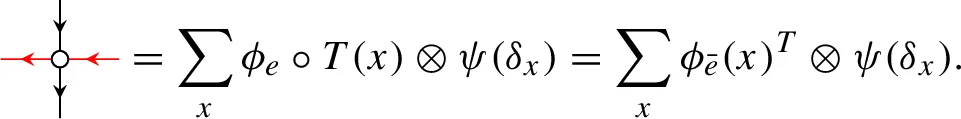

Note that this white tensor is constructed the same way as the black tensor, but for the construction we use the wrong representation: $\phi _{\bar{e}}$ instead of $\phi _e$ (in the formula transpose appears because on the l.h.s. we read the tensor as a linear map from the bottom to the top). Changing the orientation of the edge *e* (i.e. replacing it with $\bar{e}$) changes the black tensors to white and white tensors to black. Finally, as the vertex 3 was selected for the plaquette surrounded by vertices 1, 2 and 3, we insert the linear functional *g* between the edges connecting the vertex 3 to the vertices 1 and 2.

Using the above definition of an MPO-injective PEPS tensor, an MPO-injective PEPS—after applying the inverse tensors $C_v$ at each vertex *v*—can be written as the following tensor network:

**Figure:**
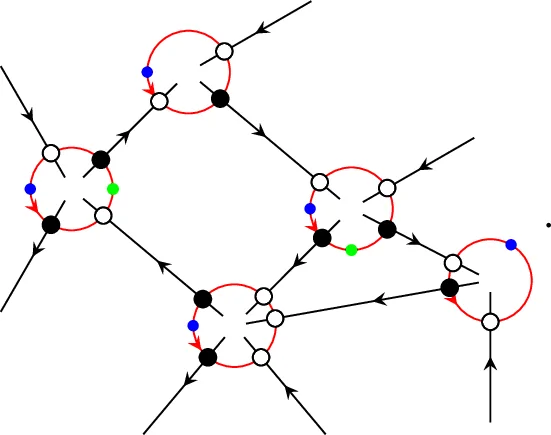

In general pulling-through algebras (such as a pulling-through algebra that originates from a pivotal WHA that is not a $C^*$-WHA) different placements of the group-like elements *g* lead to different states. In a pulling-through algebra that originates from a $C^*$-WHA, however, all these states are related to each other by local operations, and in fact, one can define MPO-injective PEPS in a more translation invariant way. To understand why, recall [BNS99] that in a $C^*$-WHA the positive spherical element $g\in \mathcal {A}^*$ can be written as $g=g_L g_R^{-1} = g_R^{-1} g_L$ such that $g_R=S(g_L)$ and such that there are algebra elements $G_L,G_R\in \mathcal {A}$ satisfying the following relations:

**Figure:**
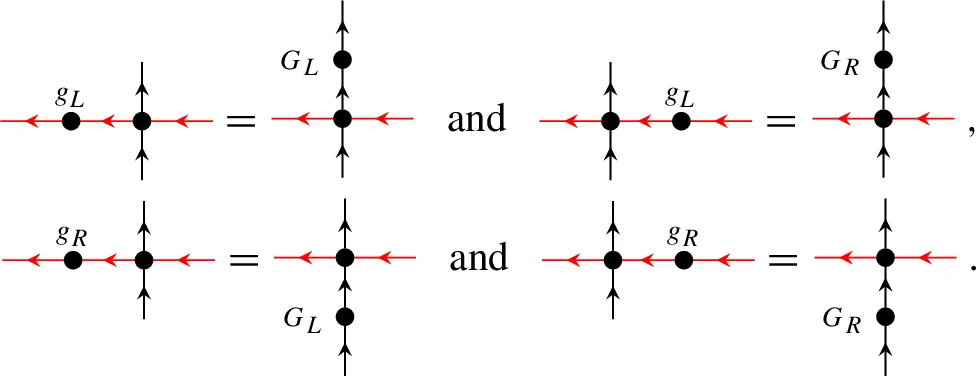

The equation $G_L = S(G_R)$ also holds and it is easy to check that $G_L = T(G_R)$ holds as well. Applying thus *T* on the previous relations, we obtain that the white tensors satisfy

**Figure:**
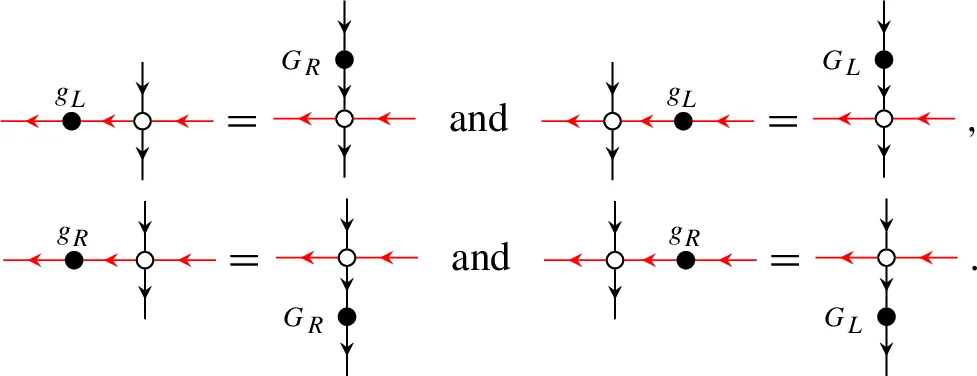

Let us write now $g=g_L g_R^{-1}$ in the definition of the MPO-injective PEPS. Using the relations above, we obtain then that $g_R^{-1}$ translates to a local operator acting on the vertex which *g* belongs to, while $g_L$ is delocalized around the plaquette. Let us illustrate this fact by depicting one of the plaquettes of the previous PEPS:

**Figure:**
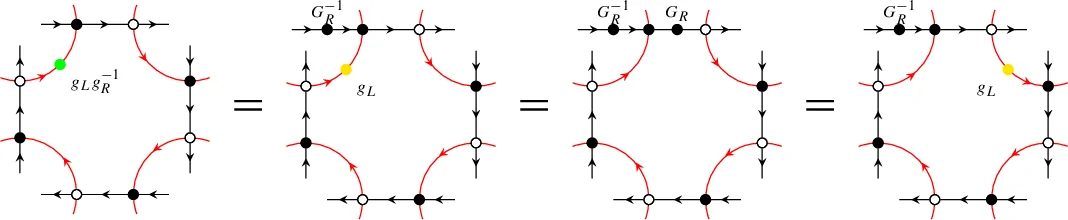

Applying thus $G_R$ on the appropriate tensor components, we obtain that the state defined by

**Figure:**
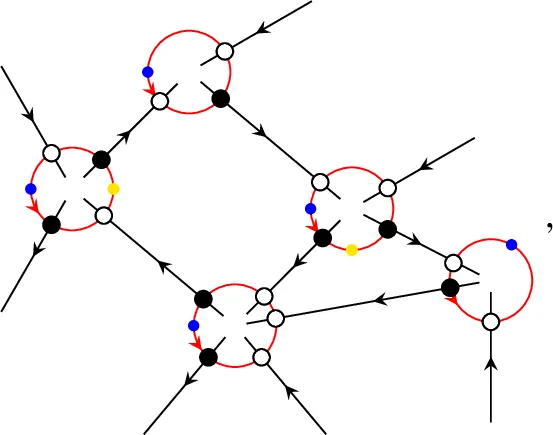

where the yellow dot now denotes $g_L$ instead of $g=g_Lg_R^{-1}$, is also MPO-injective and in this state the yellow dots move freely around the plaquette:

**Figure:**
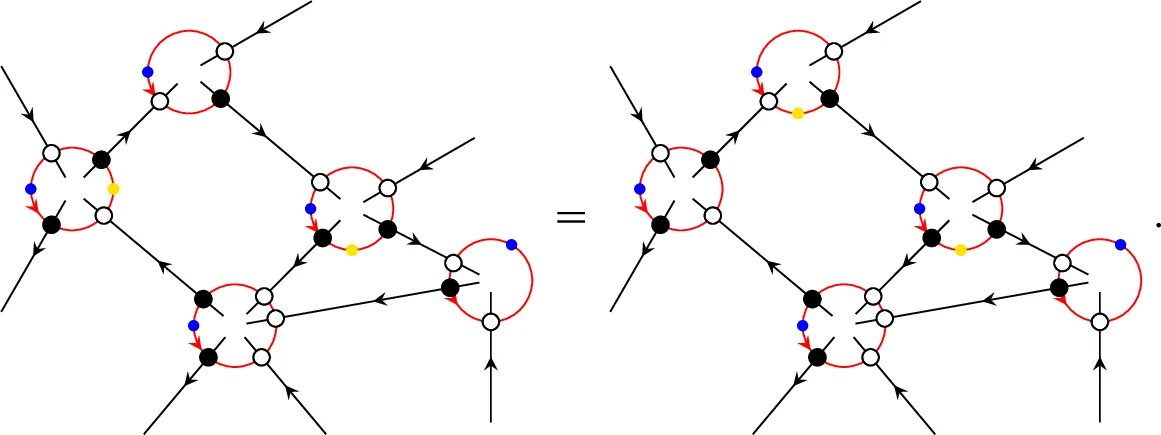

As we have seen above, the yellow dot does not even have to appear on the virtual index of the MPO, it can be instead inserted between two PEPS tensors anywhere around the plaquette.

### Scale independence

In this section we introduce an operation on states that we call *blocking* and show that an MPO-injective PEPS stays MPO-injective even after blocking. Blocking is the basis of renormalization (there it is followed by an isometry that gets rid of certain degrees of freedom) and has a natural representation in tensor networks. Blocking simply means that we treat certain – neighboring—particles together: for example, given a three-partite state $\vert \psi \rangle \in \mathcal {H}_1 \otimes \mathcal {H}_2 \otimes \mathcal {H}_3$, the blocking of particles 2 and 3 means that we reinterpret $\vert \psi \rangle$ as a two-partite state in $\mathcal {H}_1 \otimes \mathcal {H}_{23}$, where $\mathcal {H}_{23} = \mathcal {H}_2 \otimes \mathcal {H}_3$.

In PEPS, blocking is a partial contraction of the tensor network, i.e., that in a given region we replace all tensors by one tensor that is the result of the contraction of the tensors in that region. The blocked tensor network is then another, coarser tensor network. For example, in the tensor network below blocking of four tensors results in the following new tensor network:

**Figure:**
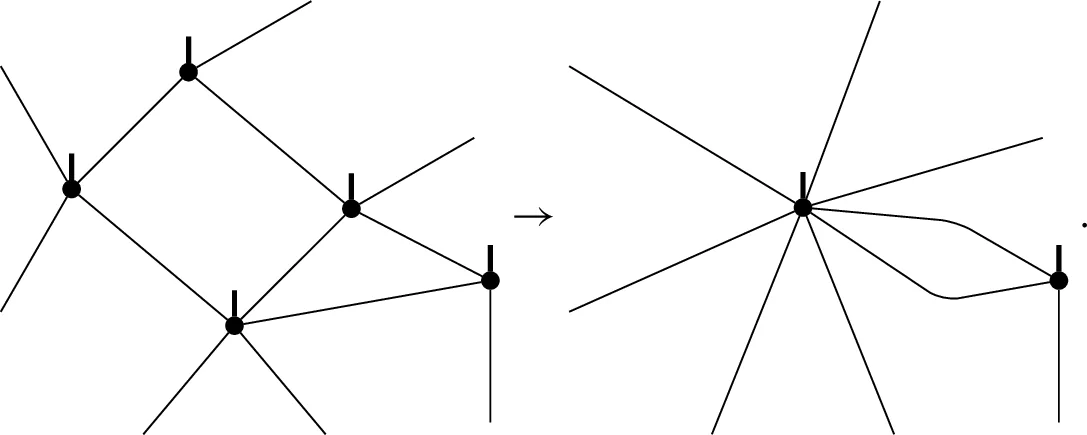

As the example above shows, sometimes two edges *e* and *f* that were distinct edges in the original PEPS become a single double edge in the blocked tensor network. We can block these two edges together, i.e. consider $V_e\otimes V_f$ as a single vector space. Pictorially we express this as writing a single edge instead of the two distinct ones:

**Figure:**
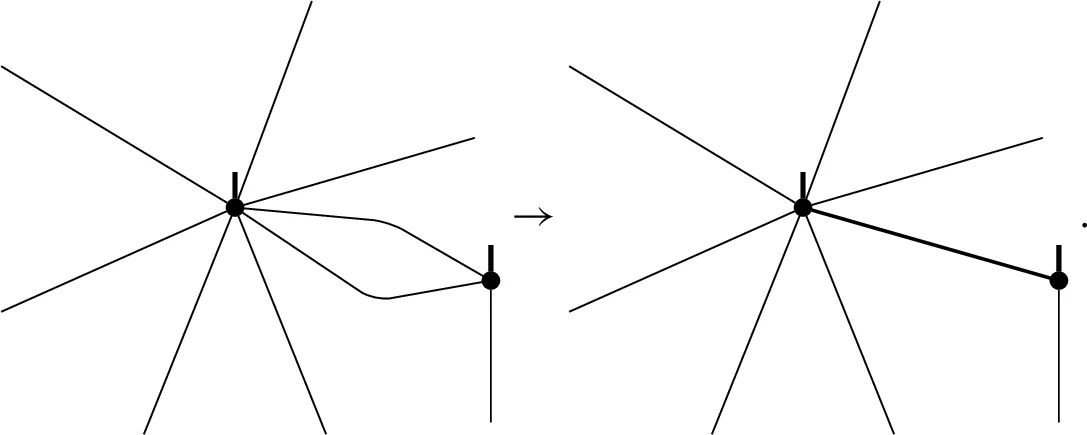

In fact, every blocking can be broken down into a series of simple steps: in each step we either block two neighboring tensors that are connected by a singe edge, possibly leading to double edges in the PEPS, or block two edges together to remove a double edge from the PEPS. Note that in the first case the number of plaquettes does not change (if there are double edges after blocking, the area enclosed between the two edges is considered as a plaquette), while in the second case the number of plaquettes decreases by one.

In the following, we will show that an MPO-injective PEPS stays MPO-injective even after blocking. Let us consider a pulling-through algebra $\mathcal {A}$ and an $\mathcal {A}$-injective PEPS. We first show that blocking two neighboring tensors in the $\mathcal {A}$-injective PEPS results in another $\mathcal {A}$-injective PEPS, and then, that removing double edges does not change the $\mathcal {A}$-injectivity property either.

Let us now consider the blocking of two neighboring $\mathcal {A}$-injective PEPS tensors that are connected to each other with a single edge. After applying the inverse tensors on the two PEPS tensors and using the pulling-through property, we obtain

**Figure:**
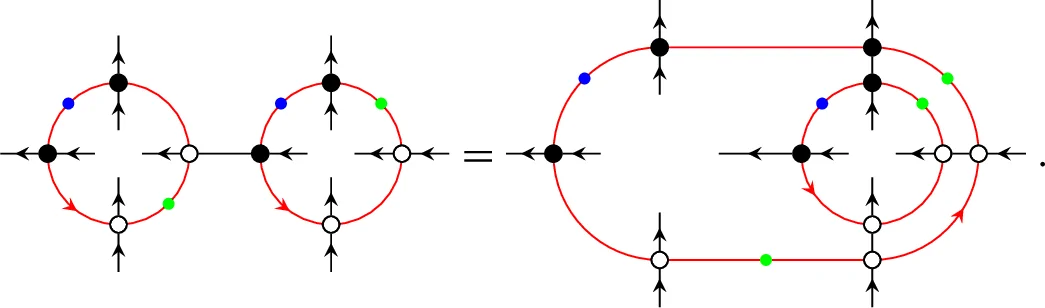

Due to non-degeneracy, applying a suitable linear functional to the inner two indices, the inner MPO disappears leading to a single MPO on the boundary:

**Figure:**
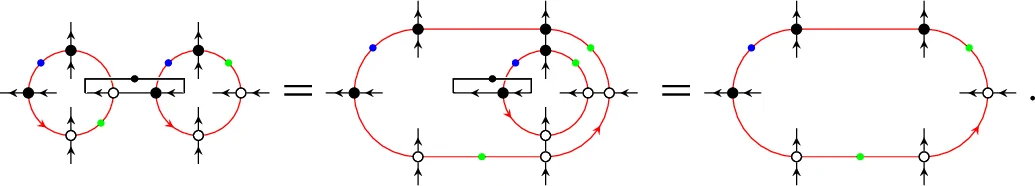

We have thus constructed an inverse tensor (the inverse tensors of the individual PEPS tensors contracted with the matrix describing the linear functional used above) for the blocked tensor, and showed that it is MPO-injective. Let us note that in the process neither the “outgoing arrow is black tensor, incoming arrow is white tensor” nor the “one green dot per plaquette” property have changed, and thus not only the blocked tensor is MPO-injective but the whole PEPS remains MPO-injective as well.

Let us now consider the blocking of two neighboring edges *e* and *f*. Assume that the edges are oriented in the opposite direction and that the “one green dot per plaquette” is on the left vertex:

**Figure:**
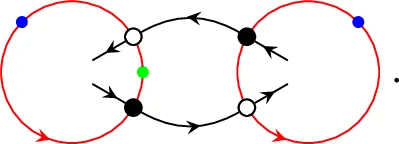

Here we did not indicate the other MPO tensors on the two vertices. In order to block these two edges together, let us first reorient the lower edge, *f*. The reorientation of the arrow implies that we change the corresponding vector space $V_f$ to $V_{\bar{f}}$ and the representation $\phi _f$ to $\phi _{\bar{f}}$. This also changes the black and white tensors and thus the tensor network notation becomes:

**Figure:**
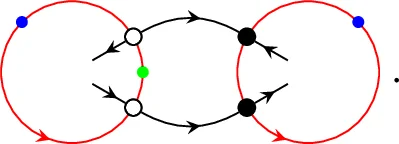

Blocking the edges *e* and *f* now simply means replacing the two individual $\mathcal {A}$-modules $V_e$ and $V_f$ by their tensor product $V_f\boxtimes V_e$. After blocking, we obtain a single edge with two MPO tensors at the two ends:

**Figure:**
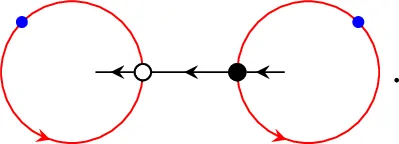

Here the black MPO tensor is built using the representation $\phi _{fe} = \phi _f \boxtimes \phi _e$, while the white MPO tensor is built using the transpose of the representation $\bar{\phi }_{fe}$,

$$\begin{aligned} \phi _{fe} \circ T = (\phi _{f} \otimes \phi _e) \circ \Delta \circ T = (\phi _f \circ T \otimes g \otimes \phi _e \circ T) \circ \Delta _{\textrm{op}}^2. \end{aligned}$$

This equation shows that the single (blocked) white tensor is the concatenation of the two white tensors with the green dot in the middle. The fact that the equation contains $\Delta _{\textrm{op}}$ instead of Δ simply reflects the fact that as the orientation of the two circles are the same, the arrow on the virtual index (red line) on the left is oriented in the opposite direction as on the right.

As the blocking leaves the rest of the PEPS invariant, blocking two edges keeps the PEPS MPO-injective. We have thus shown that MPO-injectivity is invariant both under the blocking of neighboring vertices and under the blocking of neighboring edges. As blocking any number of tensors in a simply connected region can be decomposed into a series of such simple blocking steps, we have proven that MPO-injectivity is invariant under blocking.

### Relation to the Kitaev model

The tensor networks defined above are strongly linked to the generalized Kitaev models defined in [BMCA13]. In this section we show the concrete connection. First, let us consider an MPO-injective PEPS constructed from a $C^*$-Hopf algebra $\mathcal {A}$. Based on the construction of this PEPS, we can define another multi-partite state $\vert \Psi \rangle$ where the individual degrees of freedom are described on the Hilbert space $\mathcal {A}$. Second, we construct a parent Hamiltonian for $\vert \Psi \rangle$—the Kitaev Hamiltonian—that is the sum of commuting projectors. Finally, we define the Drinfeld double $D(\mathcal {B})$ of any Hopf algebra $\mathcal {B}$ and construct a set of local operators that form a representation of $D(\mathcal {A}^*)$; we also construct a set of local deformations of $\vert \Psi \rangle$ and show that this set of states *S* is invariant under the action of $D(\mathcal {A}^*)$. We identify the anyons of the model as the irreps sectors of *S* under the action of $\mathcal {D}(\mathcal {A}^*)$.

Let us first show how the state $\vert \Psi \rangle$ is defined. For that, consider the PEPS defined by a $C^*$-Hopf algebra. The construction of these states is easier to understand than in the general case, because there are no group-like elements *g* present in it (as g=ϵ). The PEPS, after applying the inverse tensors, read as

**Figure:**
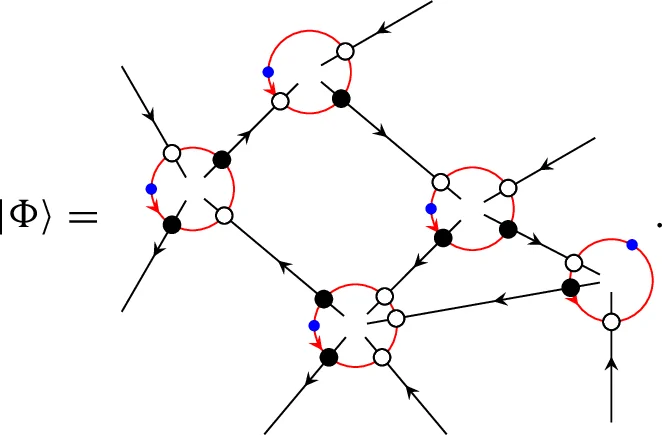

This vector $\vert \Phi \rangle$ lives in the vector space $\bigotimes _{v\in \mathcal {V}} \left( \bigotimes _{e\in \mathcal {N}_v} V_{e} \right)$, because at every vertex *v* the degrees of freedom are $\bigotimes _{e\in \mathcal {N}_v} V_{e}$. Note that in this tensor product every edge appears exactly twice, once with the orientation defined by the graph and once with opposite orientation. Let us now rearrange this tensor product and group together the degrees of freedom corresponding to the two orientations of each edge. After this regrouping, we can interpret the previous vector space as $\bigotimes _{e \in \mathcal {E}} \left( V_{e} \otimes V_{\bar{e}}\right)$. Finally note that as $V_{\bar{e}} = V_e^*$ and for every finite dimensional vector space *V*, the tensor product $V\otimes V^*$ is isomorphic to $\textrm{End}(V)$, this vector space can also be interpreted as $\bigotimes _{e \in \mathcal {E}} \textrm{End}(V_e)$, i.e. one can think of $\vert \Phi \rangle$ as

$$\begin{aligned} \vert \Phi \rangle \in \bigotimes _{e \in \mathcal {E}} \textrm{End}(V_e). \end{aligned}$$

Note that, by construction, $\vert \Phi \rangle$ is not supported on the whole Hilbert space $\bigotimes _{e \in \mathcal {E}} \textrm{End}(V_e)$, but instead only on the subspace $\bigotimes _{e \in \mathcal {E}} \phi _{e} (\mathcal {A})$, where $\phi _{e}$ is the representation of $\mathcal {A}$ used on the edge *e*. This implies that we can define another vector, $\vert \Psi \rangle$, as

$$\begin{aligned} \vert \Psi \rangle = \left( \bigotimes _{e \in \mathcal {E}} \phi _{e}^{-1}\right) \vert \Phi \rangle \in \bigotimes _{e \in \mathcal {E}} \mathcal {A} . \end{aligned}$$

If $\mathcal {A}$ is a $C^*$-Hopf algebra, there is a scalar product on $\mathcal {A}$, and thus the vector space $\bigotimes _{e \in \mathcal {E}} \mathcal {A}$ is a finite dimensional Hilbert space. Therefore $\vert \Psi \rangle$ can be interpreted as a (possibly unnormalized) state. The maps $\phi _{e}^{-1}$ act locally, therefore this state is again a PEPS—the virtual legs of the PEPS tensor are the red lines in the figure above. This PEPS is then the same (up to a choice of orientation of the arrows on both the red and black lines) as the one described in [BMCA13].

#### The Kitaev Hamiltonian

Building on the results of [BMCA13], in this section we explicitly construct the Kitaev parent Hamiltonian for the state $\vert \Psi \rangle$ defined in the previous section. To make the reading easier, we will use a graphical language to depict the action of the defined Hamiltonian. This graphical language is nothing but the tensor network representation of the state using the representation $\bigotimes _{e\in \mathcal {E}} \phi _{e}$. For simplicity, let us restrict ourselves to a square lattice, and fix the orientation of the lattice such that all vertical edges point from bottom to top and all horizontal ones from right to left (i.e. the product of two elements on the vertical edge reads from top to bottom and on the horizontal edges from left to right).

The Kitaev Hamiltonian consists of two type of terms, the plaquette terms $B_p$ and vertex terms $A_v$. The total Hamiltonian is the sum of these terms,

$$\begin{aligned} H = - \sum _{p\in \text {plaquettes}} B_p - \sum _{v\in \text {vertices}} A_v. \end{aligned}$$

Each plaquette term $B_p$ acts on the edges surrounding the plaquette *p*, while each vertex term $A_v$ act on the edges connected to the vertex *v*. We will define $B_p$ and $A_v$ such that they are orthogonal projectors and any two such terms commute. The MPO-injective PEPS $\vert \Psi \rangle$ is a frustration-free ground state of this Hamiltonian.

Let us now define the operator $A_v$ for a given vertex *v*. As stated above, this operator acts on the edges surrounding the vertex *v*. On these four particles, its action is given by:

$$\begin{aligned} A_v : x\otimes y\otimes z\otimes v \mapsto \sum _{(\Omega )} x \cdot \Omega _{(1)} \otimes S(\Omega _{(2)}) \cdot y \otimes S(\Omega _{(3)}) \cdot z \otimes v\cdot \Omega _{(4)} \ , \end{aligned}$$

where Ω is the Haar integral of $\mathcal {A}$, *x* is the particle above the vertex, *y* is the one on its right, *z* is the one below and *v* is the one on the left. The concrete form of $A_v$ depends on the orientation of the lattice. Using the graphical representation, it is easier to visualize the action of $A_v$:

**Figure:**
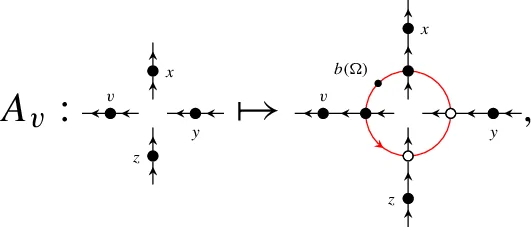

i.e. it multiplies the four particles by the (translation invariant) MPO $\Delta ^3(\Omega )$, each from the side that is closer to the vertex. As Ω is a projector, it is clear that $A_v$ is a projector as well. As both representations $x \mapsto (y\mapsto yx)$ and $x \mapsto (y\mapsto S(x)y)$ of $\mathcal {A}^{op}$ are ∗-representations, $A_v$ is also self-adjoint. Note that for any two different vertices $v_1$ and $v_2$ the Hamiltonian terms $A_{v_1}$ and $A_{v_2}$ clearly commute: if $v_1$ and $v_2$ are not neighboring vertices, they act on different particles; if $v_1$ and $v_2$ are neighboring, there is a single particle on which both of them acts, but if $A_{v_1}$ acts from the left, then $A_{v_2}$ acts from the right of the particle.

Let us now define the operator $B_p$ for a given plaquette *p*. As stated above, this operator acts on the edges surrounding the plaquette. On these four particles, its action is given by

$$\begin{aligned} B_p: x\otimes y \otimes z \otimes v \mapsto \sum _{xyzv} \omega (S(x_{(1)}) S(y_{(1)} z_{(2)} v_{(2)} ) ) \cdot x_{(2)} \otimes y_{(2)} \otimes z_{(1)} \otimes v_{(1)}, \end{aligned}$$

where ω is the Haar integral of $\mathcal {A}^*$ and *x* is the particle on the right of the plaquette, *y* is the one on top, *z* is the one on the left and *v* is the particle below the plaquette. Again, the action of $B_p$ is easier to understand using the graphical representation:

**Figure:**
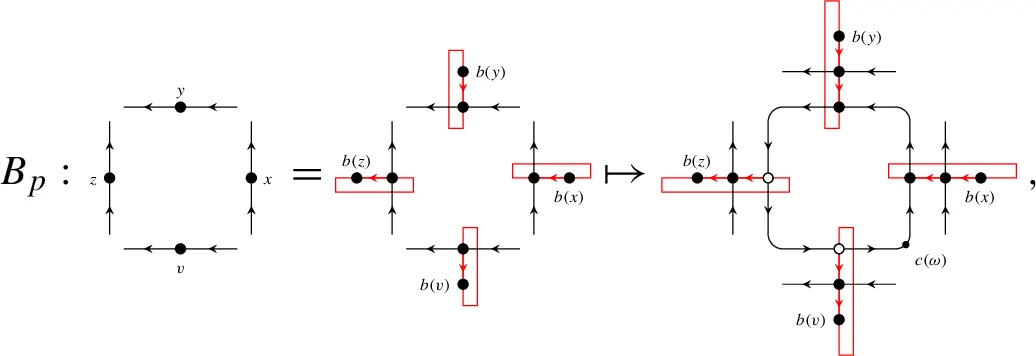

where the matrix c(ω) is the boundary describing the MPO representation of ω, see Eq. (4.14). Similar to the vertex terms, the operators $B_p$ are projectors, and as the representations $f \mapsto (x\mapsto x_{(1)} f(x_{(2)}))$ and $f\mapsto (x\mapsto f\circ S(x_{(1)}) \cdot x_{(2)})$ are both ∗-representations of $\mathcal {A}^*$, they are also self-adjoint. If $p_1$ and $p_2$ are plaquettes that are not neighboring, then $B_{p_1}$ and $B_{p_2}$ act on different particles, and thus they commute. If $p_1$ and $p_2$ are neighboring, then there is one particle both acts on. One of the operators, however, act from the right, the other from the left, and thus even in this case, $B_{p_1}$ and $B_{p_2}$ commute.

Let us now show that the operators $A_v$ and $B_p$ commute, i.e. that the Kitaev Hamiltonian is indeed a sum of commuting orthogonal projectors. If the vertex *v* is not a vertex on the plaquette *p*, then $A_v$ and $B_p$ are acting on different particles and thus they trivially commute. If the vertex *v* is one of the vertices around the plaquette, we first calculate the graphical representation of the action of $A_v B_p$ on the six particles surrounding both the plaquette and the vertex:

**Figure:**
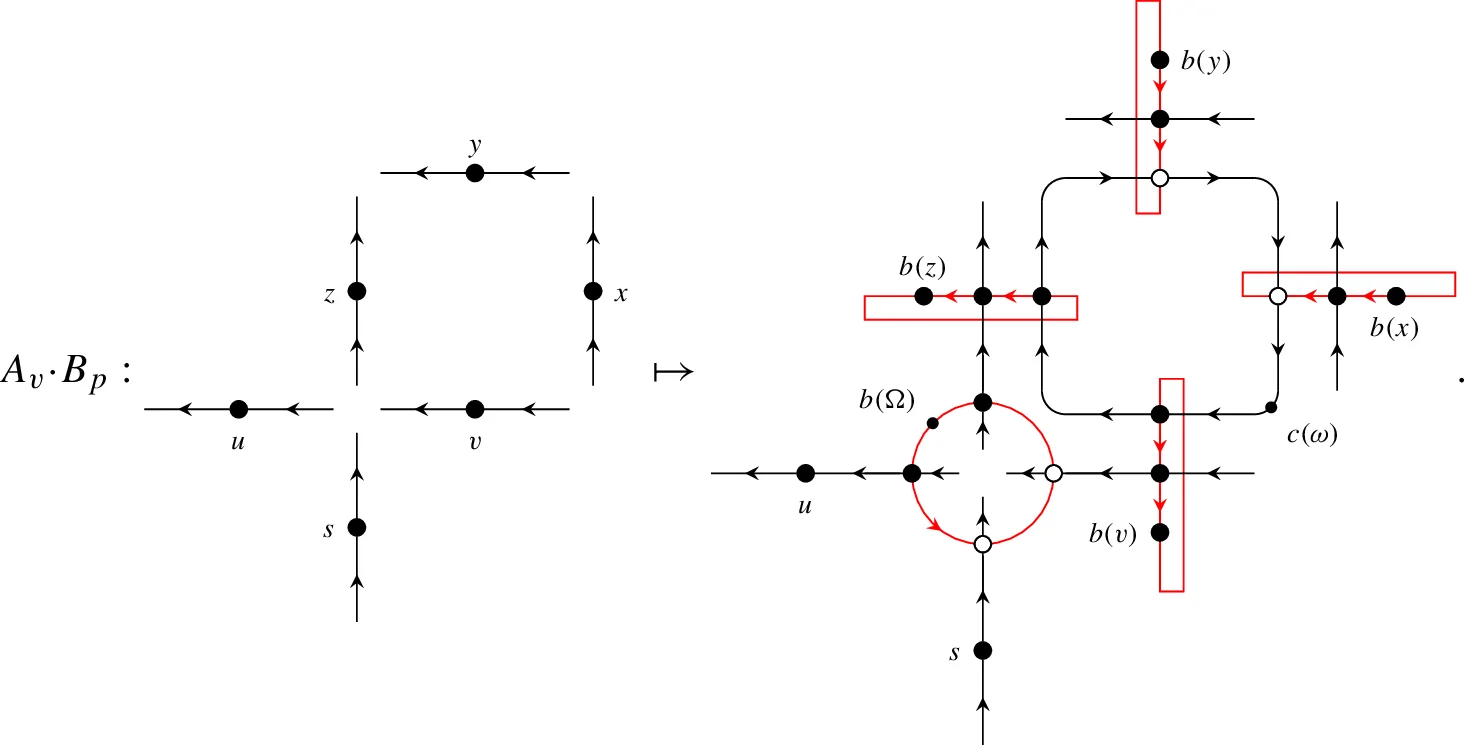

Let us now calculate the action of $B_p A_v$.

**Figure:**
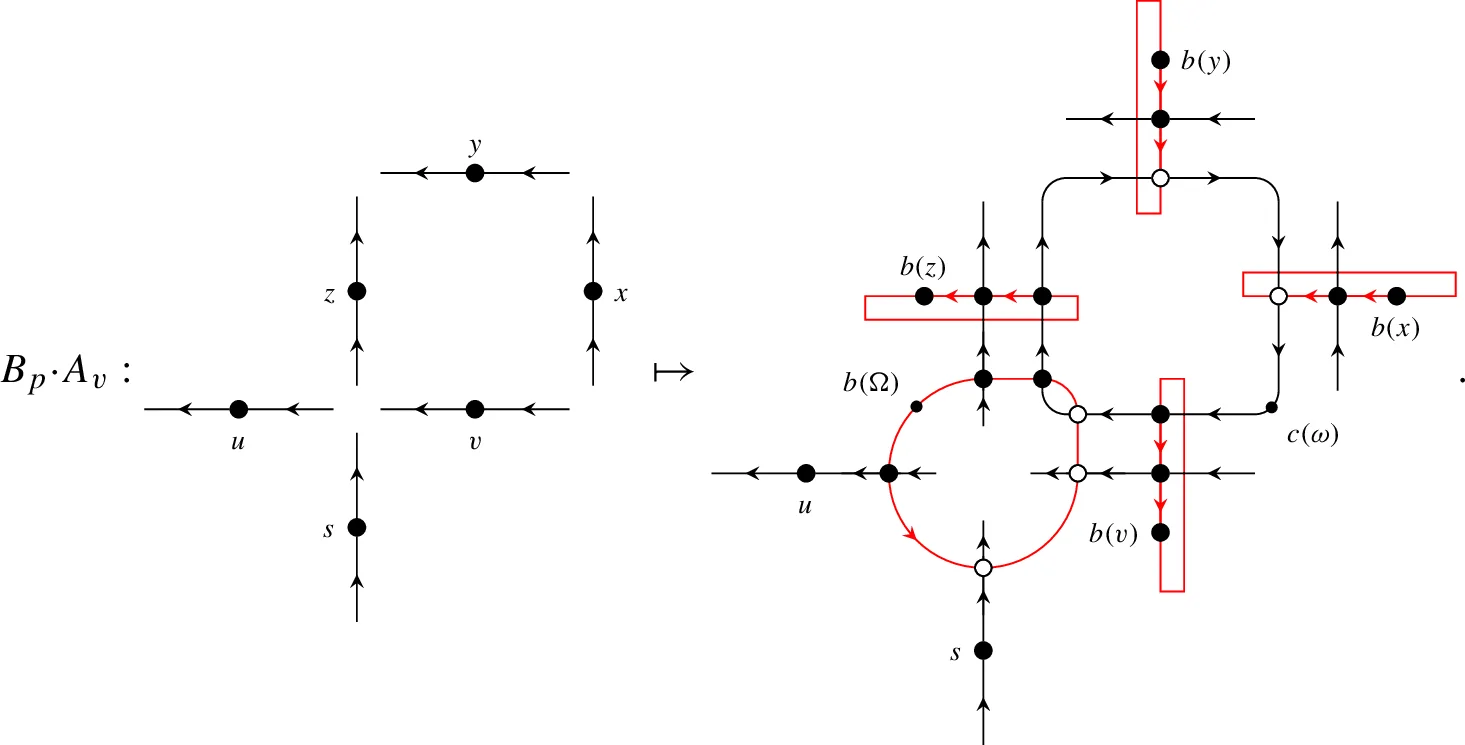

The two tensors in the middle—closed by arbitrary boundary condition *b*(*w*) on the virtual index and after straightening the physical index—read as:

**Figure:**
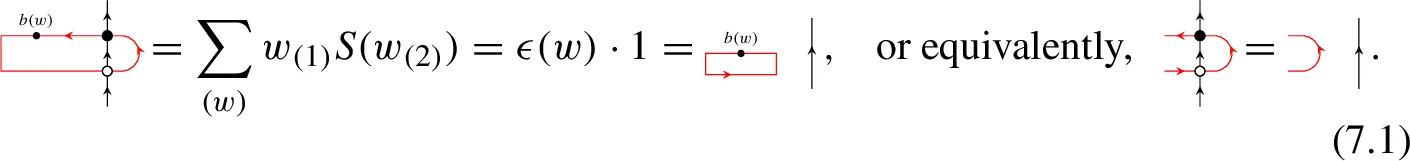

Using this identity in the expression for $B_p A_v$, we obtain that the two loops, the virtual and physical one, can be untangled, and thus $B_p A_v = A_v B_p$, i.e. the Hamiltonian terms $A_v$ and $B_p$ commute.

Let us finally show that the state $\vert \Psi \rangle$ is a ground state of the Hamiltonian *H*. First, as Ω is a projector, $\vert \Psi \rangle$ is clearly invariant under each term $A_v$. Let us now show that it is also invariant under all $B_p$, then this will mean that $\vert \Psi \rangle$ is a frustration-free ground state of *H*. To see that $\vert \Psi \rangle$ is invariant under the action of $B_p$, note that the state, locally around a plaquette, looks like

**Figure:**
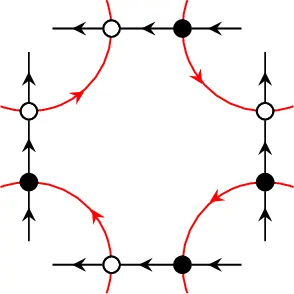

The action of $B_p$ is thus

**Figure:**
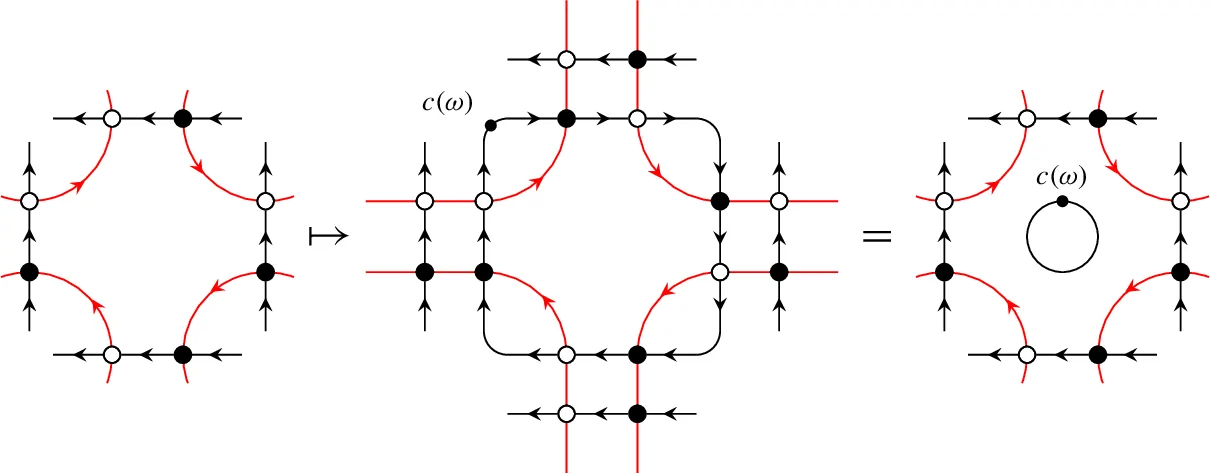

Where in the last equation we have used Eq. (7.1) in each corner. The result is therefore just a multiplication with the complex number ω(1)=1, i.e. the state $\vert \Psi \rangle$ is invariant under $B_p$.

#### The Drinfeld double and anyons

In this section we define the Drinfeld double $D(\mathcal {A})$ of a finite dimensional Hopf algebra $\mathcal {A}$ and, for each pair of plaquette *p* and neighboring vertex *v*, a set of local operators including the Hamiltonian terms $A_v$ and $B_p$ that forms a representation of $D(\mathcal {A}^*)$. We also define a set of sates that differ only locally from the state $\vert \Psi \rangle$. The defined local operators transform these states amongst each other, and thus these states form a $D(\mathcal {A}^*)$-module. We identify the anyons of the model as the subsets of states that form *irreducible* $\mathcal {D}(\mathcal {A}^*)$-modules (see also [BMW+17]).

Let us first define the Drinfeld double of a Hopf algebra.

##### Definition 7.1

Let $\mathcal {A}$ be a finite dimensional Hopf algebra. The Drinfeld double $\mathcal {D}(\mathcal {A})$ is a Hopf algebra constructed as follows. As a vector space, it is $\mathcal {A}^* \otimes \mathcal {A}$. Given $f\in \mathcal {A}^*$ and $x\in \mathcal {A}$ we will write f⋈x for their tensor product. The coproduct in $\mathcal {D}(\mathcal {A})$ is given by

$$\begin{aligned} \Delta (f\bowtie x) = \sum _{(f),(x)} (f_{(2)} \bowtie x_{(1)}) \otimes (f_{(1)}\bowtie x_{(2)}). \end{aligned}$$

The product in $\mathcal {D}(\mathcal {A})$ is given by

7.2 $$\begin{aligned} (f\bowtie x)\cdot (g\bowtie y) = \sum _{(g) (x)} g_{(1)}\circ S^{-1} (x_{(3)}) \cdot g_{(3)}(x_{(1)}) \cdot fg_{(2)} \bowtie x_{(2)}y. \end{aligned}$$

One can verify that the above product and coproduct indeed define a Hopf algebra. The unit of $\mathcal {D}(\mathcal {A})$ is $\epsilon \bowtie 1$. Linear functionals on $\mathcal {D}(\mathcal {A})$ are of the form x⋈f ($x\in \mathcal {A}$ and $f\in \mathcal {A}^*$) and in particular, the counit is given by $1\bowtie \epsilon$. Finally, the antipode in $\mathcal {D}(\mathcal {A})$ is given by $S(f\bowtie x) = S^{-1}(f)\bowtie S(x)$. If $\mathcal {A}$ is a $C^*$-Hopf algebra, then $\mathcal {D}(\mathcal {A})$ is also a $C^*$-Hopf algebra with ∗ operation $f\bowtie x \mapsto f^* \bowtie x^*$. Note that, as $\sum _{(\epsilon )} \epsilon _{(1)}\otimes \epsilon _{(2)}\otimes \epsilon _{(3)} = \epsilon \otimes \epsilon \otimes \epsilon$, the map

$$\begin{aligned} \mathcal {A} \rightarrow \mathcal {D}(\mathcal {A}), \ x \mapsto \epsilon \bowtie x \end{aligned}$$

is both a homomorphism and a cohomomorphism. Similarly, as $\sum _{(1)} 1_{(1)}\otimes 1_{(2)}\otimes 1_{(3)} = 1\otimes 1\otimes 1$, the map

$$\begin{aligned} \mathcal {A}^* \rightarrow \mathcal {D}(\mathcal {A}), \ f \mapsto f \bowtie 1 \end{aligned}$$

is a homomorphism and an anti-cohomomorphism. Using the images of these maps, all elements in the Drinfeld double can be written as

$$\begin{aligned} f\bowtie x = (f\bowtie 1) \cdot (\epsilon \bowtie x). \end{aligned}$$

The elements (f⋈1) and $(\epsilon \bowtie x)$ satisfy the following commutation relation:

$$\begin{aligned} (\epsilon \bowtie x) \cdot (f\bowtie 1) = \sum _{(f)(x)} f_{(1)}\circ S^{-1} (x_{(3)}) \cdot f_{(3)}(x_{(1)}) \cdot (f_{(2)} \bowtie 1) \cdot (\epsilon \bowtie x_{(2)}). \end{aligned}$$

Let $\mathcal {A}$ now be a finite dimensional Hopf algebra and $\mathcal {A}^*$ be its dual Hopf algebra. Let us now construct the Drinfeld double of $\mathcal {A}^*$. As a vector space, it is $\mathcal {A}\otimes \mathcal {A}^*$, and thus (as a vector space) it is canonically isomorphic to $\mathcal {D}(\mathcal {A})$. Let us make use of this isomorphism and write the elements of $\mathcal {D}(\mathcal {A}^*)$ as f⋈x instead of x⋈f. In this notation, the coproduct of the Drinfeld double $\mathcal {D}(\mathcal {A}^*)$ is given by

$$\begin{aligned} \Delta (f\bowtie x) = \sum _{(f),(x)} (f_{(1)} \bowtie x_{(2)}) \otimes (f_{(2)}\bowtie x_{(1)}), \end{aligned}$$

and the product is given by

7.3 $$\begin{aligned} (f\bowtie x)\cdot (g\bowtie y) = \sum _{(f) (y)} f_{(3)}\circ S^{-1} (y_{(1)}) \cdot f_{(1)}(y_{(3)}) \cdot f_{(2)}g \bowtie xy_{(2)}. \end{aligned}$$

Again, the maps

$$\begin{aligned} \mathcal {A} \rightarrow \mathcal {D}(\mathcal {A}^*): \ x \mapsto \epsilon \bowtie x \quad \text {and} \quad \mathcal {A}^* \rightarrow \mathcal {D}(\mathcal {A}^*):\ f\mapsto f\bowtie 1 \end{aligned}$$

are homomorphisms. Similar as above, every element of $\mathcal {D}(\mathcal {A}^*)$ can be written as

$$\begin{aligned} f\bowtie x = (\epsilon \bowtie x) \cdot (f\bowtie 1). \end{aligned}$$

The elements $(\epsilon \bowtie x)$ and (f⋈1) of $\mathcal {D}(\mathcal {A}^*)$ satisfy the commutation relation

7.4 $$\begin{aligned} (f\bowtie 1)\cdot (\epsilon \bowtie x) = \sum _{(f) (x)} f_{(3)}\circ S^{-1} (x_{(1)}) \cdot f_{(1)}(x_{(3)}) \cdot (\epsilon \bowtie x_{(2)}) \cdot (f_{(2)}\bowtie 1). \end{aligned}$$

Let us now define a set of local operators acting on an $\mathcal {A}$-injective PEPS and show that they form a representation of the Drinfeld double $\mathcal {D}(\mathcal {A}^*)$. All of these operators will act on the particles surrounding a neighboring plaquette and vertex pair (*p*, *v*). We will define two types of operators. The first type is denoted by $A_{(p,v)}^w$ for any $w\in \mathcal {A}$, it acts only on the particles surrounding the vertex and represents the element $\epsilon \bowtie w\in \mathcal {D}(\mathcal {A}^*)$. The second type is denoted by $B_{(p,v)}^f$ for any $f\in \mathcal {A}^*$, it acts only only on the particles surrounding the plaquette and it represents the element $f\bowtie 1\in \mathcal {D}(\mathcal {A}^*)$.

Let us first define the operators $A_{(p,v)}^w$ for a given plaquette *p*, vertex *v* and algebra element $w\in \mathcal {A}$. We define such an operator by its action on the four particles around the vertex; this action is defined by the graphical representation, using the injective representations $\phi _{(v,w)}$ on each edge. In the figure below, the plaquette *p* is in the upper right corner of the vertex. The action of $A_{(p,v)}^w$ is given by

**Figure:**
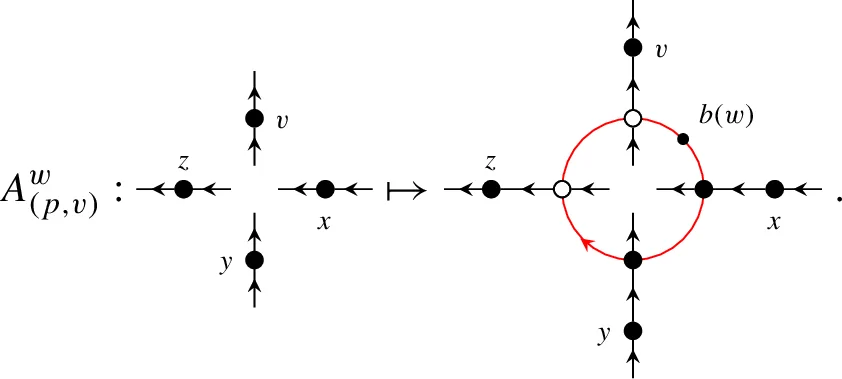

Note that the MPO representing this operator is oriented in the opposite way as in the definition of the PEPS and in the definition of the vertex term of the Hamiltonian. This expression reads as

$$\begin{aligned} A^w_{(p,v)} : x\otimes y\otimes z\otimes v \mapsto \sum _{(w)} w_{(4)} \cdot x \otimes w_{(3)} \cdot y \otimes z \cdot S(w_{(2)})\otimes v\cdot S( w_{(1)}) \ . \end{aligned}$$

Here and in what follows we omit the representations ϕ when translating between the figures and the algebraic formulas. It is easy to check that the operators $A_{(p,v)}^w$ form a representation of $\mathcal {A}$ and that this representation is a ∗-representation. Notice that the vertex term $A_v$ is exactly $A_{(p,v)}^\Omega$, because the Haar integral Ω is invariant under the antipode *S*:

**Figure:**
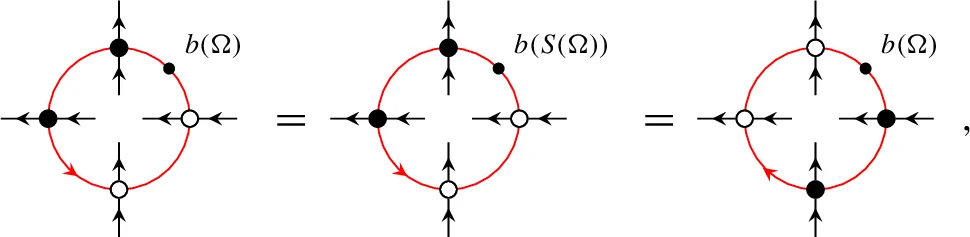

where the last equation is Eq. (5.21). The plaquette term $A_v$ is the multiplication with the MPO on the left, while $A_{(p,v)}^\Omega$ is multiplication with the MPO on the right. As Ω is cocommutative, the particular choice of the plaquette *p* does not matter, i.e. $A_v = A_{(p,v)}^\Omega$ holds for any choice of the plaquette *p* next to the vertex *v*.

Let us now define the operator $B_{(p,v)}^f$ for the plaquette *p*, vertex *v* and linear functional $f\in \mathcal {A}^*$. In the figure below, the vertex *v* is in the lower left corner of the plaquette *p*. The action of $B_{(p,v)}^f$ is given by

**Figure:**
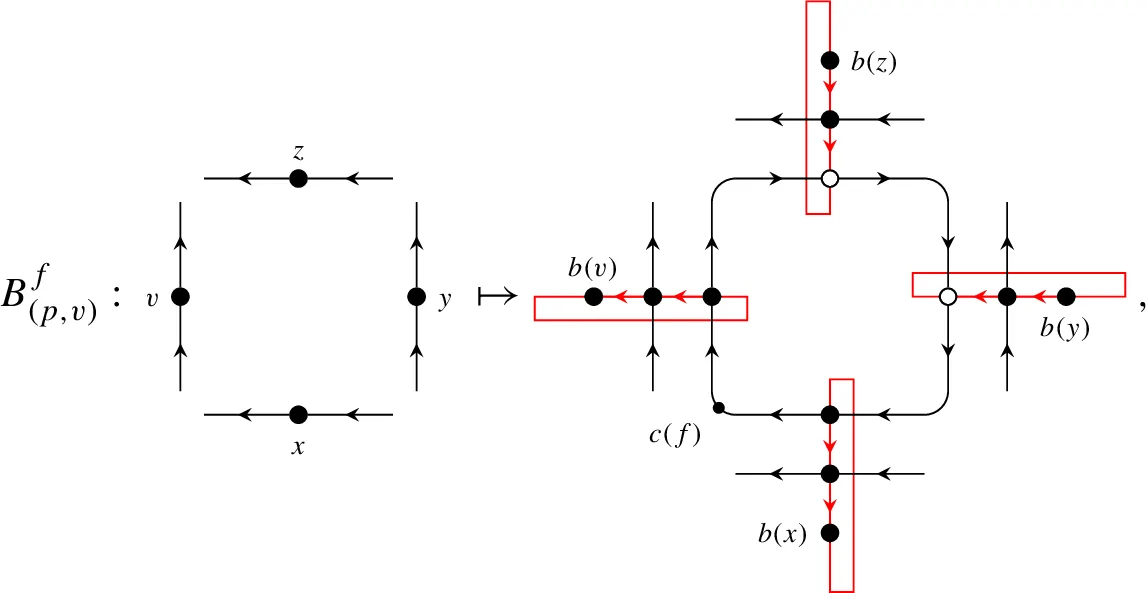

or equivalently, by

$$\begin{aligned} B_{(p,v)}^f: x\otimes y \otimes z \otimes v \mapsto \sum _{xyzv} f(x_{(2)} S(y_{(1)}) S(z_{(1)}) v_{(2)} ) \cdot x_{(1)} \otimes y_{(2)} \otimes z_{(2)} \otimes v_{(1)}. \end{aligned}$$

It is easy to check that these operators form a ∗-representation of $\mathcal {A}^*$. The plaquette term $B_p$ of the Kitaev Hamiltonian is exactly the operator $B_{(p,v)}^\omega$, where ω is the Haar integral of $\mathcal {A}^*$. As ω is cocommutative, the particular choice of *v* does not matter, i.e. $B_p = B_{(p,v)}^\omega$ for any vertex *v* around the plaquette *p*.

Let us now define a linear map $\mathcal {D}(\mathcal {A}^*) \rightarrow \textrm{End}(\mathcal {A}^6)$ for a given pair of plaquette *p* and vertex *v* by

7.5 $$\begin{aligned} f\bowtie w \mapsto A_{(p,v)}^w \cdot B_{(p,v)}^f , \end{aligned}$$

for all $f\in \mathcal {A}^*$ and $w\in \mathcal {A}$, and by linear extension, on the whole $\mathcal {D}(\mathcal {A})$. Below we show that this map defines a representation of the Drinfled double $\mathcal {D}(\mathcal {A})$ (see also [BMCA13]). As the maps

$$\begin{aligned} f\bowtie \epsilon \mapsto B_{(p,v)}^f \quad \text {and} \quad \epsilon \bowtie w \mapsto A_{(p,v)}^w \end{aligned}$$

form representations of $\mathcal {A}^*$ and $\mathcal {A}$, respectively, and $(f\bowtie w) = (\epsilon \bowtie w) \cdot (f\bowtie 1)$, we only have to check that the commutation relation Eq. (7.4) holds. Let us therefore compare the action of $A_{(p,v)}^w\cdot B_{(p,v)}^f$ and the action of $B_{(p,v)}^f \cdot A_{(p,v)}^w$. The graphical representation of the action of the operator $A_{(p,v)}^w\cdot B_{(p,v)}^f$ is the following:

**Figure:**
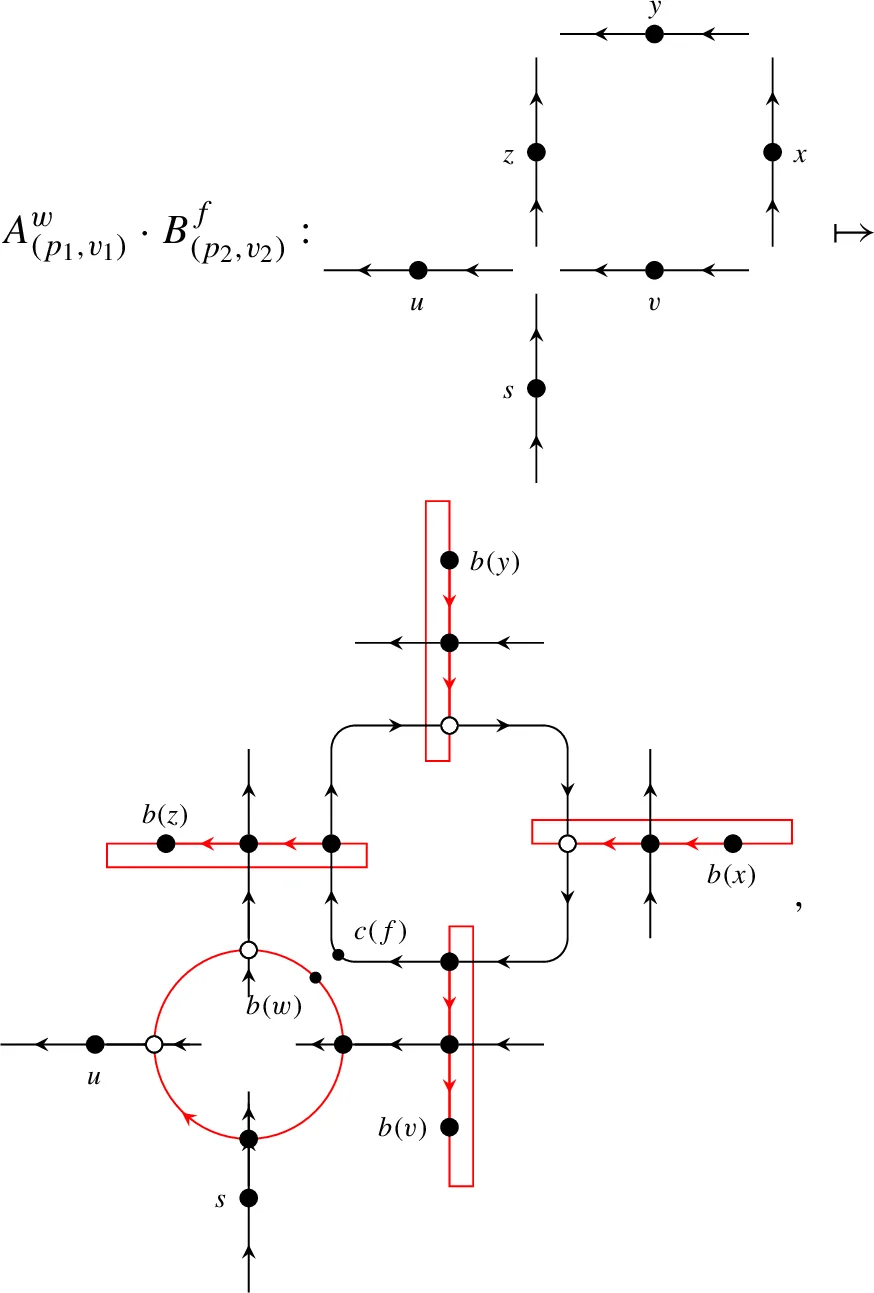

where *v* is the vertex with all four edges drawn, and *p* is the plaquette with all four bordering edges drawn. Let us now depict the action of the operator $B_{(p,v)}^f \cdot A_{(p,v)}^w$. To do that, note that as we act first with the operator $A_{(p,v)}^w$, the coproduct in the operator $B_{(p,v)}^f$ also applies to the MPO representation of *w*. The graphical representation of this action is thus

**Figure:**
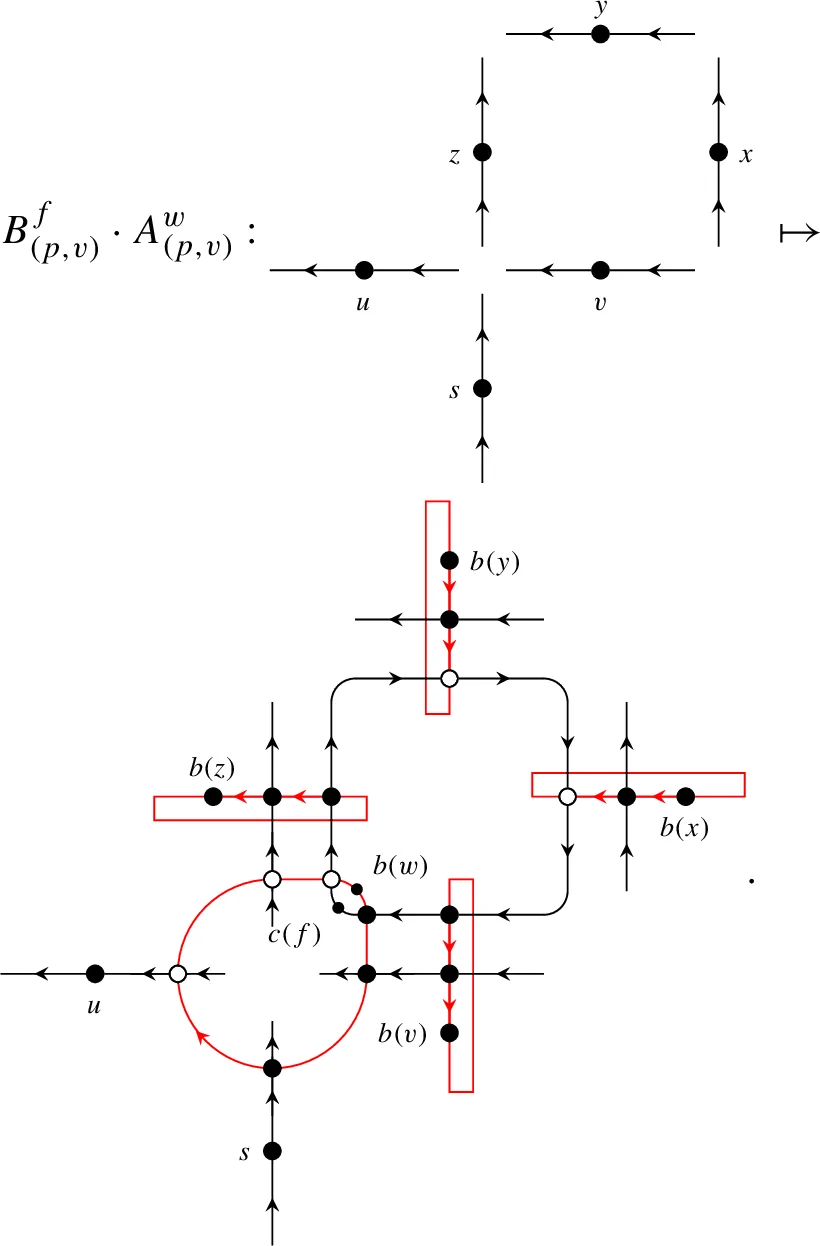

This operator describes the action of the operator

$$\begin{aligned} \sum _{(f)(w)} f_{(3)}\circ S( w_{(1)}) \cdot f_{(1)}(w_{(3)}) \cdot A_{(p,v)}^{w_{(2)}} \cdot B_{(p,v)}^{f_{(2)}}, \end{aligned}$$

and thus, as this commutation relation is the same^11^ as Eq. (7.4), the map in Eq. (7.5) describes a representation of the Drinfeld double $\mathcal {D}(\mathcal {A}^*)$.

Let us now construct a set of states that differ from $\vert \Psi \rangle$ only on a given pair of plaquette *p* and vertex *v*. The states, in the bulk, are given by

**Figure:**
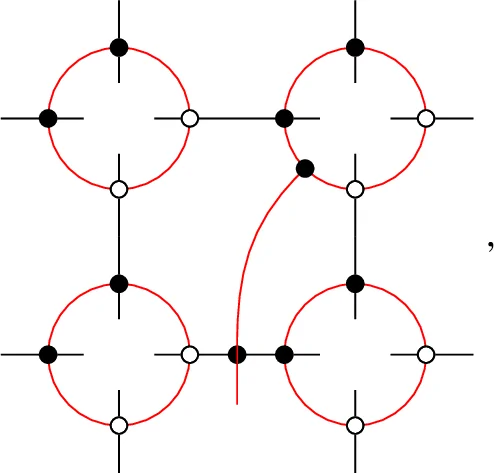

i.e. at the position defined by the vertex and plaquette we insert a rank-three tensor in $W \otimes \psi (\mathcal {A}) \subset W\otimes \textrm{End}(W)$, where *W* is the virtual vector space of the MPO and ψ is the corresponding representation of $\mathcal {A}^*$, and we continue with the MPO starting from the given point. If the state is defined by open boundary, then the boundary has one more index than the boundary describing the ground state $\vert \Psi \rangle$ of the PEPS. If the state is defined on closed boundary, then the string has to terminate at some point; at this termination we will insert another rank-three tensor the same way as above. That is, on closed boundary, we do not define a state with a single defect in it, instead, only states that differ from $\vert \Psi \rangle$ at least in two different positions (note, however, that one can construct periodic boundary states with an odd number of defects).

Both operators $B_{(p,v)}^f$ and $A_{(p,v)}^w$ map a state with a defect at the pair of plaquette *p* and vertex *v* to another state with a defect at the same position, but this state might be described by a different rank-three tensor. The action of the operators $B_{(p,v)}^f$ and $A_{(p,v)}^w$ on the rank-three tensor can be depicted as

**Figure:**
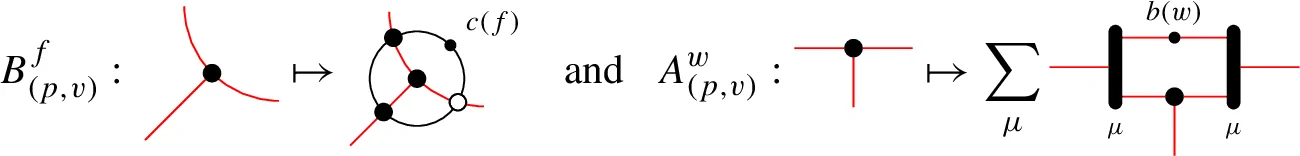

Or, by formulas, if the tensor is given by $\vert v \rangle \otimes b(x)$, then

$$\begin{aligned} B_{(p,v)}^f: \vert v \rangle \otimes b(x) \mapsto&\sum _{(f)} \psi (f_{(2)}) \vert v \rangle \otimes f_{(1)}(x_{(1)}) \cdot f_{(3)}\circ S(x_{(3)})\cdot b(x_{(2)}),\\&A_{(p,v)}^w: \vert v \rangle \otimes b(x) \mapsto \vert v \rangle \otimes b(xw). \end{aligned}$$

These states thus form a $\mathcal {D}(\mathcal {A}^*)$-module. We identify the *anyons* of the model as the irrep sectors of this module, i.e. an anyon is a set of states, each of which are locally different from the ground state of the Hamiltonian and such that the above defined local operators do not mix the different anyons. Note that as the Hamiltonian, in general, is not in the center of $\mathcal {D}(\mathcal {A}^*)$, an anyon might not have a definite energy (i.e. the different states in the anyonic sector might have different energies, see also [KL17]).

## Outlook and Applications

In this work we have introduced MPO representations of pre-bialgebras to describe extended symmetries of one-dimensional quantum systems. This formalism is equivalent to the categorical formulation, but uses instead an algebraic language to characterize the relevant MPOs. The construction of MPO symmetries in the categorical framework requires the knowledge of the *F*-symbols of a given bimodule category. In the algebraic framework the input is a concrete representation of a given weak Hopf algebra and its dual (which, in fact, carries the same information).

The difference in viewpoints manifests itself in the ease with which certain properties can be derived. For instance, in the categorical framework the pulling-through equations are derived from local relations between the tensors, whereas in the algebraic framework the pulling-through equations are satisfied by construction, and one has to derive the local relations between the tensors. Extracting the boundary matrix product density operators (MPDOs) within the algebraic framework becomes particularly transparent, since the relevant structural elements can be written down explicitly. Another application where the explicit form of these algebraic elements is crucial is the verification of the local topological quantum order (LTQO) condition [OPR25].

Finally, our algebraic framework can provide an advantage for future generalizations: for example, one can readily generalize the existing constructions to multi-fusion categories (non-biconnected $C^*$-weak Hopf algebras). The weak Hopf algebra framework, beginning already at the level of pre-bialgebras, naturally accommodates the generalizations to the non-semisimple case as well, and thus, to define meaningful MPO-injective PEPS, the task is to identify a pulling-through structure in those algebras. This broader applicability illustrates that the algebraic approach is not merely a reformulation of the fusion-category setting but a genuine generalization capable of treating models beyond the traditional Levin–Wen framework.

The algebraic and MPO-based framework developed here thus offers a flexible foundation for exploring generalized symmetries, locality structures, and fixed-point phenomena in quantum many-body systems. The structural insights gained from the framework may lead to applications such as in the analysis of one-dimensional mixed phases, renormalization group flows, matrix product unitaries, convergence of dissipative dynamics, etc. Broader applications such as the framework’s natural extension to the non-semisimple setting remain to be explored. In the following, we illustrate these possibilities in concrete settings, exemplifying both the present utility and the prospective reach of the approach.

*Operator-algebraic analysis of quantum many-body systems.* A first class of applications concerns problems requiring a rigorous description of superselection sectors for anyon excitations. Our algebraic framework allows an explicit description of the MPO symmetries of quantum double models together with the strings associated to the anyons. This can be used to construct the mathematical objects needed for their formulation within the operator-algebraic setting that handles such questions directly in the thermodynamic limit. Thus, the interplay between this operator-algebraic perspective and our framework—especially explicit tensor network representations—remains a promising direction to explore, including in higher dimensions.

Within the algebraic-operator setting, Haag duality has become a pivotal notion in the operator-algebraic analysis of quantum many-body systems. It plays a key role (i) in the description of anyonic excitations [Naa12, Oga22, BHNV25], which are widely believed to classify two-dimensional non-chiral gapped quantum phases of matter, (ii) in the von-Neumann algebraic formulation of entanglement and quantum information theory [LSWW24, LSSW24, LSWW25, LSW25b, LSW25a], and (iii) in the proof of the quantization of the Hall conductance [BCFO25]. Despite its importance, however, prior to [OPR25], Haag duality had only been rigorously established for Kitaev’s quantum double models with abelian input groups [Naa12, FN15]. The publication [OPR25] extends this to a large class of two-dimensional tensor network states, built from biconnected $C^*$-weak Hopf algebras. In that work, the tensor network methods developed in the present work are used in the construction of commuting parent Hamiltonians, to prove that they satisfy the LTQO condition, and in the proof of Haag duality itself. However, several aspects of the tensor network description of anyons remain open. In particular, describing fusion and braiding in this framework, and identifying the explicit operators that create anyon pairs, move, and braid them in terms of tensor networks, presents an interesting direction for future work.

*One-dimensional quantum mixed-state phases*

Another family of applications arises in the study of mixed-state phases of one-dimensional systems where our framework can be used to give explicit examples of matrix product density operators (MPDO). The fact that these MPDOs come from algebraic structures facilitates the study of their MPO symmetries, and the coproduct of the underlying coalgebra can be seen as a coarse-graining map that allows one to study renormalization group flows on these mixed states. Finally, these MPDOs arise naturally as the boundary states of the topological PEPS that we have constructed. Altogether, our algebraic MPO representation provides a powerful framework to study mixed-state phases and tackle their classification.

Two seminal papers formalizing the study of mixed-state phases are [CPSV17a], which defines renormalization fixed point (RFP) MPDOs, and [CP19], which defines the notion of phases for mixed states. The algebraic viewpoint is particularly useful in these settings. For example, the papers [RGMP24, Sun25, LRS+25], building on the techniques developed in the present paper, explore the resulting phase diagram. First, in [RGMP24] RFP MPDOs and explicit renormalization maps were constructed based on MPO representations of $C^*$-weak Hopf algebras. It has been proven, moreover, that these states arise as boundaries of two-dimensional topologically ordered states and it was shown that the states arising from (non-weak) $C^*$-Hopf algebras are in the trivial phase. In [Sun25], the author proves that MPDOs exhibiting strong anomalous MPO symmetries, encompassing both unitary and non-invertible symmetries, are not in the trivial phase according to the definitions above (see [LCW25] as well). In [LRS+25], RFP MPDOs are interpreted as steady states of local frustration-free Lindbladians with minimal steady-state degeneracy, i.e., explicit local Lindbladians are constructed for which the steady states correspond to the sectors of the RFP MPDO. This result shows that RFP MPDOs are physical, justifying their use in phase classification.

*Renormalization group flow for mixed states.* The framework is also expected to shed light on renormalization problems where one must determine whether a given mixed state is a genuine renormalization fixed point and, if so, which algebraic structures organize its coarse-graining flow. In such contexts, pre-bialgebras naturally encode the stability properties of MPDOs under renormalization. For example, in [Kat24], a renormalization step is defined as the application of a circuit of local quantum channels that has an inverse of the same form. Unlike matrix product states, which always admit a well-defined isometric renormalization transformation, it is shown that general MPDOs do not necessarily possess a convergent exact renormalization group flow. However, a subclass of MPDOs that does admit a well-defined flow is identified, and these MPDOs can be understood as representations of certain pre-bialgebras, as formalized in the present work. Determining precisely when such convergence occurs, which algebraic structures arise in full generality, and how these structures can be interpreted physically remains an open direction for future research.

*Circuit complexity of matrix product unitaries.*

Matrix product unitaries (MPUs) are many-body unitaries that, despite their tensor network structure limiting entanglement generation, remain more general than quantum cellular automata and can still produce long-range entanglement. They can describe discrete time evolution, unitary symmetries of 1D systems, and the boundary physics of Floquet phases in 2D. The techniques introduced in this paper may pave the way for a systematic investigation and classification of such unitaries. Two notable advances have already been made in this direction, both building on the results presented here. First, [STC25] shows that a broad class of MPUs—constructed from representations of $C^*$-weak Hopf algebras—can be implemented with polynomial-depth quantum circuits. Second, [Wan25] introduces a new family of exactly solvable unitary circuits describing quantum many-body dynamics in discrete space and time. This dynamical evolution can be compressed into an MPU whose bond dimension is time-independent, enabling exact computation of the full evolution from any matrix product state. Notably, the work analyzes a model built from a weak Hopf algebra closely related to a Floquet version of the PXP model, suggesting that results of the present paper might shed new light on quantum many-body scars and, more broadly, on Floquet dynamics in constrained systems.

*Non-semisimple generalizations.* Finally, the present formalism extends to non-semisimple or non-cosemisimple pre-bialgebras, capturing non-semisimple categorical symmetries. This extension may enable the identification of new phases of matter in one- and two-dimensional systems that exhibit such symmetries. A concrete case requiring departure from the semisimple setting is the anomalous $\mathbb {Z}_2$ symmetry of the XX model. Despite its simplicity, this symmetry lies outside the framework of fusion categories, yet it is accommodated in our approach, yielding a pre-bialgebra that is semisimple but not cosemisimple [LMS+25]. Symmetries that are neither semisimple nor cosemisimple may lead to further exotic phases of matter.

## Data Availability

Data sharing is not applicable to this article as no new data were created or analyzed in this study.

## Proof of Lemma 5.1 and of Lemma 5.2

Before proving Lemma 5.1, we need the following lemma:

### Lemma A.1

In a cosemisimple WHA, the following holds for all $a,b,c\in \textrm{Irr}(\mathcal {A}^*)$:  where the constants $w_c$ and $w_a$ are defined in Eq. (5.24).

### Proof

Using Eq. (5.24), one can write  where in the second equality we have used that $N_{dc}^{r_c} = N_{r_c\bar{c}}^{d}$ (see Eq. (5.23)) and that $r_c = l_{\bar{c}}$ (see Theorem 5.3), and thus that the sum over μ on the r.h.s. runs from 1 to $N_{dc}^{r_c} = N_{r_c\bar{c}}^{d} = N_{l_{\bar{c}}\bar{c}}^{d} = \delta _{d\bar{c}}$, i.e. it is an empty sum if $d\ne \bar{c}$ and it consists of a single term if $d=\bar{c}$. Due to associativity (Eq. (4.6)), the r.h.s. can be further rewritten as  where in the first equation we have used that $N_{\bar{a}d}^e = N_{ae}^d$ (see Eq. (5.23)), and that it is non-zero for e∈E if and only if $e=r_{a}$, and in this case it is $\delta _{da}$ (see Eq. (5.9)); in the second equation we have used again Eq. (5.24). Finally note that $N_{\bar{a}\bar{b}}^{\bar{c}} = N_{\bar{b}c}^{a}$ is non-zero only if $r_{c} = r_a$, and thus one can drop the prefactor $\delta _{r_cr_a}$. Rearranging the resulting equation leads to the desired result. □

Let us now restate and prove Lemma 5.1:

### Lemma 5.1

Let $\mathcal {A}$ be a cosemisimple WHA, and *g* be the linear functional defined in Eq. (5.25). Then there exists an $N_{ab}^c\times N_{ab}^c$ matrix $B_{ab}^c$ such that the linear functional $g\in \mathcal {A}^*$ satisfies  Moreover, $\left( B_{ab}^c\right) ^2 = \textrm{Id}_{N_{ab}^c}$ and the following equation holds as well:

### Proof

Let us apply Eq. (5.22) three times. The first application yields  The second application yields  Finally, the third application yields  We have thus obtained that there exists an invertible matrix $Y_{ab}^c$ such that  and in fact, $Y_{ab}^c = C_{\bar{b}\bar{a}}^{\bar{c}} \hat{C}_{b\bar{c}}^{\bar{a}} C_{\bar{a}c}^b$. Similarly, by three consecutive applications of the inverse relations of Theorem 5.2,  we conclude that there is an invertible matrix $X_{ab}^c$ such that  In fact, $X_{ab}^c = (C_{\bar{a}c}^b)^{-1} (\hat{C}_{b\bar{c}}^{\bar{a}})^{-1} (C_{\bar{b}\bar{a}}^{\bar{c}})^{-1}$, i.e. $X_{ab}^c Y_{ab}^{c} = \textrm{Id}$. Using similar arguments, we conclude that there are invertible matrices $\hat{X}$ and $\hat{Y}$ such that  Just as above, $\hat{X}_{ab}^c \hat{Y}_{ab}^c = \textrm{Id}$. Moreover, by orthogonality of the fusion tensors, $\hat{Y}_{ab}^c = (Y_{ab}^c)^T$:  where in the first equality we have used the definition of $Y_{ab}^c$ and the orthogonality of the fusion tensors, while in the second the definition of $\hat{Y}_{ab}^c$ and again the orthogonality of the fusion tensors. By a similar argument, we obtain $\hat{X}_{ab}^c = (X_{ab}^c)^T$ as well.

By definition of the linear functional *g*, we obtain that  We have thus obtained that Eq. (5.31) holds with

A.3 $$\begin{aligned} B_{ab}^c = \frac{d_ad_b}{d_c} \frac{w_{\bar{c}}}{w_{\bar{a}}w_{\bar{b}}} \cdot \hat{Y}_{ab}^c X_{\bar{b}\bar{a}}^{\bar{c}} . \end{aligned}$$

Combining the two equations in Eq. (5.31), we obtain that  On the other hand, using Lemma A.1 three times, we obtain that  or equivalently, as $(d_a/w_{\bar{a}})^2 = w_{a}/w_{\bar{a}}$, that  This equation can be rearranged as  and therefore, comparing this equation to Eq. (A.4), we obtain that $\left( B_{ab}^c\right) ^2 = \textrm{Id}$. Finally, using Eq. (A.3) and then Lemma A.1, we can write  Part of the last statement follows using that $d_a/w_{\bar{a}} = w_a/d_a$. Finally, using again Lemma A.1, we conclude that □

Let us restate and prove now Lemma 5.2:

### Lemma 5.2

For all $a\in \textrm{Irr}(\mathcal {A}^*)$, $d_a^2 = w_a w_{\bar{a}}>0$. Let moreover $T_{ab}^c$ be defined by $T_{ab}^c = \sum _{\mu } \left( B_{ab}^c\right) _{\mu \mu }$. Then the following equations hold:

$$\begin{aligned} \sum _b T_{ab}^c \cdot d_b = d_a \cdot \delta _{l_a l_c} \cdot d_c \quad \text {and} \quad \sum _{x:l_x = l_a} d_x^2 = \sum _{x:l_x =r_a} d_x^2. \end{aligned}$$

### Proof

Using Theorem 5.2,  Therfore, using Lemma 5.1, we obtain that $B_{ab}^c$ can be expressed as

A.5 $$\begin{aligned} B_{ab}^c = w_a \cdot \frac{d_c}{d_a d_b} \cdot \left( C_{\bar{a}c}^b\right) ^T \left( C_{ab}^c\right) ^{-1}. \end{aligned}$$

As $B_{ab}^c$ squares to the identity, the eigenvalues of $B_{ab}^c$ are ±1, and thus $T_{ab}^c$, the trace of $B_{ab}^c$, is an integer with $|T_{ab}^c|\le N_{ab}^c$. Note now that Eq. (A.5) can be used to obtain the following expression for $B_{\bar{a}c}^b$:

$$\begin{aligned} B_{\bar{a}c}^b = w_{\bar{a}} \cdot \frac{d_b}{d_a d_c} \left( C_{ab}^c\right) ^{T} \left( C_{\bar{a}c}^b\right) ^{-1} = C_{\bar{a}c}^b \cdot \left[ \left( B_{ab}^c\right) ^{-1}\right] ^T \cdot \left( C_{\bar{a}c}^b\right) ^{-1} = C_{\bar{a}c}^b \cdot \left( B_{ab}^c\right) ^T \cdot \left( C_{\bar{a}c}^b\right) ^{-1}, \end{aligned}$$

where in the first equation we have used that $d_a = d_{\bar{a}}$, in the second equation we have used that $w_{\bar{a}} d_b / (d_ad_c) = (w_{a} d_c /(d_a d_b))^{-1}$ and in the third that $B_{ab}^c$ squares to the identity. Taking the trace of the two sides in this equation, we obtain that $T_{\bar{a}c}^b = T_{ab}^c$. By a similar argument, we obtain that $T_{ab}^c = T_{c\bar{b}}^a$ as well. The definition of $T_{ab}^c$ and Lemma 5.1 implies that  Using Eq. (5.24), the fact that $d_a^2 = w_a w_{\bar{a}}$, and the counit axiom Eq. (5.12), the r.h.s. can be further written as  Therefore the equation $\sum _b T_{ab}^c d_b = d_a \cdot \delta _{l_a l_c} d_c$ holds.

Following the proof of Theorem 2.3 in [EGH+] (see also [Bar15]), we can now prove that $d_a^2 = w_a w_{\bar{a}}$ is a positive number. Notice first that the matrix $T_a^T T_a = T_{\bar{a}} T_a$ is positive semidefinite (as $T_a$ is a real matrix), and thus all of its eigenvalues are non-negative. Let us show that $d_a^2 = w_a w_{\bar{a}}$ is one of its eigenvalues, then, as neither $w_a$ nor $w_{\bar{a}}$ is 0, this implies the the positivity of $d_a^2$. To see that it is an eigenvalue, we can check that $\delta _{r_a l_b}d_b$ is the corresponding eigenvector:

$$\begin{aligned} \sum _{bc} T_{\bar{a}c}^d T_{ab}^c \delta _{r_a l_b} d_b =\sum _{bc} T_{\bar{a}c}^d T_{ab}^c d_b = d_a \sum _c T_{\bar{a}c}^d \delta _{l_a l_c} d_c = d_a \sum _c T_{\bar{a}c}^d d_c = d_a d_{\bar{a}} \delta _{r_a l_d} d_d, \end{aligned}$$

where in the first equation we have used that $T_{ab}^c = 0$ if $\delta _{r_al_b} = 0$ (as then $N_{ab}^c=0$ and $|T_{ab}^c| < N_{ab}^c$); the same relation is used in the third equation, together with $r_{\bar{a}} = l_a$. In the last equation we have used $r_a = l_{\bar{a}}$. Finally note that $d_{\bar{a}} = d_a$ and that the vector defined by $\delta _{r_a l_b} d_b$ is non-zero.

Finally, let us prove that $\sum _{x:l_x = l_a} d_x^2 = \sum _{x:l_x =r_a} d_x^2$. For that, note that $T_{ab}^c = T_{\bar{a}c}^b = T_{b\bar{c}}^{\bar{a}} = T_{\bar{b}\bar{a}}^{\bar{c}}$, and thus

$$\begin{aligned} d_a \sum _{x:l_x = l_a} d_x^2 = \sum _{xb} T_{xb}^a d_x d_b = \sum _{xb} T_{\bar{b}\bar{x}}^{\bar{a}} d_x d_b = \sum _{\bar{x}\bar{b}} T_{\bar{b}\bar{x}}^{\bar{a}} d_{\bar{x}} d_{\bar{b}} = d_{\bar{a}} \sum _{\bar{b}:l_{\bar{b}} = l_{\bar{a}}} d_{\bar{b}}^2 = d_a \sum _{x:l_x = r_a} d_{x}^2 . \end{aligned}$$

As $d_a\ne 0$, the statement follows. □

## Example: String-Net Models

In the following, we create WHAs from fusion categories that appear in the construction of string-net models. For simplicity, we will restrict to fusion categories where the fusion multiplicities are all 0 or 1, $N_{ab}^c \le 1$ for all *a*, *b*, *c*. With this assumption, the pentagon equations for the *F*-symbols simplify to the equation:

B.1 $$\begin{aligned} \left[ F_{fcd}^e\right] _l^g \left[ F_{abl}^e\right] _k^f = \sum _h \left[ F_{abc}^g\right] _h^f \left[ F_{ahd}^e\right] _k^g \left[ F_{bcd}^k\right] _l^h. \end{aligned}$$

The MPO tensor used in the construction of the string-net models is then defined as  where the dotted lines serve as a visual reminder of the δ prefactors. We will often make use of this visual reminder of the δ prefactors and only write the non-zero components of the MPO tensor,  Each line is labeled by the simple objects of the category, i.e. it is an *N*-dimensional vector space (*N* is the number of simple objects in the fusion category); thus the bond dimension of this tensor is $N^3$. This bond dimension can be reduced as the tensor is block diagonal and it contains a zero block. The projectors reducing the tensor (those that correspond to a non-zero block) are  These are indeed projectors as $N_{fl}^e\in \{0,1\}$ and it is easy to see that they commute with the MPO tensor (the *F*-symbols satisfy $\left[ F_{abl}^e\right] _k^f=0$ unless $N_{fl}^e\ne 0$ and $N_{bl}^k\ne 0$). The bond dimension of each block is then $D_l = \sum _{fe} N_{fl}^e$. A similar decomposition holds for the vertical direction as well. There the projectors that decompose the MPO tensor are  Starting from this MPO tensor, let us define a linear space $\mathcal {A}$ as  This linear space $\mathcal {A}$ has a natural coalgebra structure, with the coproduct given by  As above, taking repeated coproducts of a coalgebra element is the same as growing the size of the MPO. In particular, the operation Δ defined by this equation is associative. Note that as the pentagon equation Eq. (B.1) can be rearranged as

$$\begin{aligned} \left[ F_{ahd}^e\right] _k^g \left[ F_{bcd}^k\right] _l^h = \sum _f \left[ F_{fcd}^e\right] _l^g \left[ F_{abl}^e\right] _k^f \left[ \left( F_{abc}^g\right) ^{-1}\right] _f^h, \end{aligned}$$

the coproduct can also be expressed as  where we have defined the fusion tensors of the physical level as  As the construction is built on a fusion category, there is a unique vacuum label that we denote by 1. The counit of this coalgebra is given by  where we have used that $\left[ F_{1bl}^k\right] _{k}^b = \alpha _b/\alpha _k$ independent of *l* and we have defined

Let us now show that $\mathcal {A}$ is not only a coalgebra, but it also has a pre-bialgebra structure. For that, we define fusion tensors corresponding to this MPO tensor as  These tensors satisfy the associativity equations Eq. (4.6), because the equations  both follow from the pentagon equation Eq. (B.1). More precisely, the left equation is exactly Eq. (B.1), while the right equation is its inverse,

$$\begin{aligned} \left[ \left( F_{fcd}^e\right) ^{-1}\right] _g^l \left[ \left( F_{abl}^e\right) ^{-1}\right] _f^k = \sum _h \left[ \left( F_{abc}^g\right) ^{-1}\right] _f^h \left[ \left( F_{ahd}^e\right) ^{-1}\right] _g^k \left[ \left( F_{bcd}^k\right) ^{-1}\right] _h^l. \end{aligned}$$

We can now check for the key equation Eq. (4.8). The r.h.s. of Eq. (4.8) is  where we have only written out the components that are not automatically zero due to the delta functions. This equation is the pentagon equation rearranged:

$$\begin{aligned} \left[ F_{abc}^g\right] _h^f \left[ F_{ahd}^e\right] _k^g = \sum _l \left[ F_{fcd}^e\right] _l^g \left[ F_{abl}^e\right] _k^f \left[ \left( F_{bcd}^k\right) ^{-1}\right] _h^l. \end{aligned}$$

Let us now check for the l.h.s. of Eq. (4.8), i.e. the orthogonality of the fusion tensors:  where again $N_{fl}^e$ is one or zero, which gives the r.h.s. of Eq. (4.8). We have thus checked that $\mathcal {A}$ admits a pre-bialgebra structure with the usual multiplication, except that we have not shown that $\mathcal {A}$ has a unit. For that, note that the unit is of the form  where we have defined  As the unit is described by a rank-one boundary that is supported only in the vacuum sector, the pre-bialgebra $\mathcal {A}$ automatically satisfies the unit axiom. Dually, as the counit is also described by a rank-one boundary that is supported only in the vacuum sector, $\mathcal {A}$ also satisfies the counit axiom. Therefore $\mathcal {A}$ is a WBA.

Let us now show that $\mathcal {A}$ is a WHA as well. A cosemisimple WBA is a WHA if and only if for every irrep sector *a* there is another irrep sector $\bar{a}$ such that the symmetries $N_{ab}^c = N_{\bar{a}c}^b$ and $N_{ab}^c = N_{c\bar{b}}^a$ hold. In our case, as the fusion multiplicities originate from a fusion category, and thus these symmetries trivially hold. Therefore $\mathcal {A}$ is a WHA. The matrices $Z_c$ and $Z^{-1}_c$ describing the antipode can be expressed with the help of the fusion tensors as Eq. (5.24):  It is easy to see that these matrices are the inverses of each other. The square of the antipode is realized by the matrices

### Fibonacci anyons

The fusion rules are given by:

$$\begin{aligned} N_{11}^1 = N_{\tau 1}^\tau = N_{1\tau }^\tau = N_{\tau \tau }^1 = N_{\tau \tau }^1 =&1, \\ N_{11}^\tau = N_{\tau 1}^1 = N_{1\tau }^1 =&0. \end{aligned}$$

The F-symbol $\left[ F_{fcd}^e\right] _l^g$ is proportional to $N_{fc}^g \cdot N_{cd}^l \cdot N_{fg}^e \cdot N_{ld}^e$. Therefore the following entries are the only non-zero ones and are given by

$$\begin{aligned} \left[ F_{111}^1\right] _1^1 = \left[ F_{11\tau }^\tau \right] _\tau ^1 = \left[ F_{\tau 11}^\tau \right] _1^\tau = \left[ F_{1\tau 1}^\tau \right] _\tau ^\tau = \ \left[ F_{1\tau \tau }^1\right] _1^\tau = \left[ F_{1\tau \tau }^\tau \right] _\tau ^\tau = \\ \left[ F_{\tau 1\tau }^\tau \right] _\tau ^\tau = \left[ F_{\tau 1\tau }^1\right] _\tau ^\tau = \left[ F_{\tau \tau 1}^\tau \right] _\tau ^\tau = \left[ F_{\tau \tau 1}^1\right] _\tau ^1 = \left[ F_{\tau \tau \tau }^1\right] _\tau ^\tau =1 \\ \left[ F_{\tau \tau \tau }^\tau \right] _1^1 = \varphi , \ . \left[ F_{\tau \tau \tau }^\tau \right] _\tau ^\tau = -\varphi , \ \left[ F_{\tau \tau \tau }^\tau \right] _1^\tau = \left[ F_{\tau \tau \tau }^\tau \right] _\tau ^1 = \sqrt{\varphi }, \end{aligned}$$

where $\varphi = (\sqrt{5}-1)/2$. We also have $\left( \left( F_{abc}^d\right) ^{-1}\right) _e^f=\left( F_{abc}^d\right) _f^e$.

#### The MPO tensor

The MPO tensor given in Eq. (B.2) has bond- and physical dimension 8. It is block-diagonal both in the horizontal and vertical direction, with the two blocks labeled by 1 and τ. The block 1 corresponds to the subspace spanned by $\vert 111 \rangle , \vert \tau 1 \tau \rangle$ and the block τ to the subspace spanned by $\vert 1\tau \tau \rangle , \vert \tau \tau 1 \rangle , \vert \tau \tau \tau \rangle$. The two simple cocommutative elements are given by

$$\begin{aligned} a_1&\equiv \left( \begin{matrix} \left[ F_{111}^1\right] _1^1 & \left[ F_{111}^\tau \right] _\tau ^1 \\ \left[ F_{1\tau 1}^1\right] _1^\tau & \left[ F_{1\tau 1}^\tau \right] _\tau ^\tau \\ \end{matrix}\right) \oplus \left( \begin{matrix} 0 & 0& 0 \\ 0 & 0 & 0 \\ 0 & 0 & \left[ F_{\tau \tau 1}^\tau \right] _\tau ^\tau \\ \end{matrix}\right) = \left( \begin{matrix} 1 & 0 \\ 0 & 1 \\ \end{matrix}\right) \oplus \left( \begin{matrix} 0 & 0& 0 \\ 0 & 0 & 0 \\ 0 & 0 & 1 \\ \end{matrix}\right) \\ a_\tau&\equiv \left( \begin{matrix} \left[ F_{11\tau }^1\right] _1^1 & \left[ F_{11\tau }^\tau \right] _\tau ^1 \\ \left[ F_{1\tau \tau }^1\right] _1^\tau & \left[ F_{1\tau \tau }^\tau \right] _\tau ^\tau \\ \end{matrix}\right) \oplus \left( \begin{matrix} 0 & 0& 0 \\ 0 & 0 & 0 \\ 0 & 0 & \left[ F_{\tau \tau \tau }^\tau \right] _\tau ^\tau \\ \end{matrix}\right) = \left( \begin{matrix} 0 & 1 \\ 1 & 1 \\ \end{matrix}\right) \oplus \left( \begin{matrix} 0 & 0& 0 \\ 0 & 0 & 0 \\ 0 & 0 & -\varphi \\ \end{matrix}\right) , \end{aligned}$$

where the basis is ordered as given above. The square of the antipode, as given by Eq. (b.3), is implemented by

## Footnotes

1 Note that even if $\mathcal {C}^*$ arises as the group algebra $\mathbb {C}[G]$ of some finite group *G*, we are talking about the representation of the group algebra, and not the representation of the group; that is, we require the map $\phi : \mathbb {C}[G]\rightarrow \textrm{End}(V)$ to be injective, not the map $\phi |_G: G\rightarrow \textrm{End}(V)$.

2 Remember that the unit of $\mathcal {A}^*$ is ϵ

3 Note that as ϵ is a projector, $V_a\otimes V_b = \textrm{Ker}\{(\psi _a \boxtimes \psi _b)(\epsilon )\} \oplus \textrm{Im}(\psi _{a}\boxtimes \psi _{b})(\epsilon )$. The extension of the operators $Y_{ab}^{c\mu }$ is such that they act as zero on the space $\textrm{Ker}\{(\psi _a \boxtimes \psi _b)(\epsilon )\}$.

4 Not necessarily unital; note that the unit of the algebra $1\in \mathcal {A}$ might have a non-trivial boundary, and thus, in general, it is not in the Grothendieck ring.

5 The same result can be obtained by direct calculation as well [Nik03]: using the unit axiom Eq. (5.1) twice, $\Delta (\tau _E) = \sum _{(1)}1_{(1)}1_{(3)} \otimes 1_{(2)}1_{(4)} =\sum _{(1)} \Delta (1) \cdot (1_{(2)} \otimes 1_{(1)} 1_{(3)}) = \Delta (1) \cdot \Delta _{\textrm{op}}(1) \cdot \Delta (1)$. Finally, again due to the unit axiom, $\Delta (1)\cdot \Delta _{\textrm{op}}(1) = \Delta _{\textrm{op}}(1)\cdot \Delta (1)$, that leads to the desired result.

6 the order of the permutation $a\mapsto \bar{\bar{a}}$

7 We could have chosen $\lambda Z_c$ to describe the antipode instead of $Z_c$, for any $\lambda \in \mathbb {C}$. This is in fact the only freedom that we have in the choice of $Z_c$.

8 Remember that ϕ is w.l.o.g. injective and thus the value of $\phi ^{\otimes n} \circ \Delta ^{n-1} (\Lambda )$ defines Λ uniquely.

9 As Ω is non-degenerate, *T* is uniquely defined, see Sect. 6.

10 by pseudo-graph we mean a graph where multiple edges are allowed between vertices.

11 remember that $\mathcal {A}$ is a $C^*$-Hopf algebra, and thus $S^{-1} = S$
